# In Silico Analysis of Two Hard Tick P450s: Identification, Characterization, and Putative Metabolism of *Cymbopogon citratus* Essential Oil Constituents

**DOI:** 10.3390/ijms26178489

**Published:** 2025-09-01

**Authors:** Caishan Li, Licui Wen, Wenyu Shi, Yuqian Deng, Na Zhou, Xueqing Zhao, Qingyong Guo, Bayinchahan Gailike

**Affiliations:** 1 College of Veterinary Medicine, Xinjiang Agricultural University, Urumqi 830052, China; 320200058@stu.xjau.edu.cn (C.L.); 320240083@stu.xjau.edu.cn (L.W.); 320222767@stu.xjau.edu.cn (W.S.); 320232830@stu.xjau.edu.cn (Y.D.); 320232901@stu.xjau.edu.cn (N.Z.); 320232820@stu.xjau.edu.cn (X.Z.); 2 Xinjiang Key Laboratory of New Drug Research and Development for Herbivores, Urumqi 830052, China

**Keywords:** ticks, p450, monoterpene, molecular docking

## Abstract

The cytochrome p450 gene family is widely involved in various biological processes in arthropods. Tick p450s are often associated with chemical acaricides, but knowledge of their involvement in the metabolism of plant-derived essential oil components is limited. In this study, we identified the non-redundant number of p450 transcripts (NRNPTs) from *Haemaphysalis longicornis* and *Hyalomma asiaticum* under the *Cymbopogon citratus* essential oil (CCEO) and terpinolene stress using de novo transcriptome data, respectively. In this study, we identified and characterized the NRNPTs of *Ha. longicornis* and *Hy. asiaticum*. Their gene expression patterns and biological functions under CCEO and terpinolene stress were further analyzed. Finally, *Hy. asiaticum* NRNPTs (87) were more numerous than *Ha. longicornis* (58). Phylogenetic analyses showed that NRNPTs of both *Hy. asiaticum* and *Ha. longicornis* could be categorized in clan 2, clan 3, clan 4, and clan mito, this data comes from the NRNPTs. Phylogenetic analyses showed that NRNPTs of both *Hy. asiaticum* and *Ha. longicornis* could be categorized in clan 2, clan 3, clan 4, and clan mito. p450 members of both were most distributed in clan 3. In addition, one *Hy. asiaticum* NRNPT was identified as belonging to the new classification clan 20 (*Hyas*CYP20A1). The biological functions and pathways of p450 family members enriched in *Hy. asiaticum* and *Ha. longicornis* under different exogenous substance stresses were different, and the expression patterns of these genes were inconsistent. Molecular docking results showed that *Ha. longicornis* p450 members (*Halo*CYP3A4 and *Halo*CYP4B1), which were significantly up-regulated under CCEO stress, as well as *Hy. asiaticum Hyas*CYP24A1 and *Hyas*CYP4V2 (the *Halo*CYP3A4 and *Halo*CYP4B1 homologous genes), encode proteins that differ in their ability to metabolize CCEO components, but they all bind well to Germacrene D and naphthalene. Our study enriches the knowledge of the involvement of p450 family members of different tick species in the metabolism of essential oil components of plants, and provides a theoretical basis for further in-depth studies on the function of tick p450 enzymes.

## 1. Introduction

Ticks are globally distributed obligate hematophagous ectoparasites that serve as vectors for diverse pathogens, including bacteria, viruses, and protozoa [1,2,3]. These pathogens substantially contribute to the global burden of tick-borne diseases (TBD), with estimated annual economic losses reaching USD 72 billion [4,5]. *Haemaphysalis longicornis* and *Hyalomma asiaticum* (Arachnida: Ixodidae) are the dominant tick species in China, with their primary distribution encompassing Pakistan, Iran, Turkey, and East Asia [6,7]. *Hy. asiaticum* acts as a biological vector for multiple pathogens, including bunyaviruses and rickettsiae [8]. It plays a key role in transmitting various diseases, such as Crimean-Congo hemorrhagic fever [9], North Asia tick-borne spotted fever, tick-borne encephalitis [10], and theileriosis [11]. Similarly, *Ha. longicornis* is a major vector species, associated with the transmission of severe fever with thrombocytopenia syndrome virus [12], *Anaplasma marginale* [13], and *Ehrlichia* species [14].

Tick population control represents a fundamental strategy for reducing tick-associated damage. While diverse control strategies have been developed [15,16,17], chemical acaricides remain the predominant approach for reducing tick populations in practical applications [18]. However, prolonged and excessive use of chemical acaricides constitutes the primary driver of emerging acaricide resistance in ticks [19]. Furthermore, concerns persist regarding chemical residues and their adverse environmental and ecological impacts [20]. Plant-derived extracts have emerged as promising alternatives to conventional acaricides, owing to their favorable eco-friendly profiles. Numerous botanical essential oils and plant-derived natural products have demonstrated significant acaricidal efficacy against ticks [21,22,23]. Plant extracts and essential oils employed for tick control are rich in diverse secondary metabolites, including terpenoids, phenols, and alkaloids [24], among which terpenoids constitute the predominant components [25]. For example, the monoterpene derivative pyrethroid has been used successfully and extensively for pest control [26,27]. However, there is now a consensus that many pests are resistant to pyrethroid. This means that it is necessary to explore and discover other monoterpenes that can be used for pest control. Nowadays, terpenoid-based formulations have demonstrated significant acaricidal activity against ticks. For instance, the acaricidal properties of CCEO were primarily attributed to its terpenoid constituents [28,29]. Thymol exhibits effective standalone activity against *Rhipicephalus microplus* (Arachnida: Ixodidae) [30], whereas carvacrol and eugenol display synergistic efficacy against *Rhipicephalus sanguineus* (Arachnida: Ixodidae) [31]. The monoterpenoids carvacrol and thymol have been associated with detoxification metabolism in *R. microplus* larvae [32]. Nevertheless, these studies had focused on evaluating the efficacy of plant-derived acaricides, and there is very limited information on how the tick organism is involved in the mechanism of action of plant extracts/essential oil components, especially the main component, terpenoids.

Cytochrome p450 (p450) is widely distributed in plants, animals, and microorganisms [33]. In insects, p450 enzymes fulfill diverse physiological roles, encompassing hormone biosynthesis and degradation, fatty acid metabolism, pheromone and defensive compound synthesis, as well as the metabolism of both endogenous and exogenous substances [34]. It has become common knowledge that essential oils or insecticides introduce changes in p450 enzyme activity [35,36]. The p450 gene family exhibits significant variation in member numbers among different species [37]. According to classical phylogenetic classification, insect p450 genes are categorized into four major clans: clan 2, clan 3, clan 4, and the mitochondrial clan [38]. Notably, an additional clan, clan 20, has been identified in ticks [38]. p450 family members play a well-documented role in the metabolic detoxification of chemical acaricides. For example, upregulation of CYP3006G8 and CYP41 in *R. microplus* has been associated with resistance to deltamethrin [39], whereas p450-mediated cross-resistance to cyenopyrafen and pyridaben has been reported in *Tetranychus urticae* (Order: Trombidiformes, Family: Tetranychidae) [40]. Furthermore, insect p450s are implicated in the metabolism of plant-derived natural compounds [41]. For example, *Sitophilus zeamais* (Order: Coleoptera, Family: Curculionidae) CYP6MS1 may be a key enzyme in metabolizing terpinene-4-ol [42].

To the best of our knowledge, the mechanisms by which cytochrome p450 family members in ticks mediate the metabolism of plant essential oil compounds remain poorly characterized. Existing studies have demonstrated the acaricidal potential of *Cymbopogon citratus* essential oil and terpinolene [8,43]. In this study, we systematically characterized the NRNPTs in *Ha. longicornis* and *Hy. asiaticum* using de novo transcriptomic analyses under exposure to CCEO and terpinolene, respectively. Additionally, we employed molecular docking approaches to elucidate the potential roles of p450 enzymes in metabolizing bioactive components of CCEO. Our findings provide novel molecular insights into the metabolic pathways of plant-derived acaricides and establish a theoretical reference for developing eco-friendly strategies for tick control.

## 2. Results

### 2.1. Identification and Phylogenetic Analysis of the NRNPTs

**Figure 1:**
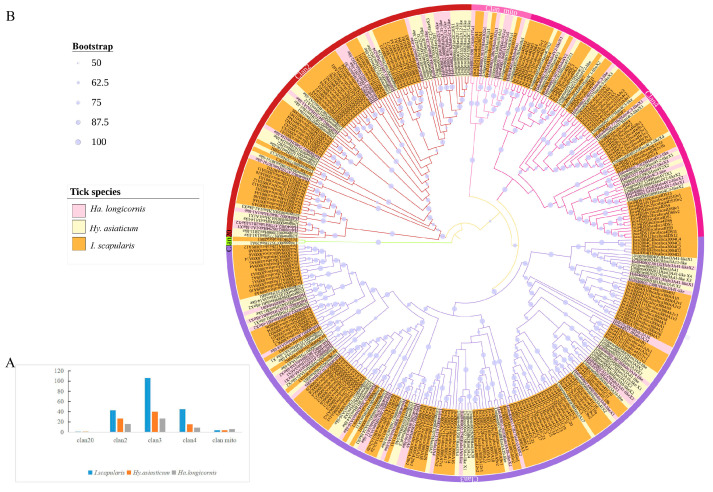
Identification and phylogenetic analysis of the NRNPTs members in *Hy. asiaticum*, *Ha. longicornis*, and p450 gene family of *I. scapularis*. (**A**) Distribution profiles of the NRNPTs members among three tick species. (**B**) In the phylogenetic tree, sequence labels are color-coded by species origin: *Ha. longicornis*, *Hy. asiaticum*, and p450 gene family of *I. scapularis*.

A total of 145 NRNPTs were identified in *Hy. asiaticum* and *Ha. longicornis* (File S1, Table S1). In *Ha. longicornis*, 58 NRNPTs were classified into clan 2 (16), clan 3 (27), clan 4 (4), and mitochondrial clan (4). In *Hy. asiaticum*, 87 p450 genes were distributed among clan 2 (27), clan 3 (40), clan 4 (15), clan 20 (1), and the mitochondrial clan (4), with clan 3 being the most abundant. Comparative analysis revealed that clan 3 contained the highest number of NRNPTs (27 in *Ha. longicornis* and 40 in *Hy. asiaticum*). Notably, *Ha. longicornis* exhibited a greater number of mitochondrial clan NRNPTs than *Hy. asiaticum* and *Ixodes scapularis* (Family: Ixodidae) (Figure 1A). Overall, both *Hy. asiaticum* and *Ha. longicornis* possessed NRNPTs from clan2, clan3, clan4, and clan mito, exhibiting distinct clustering patterns and high sequence homology. Phylogenetic analysis further revealed that *Hy. asiaticum* CYP20A1 clustered closely with *I. scapularis* CYP20R1, sharing the highest sequence homology (Figure 1B).

### 2.2. Function Annotation and Classification

#### 2.2.1. GO Annotations and Enrichment Analysis

**Figure 2:**
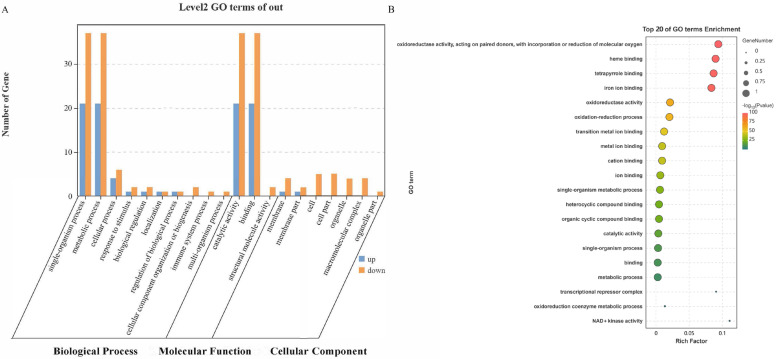
Functional annotation of cytochrome NRNPTs in *Ha. longicornis* based on GO analysis. Note: (**A**) Level 2 GO terms associated with the cytochrome p450 family in *Ha. longicornis*. (**B**) Top 20 enriched GO terms for the cytochrome p450 family in *Ha. longicornis*.

Gene Ontology (GO) enrichment analysis of *Ha. longicornis* NRNPTs under CCEO stress identified significant level 2 terms in 10 biological processes, 6 cellular components, and 2 molecular functions (Figure 2A). The predominant biological processes included metabolic process (GO: 0008152) and single-organism process (GO: 0044699). Molecular functions were primarily associated with catalytic activity (GO:0003824) and binding activity (GO:0005488). Cellular components were enriched for membrane-related terms, specifically membrane parts (GO:0044425) and integral membrane components (GO:0016020). The functional distribution of *Ha. longicornis* NRNPTs was primarily associated with biological processes and molecular functions (Figure 2A). GO enrichment analysis of the top 20 terms revealed which *Ha. longicornis* NRNPTs were mainly involved in oxidoreductase activity, acting on paired donors, with incorporation of reduction of molecular oxygen. And heme-binding, tetrapyrrole binding, and iron ion binding (Figure 2B).

**Figure 3:**
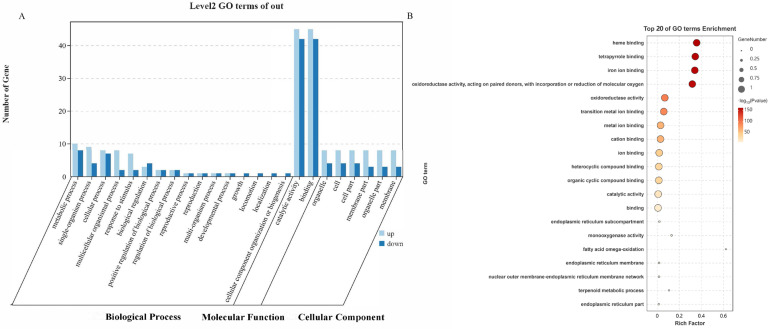
Functional annotation of the cytochrome p450 genes in *Hy. asiaticum* based on GO analysis. Note: (**A**) Level 2 (GO terms associated with the p450 family in *Hy. asiaticum)*; (**B**) The top 20 enriched GO terms of the p450 family in *Hy. asiaticum*.

Similarly, GO enrichment analysis was performed to categorize the differentially expressed *Hy. asiaticum* NRNPTs under terpinolene stress. The analysis revealed significant enrichment across 16 biological processes, 2 cellular components, and 6 molecular functions (Figure 3A). Among the biological processes, the most predominant categories were metabolic processes (GO:0008152), single-organism processes (GO:0044699), and cellular processes (GO:0009987). For molecular function, the highest enrichment was observed in catalytic activity (GO:0003824) and binding (GO:0005488). Cellular components were primarily associated with cell (GO:0005623), organelle (GO:0044422), and cellular part (GO:0044464). Notably, *Hy. asiaticum* NRNPTs were associated with biological processes, molecular functions, and cellular components. GO functional enrichment analysis revealed that the most significantly enriched terms were heme-binding, followed by tetrapyrrole binding (a structural component of porphyrin and heme-related compounds) and iron ion binding (Figure 3B).

#### 2.2.2. KEGG Annotations and Enrichment Analysis

**Figure 4:**
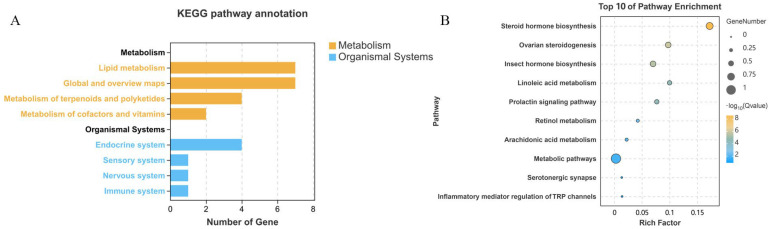
KEGG pathway annotation of the NRNPTs in *Ha. longicornis*. Note: (**A**) KEGG pathway enrichment analysis of the NRNPTs in *Ha. longicornis*. (**B**) Top 10 enriched pathways of the *Ha. longicornis* p450 family.

KEGG pathway enrichment analysis of *Ha. longicornis* NRNPTs under CCEO stress revealed significant enrichment in metabolism and organismal systems. Prominently enriched metabolic categories comprised global and overview maps (KO01100), lipid metabolism (KO00140 and KO00590), terpenoid biosynthesis (KO00981), and cofactor/vitamin metabolism (KO00830). Among these, the global and overview maps category contained the highest number of genes (Figure 4A), while the primary organism system involved was the endocrine system (KO04927). Further analysis illustrated that *Ha. longicornis* p450 family members were predominantly enriched in steroid hormone biosynthesis, ovarian steroidogenesis, and insect hormone biosynthesis pathways (Figure 4B).

**Figure 5:**
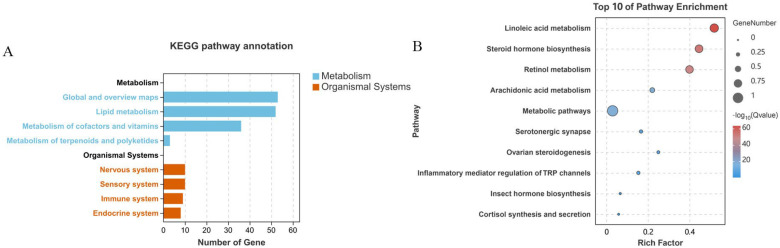
KEGG pathway enrichment analysis of the NRNPTs in *Hy. asiaticum.* Note: (**A**) KEGG pathway enrichment analysis of the NRNPTs in *Hy. asiaticum*; (**B**) The top 10 significantly enriched KEGG pathways associated with differentially expressed NRNPTs in *Hy. asiaticum*.

KEGG pathway enrichment analysis of *Hy. asiaticum* NRNPTs under terpinolene stress identified metabolism and organismal system pathways (Figure 5A). The main metabolic categories included global and overview maps (ko01100), lipid metabolism (ko00591), cofactor and vitamin metabolism (ko00830), and terpenoid and polyketide metabolism (ko00981), with the global and overview maps containing the highest number of genes. Among the organism systems, the nervous system (ko04726) and sensory system (ko04750) were most prominently represented. KEGG pathway enrichment analysis revealed that p450 family members of *Hy. asiaticum* were mainly enriched in linoleic acid metabolism, steroid hormone biosynthesis, and retinol metabolism pathways (Figure 5B).

### 2.3. Expression Profiles of Cytochrome p450 Genes in Response to Potential Plant-Derived Acaricides

**Figure 6:**
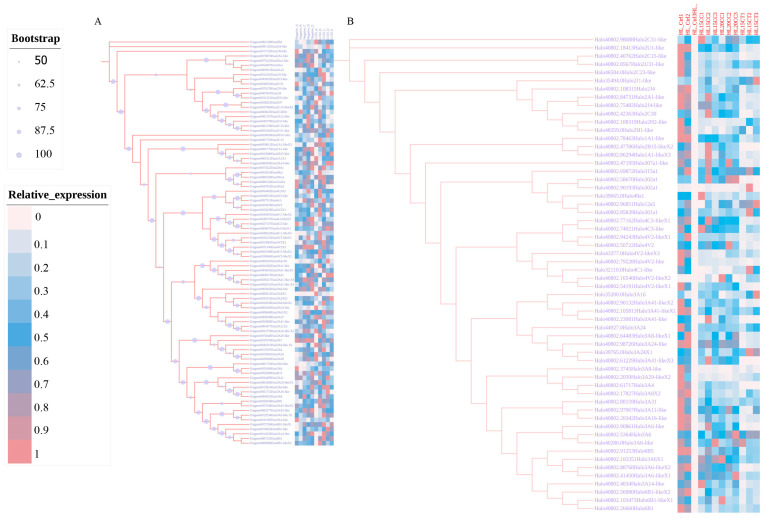
Expression heatmap profiles of NRNPTs members in *Hy. asiaticum* and *Ha. longicornis* under potential plant-derived acaricide exposure. Note: (**A**) Expression heatmap profiles of NRNPTs members in *Hy. asiaticum*; (**B**) Expression heatmap profiles of NRNPTs members in *Ha. longicornis*.

We analyzed the expression patterns of all NRNPTs members in *Ha. longicornis* and *Hy. asiaticum* under the stress of CCEO and terpinolene, respectively. Obviously, distinct expression patterns were observed in response to the stress of CCEO and terpinolene, respectively (Figure 6). Specifically, exposure to CCEO significantly upregulated *Ha. longicornis* CYP3A4 (*Halo*CYP3A4) and CYP4B1 (*Halo*CYP4B1). Homologous genes of *Halo*CYP3A4 and *Halo*CYP4B1 were found in the *Hy. asiaticum* transcriptome under terpinolene stress: *Hy. asiaticum* CYP24A1 (Unigene00086134, *Hyas*CYP24A1) and CYP4V2 (Unigene0091089, *Hyas*CYP4V2).

### 2.4. Cloning of Hy. asiaticum p450 Genes

The complete CDSs of *Hyas*CYP24A1 and *Hyas*CYP4V2 were cloned as homologous genes of *Ha. longicornis Halo*CYP3A4 and *Halo*CYP4B1. Sequence analysis revealed that *Hyas*CYP24A1 spans 1629 bp, encoding a 543-amino acid protein, while *Hyas*CYP4V2 comprises 1473 bp, encoding a 491-amino acid protein. The amplified fragments were verified by agarose gel electrophoresis, and the confirmed sequences have been deposited in the NCBI GenBank database under accession numbers PQ806966 (*Hyas*CYP4V2) and PQ806965 (*Hyas*CYP24A1).

### 2.5. Molecular Docking Analysis

**Figure 7:**
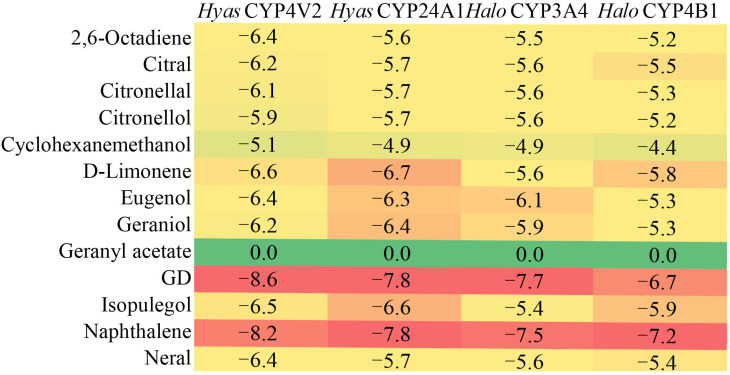
Heat map of the p450 family of *Hy. asiaticum* and *Ha. longicornis*.

Molecular docking analysis was performed to assess the binding interactions between 13 major constituents of CCEO and potential metabolizing p450 members (*Halo*CYP3A4, *Halo*CYP4B1, *HyasC*YP24A1, *HyasC*YP4V2). The results revealed differential binding affinities between the various essential oil components and the corresponding p450 proteins. Notably, germacrene D (GD) (a monoterpene) and naphthalene exhibited the strongest binding interactions with all investigated p450 isoforms (Figure 7).

**Figure 8:**
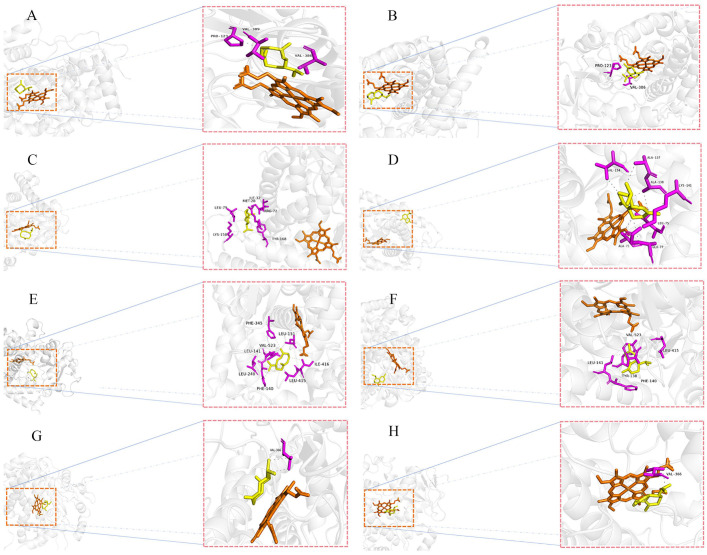
Molecular docking analysis of the p450 enzymes from *Hy. asiaticum* and *Ha. longicornis* with GD and naphthalene. Note: (**A**) Molecular docking analysis of the *Halo*CYP3A4-GD complex; (**B**) Molecular docking analysis of the *Halo*CYP3A4-naphthalene complex; (**C**) Molecular docking analysis of the *Halo*CYP4B1-GD complex; (**D**) Molecular docking analysis of the *Halo*CYP4B1-naphthalene complex; (**E**) Molecular docking analysis of the *Hyas*CYP4V2-GD complex; (**F**) Molecular docking analysis of the *Hyas*CYP4V2-naphthalene complex; (**G**) Molecular docking analysis of the *Hyas*CYP24A1-GD complex; (**H**) Molecular docking analysis of the *Hyas*CYP24A1-naphthalene complex.

The molecular docking heatmap result exhibited strong binding affinities between GD/naphthalene and the potential metabolizing proteins (*HyasC*YP4V2, *HyasC*YP24A1, *Halo*CYP3A4, *Halo*CYP4B1). Therefore, these two compounds were selected for detailed molecular docking analyses with the potential metabolizing proteins (Figure 8). The docking results revealed that hydrophobic interactions were the predominant binding mode between the GD/naphthalene and amino acid residues of the potential metabolizing proteins. *HaloC*YP3A4-GD complex bound with residues of Pro-123, and Val-386, and Val-389 (Figure 8A). *Halo*CYP3A4-naphthalene complex bound with residues of Pro-123, and Val-386 (Figure 8B). *Halo*CYP4B1-GD complex bound with residues of Met-28, Ile-32, Arg-72, Leu-79, Lys-158, and Tyr-168 (Figure 8C). *Halo*CYP4B1-naphthalene complex bound with residues of Ala-71, Leu-75, Glu-77, Val-134, Ala-137, Ala-138, and Lys-141 (Figure 8D). *Hyas*CYP4V2-GD complex bound with residues of Phe-140, Leu-141, Leu-151, Leu-248, Phe-345, Leu-415, Ile-416, and Val-523 (Figure 8E). *Hyas*CYP4V2-naphthalene complex bound with residues of Tyr-138, Phe-140, Leu-141, Leu-145, and Val-523 (Figure 8F). *Hyas*CYP24A1-GD complex bound with residues of Val-366 (Figure 8G). *Hyas*CYP24A1-naphthalene complex bound with residues of Val-366 (Figure 8H).

## 3. Discussion

The cytochrome p450 gene family exhibits remarkable diversity and complexity in ticks. For instance, 206 p450 genes were identified in the *I. scapularis* genome, a substantially higher number than observed in other insects [44]. This extensive p450 gene family complexity poses significant challenges for accurate gene identification, classification, and functional characterization. Current research indicates that p450 enzymes in insects are primarily involved in phase I detoxification processes. Although their specific functional roles remain incompletely understood [39], there are several findings that point to the involvement of the tick p450 family in the metabolism of natural products and chemical insecticides [8,39]. In this study, we systematically identified and characterized the NRNPTs in *Ha. longicornis* and *Hy. asiaticum* using transcriptomic data under potential plant-derived acaricide exposure.

Phylogenetic analysis and identification of the NRNPTs revealed that both *Hy. asiaticum* and *Ha. longicornis* possess members distributed across clan 2, clan 3, clan 4, and clan mito. Clan 2 is functionally associated with endogenous compound metabolism and biosynthetic pathways [45]. Among these, clan 3 represents the largest p450 group, exhibiting the highest gene count and family membership. This clan demonstrates remarkable evolutionary activity within insect genomes, characterized by extensive polymorphisms and pronounced tissue specificity [46]. Notably, clan 3 p450 genes participate in detoxification processes and the metabolism of diverse exogenous compounds, facilitating insect adaptation to environmental stressors. Our analysis identified a substantially greater number of clan 3 p450 genes in *Hy. asiaticum* (40 genes) compared to *Ha. longicornis* (27 genes). Clan 4 primarily functions in detoxification and pheromone metabolism, while clan mito is involved in fatty acid, sterol, and hormone metabolism [47]. Intriguingly, our study reports the novel identification of a clan 20, *Hy. asiaticum* CYP20A1, marking its first discovery in this species. This finding aligns with previous reports of clan 20 members in the *I. scapularis* genome [38].

GO and KEGG enrichment analyses provided insights into the functional roles and pathways of p450 genes under potential plant-derived acaricide stress. The GO analysis revealed that p450 genes from different tick species exhibit distinct functions in metabolizing exogenous substances. However, common biological features—such as catalytic activity and binding [48]—were observed among candidate p450 family members across these tick species.

KEGG pathway annotation revealed that the p450 genes from *Ha. longicornis* and *Hy. asiaticum* were primarily involved in metabolic and organismal systems pathways. Subsequent analysis identified the top 10 enriched pathways, among which seven were shared between the two species. Notably, the up-regulated gene *Halo*CYP3A4 and its homologous counterpart *Hyas*CYP24A1 were associated with the insect hormone biosynthesis pathway. In contrast, *Halo*CYP4B1 and *Hyas*CYP4V2 were not detected among the top 10 enriched pathways. These findings suggest that members of the p450 gene family play complex roles in regulating normal physiological processes or responding to exogenous stimuli in ticks [38]. Furthermore, p450 enzymes implicated in metabolizing plant-derived exogenous compounds, such as pesticides, did not exhibit distinct phylogenetic clustering [45].

In general, the significant upregulation of p450 gene expression is closely linked to their role in exogenous substance metabolism [49,50,51]. Therefore, we analyzed the expression profiles of the NRNPTs in two tick species under exposure to potential plant-derived acaricides. Our results revealed distinct expression patterns between tick species and stress conditions, with notably not all p450 genes being transcriptionally active. We hypothesize that the upregulated genes may represent key candidates involved in p450-mediated metabolic responses to exogenous stressors. Notably, we identified two *Ha. longicornis* p450 gene candidates (*Halo*CYP3A4 and *Halo*CYP4B1) that exhibited significant upregulation upon exposure to CCEO. We propose that the encoded proteins of *Halo*CYP3A4 and *Halo*CYP4B1 may function in detoxifying bioactive components of this essential oil. Our previous study found that *Hyas*CYP3A8 was implicated in terpinolene metabolism [8], while *Tribolium castaneum* (Order: Coleoptera, Family: Tenebrionidae) CYP6BQ8 contributed to terpinen-4-ol detoxification [52]. Collectively, these observations suggest that different terpenes may be metabolized by specific p450 genes, likely due to their genetic diversity, broad substrate specificity, and catalytic versatility [53].

Further evaluation of the ability of *Ha. longicornis* and *Hy. asiaticum* p450 members to metabolize constituents of CCEO based on the molecular docking method. Building upon these molecular docking results, we delve deeper into their biochemical significance, particularly concerning the implications for complex stability or potential metabolic outcomes. The predominance of hydrophobic interactions in the binding of GD and naphthalene to all investigated P450 isoforms is a key finding with significant biochemical implications [54,55]. Hydrophobic interactions are major contributors to the stability of protein-ligand complexes. The burial of non-polar ligand moieties within hydrophobic pockets of the enzyme’s active site reduces the entropic penalty associated with water molecules ordering around these surfaces, thereby stabilizing the complex [56]. The extensive hydrophobic contacts observed, particularly for complexes like HyasCYP4V2-GD (involving residues Phe-140, Leu-141, Leu-151, Leu-248, Phe-345, Leu-415, Ile-416, Val-523) and HaloCYP4B1-naphthalene (Ala-71, Leu-75, Glu-77, Val-134, Ala-137, Ala-138, Lys-141), suggest a tight fit within largely non-polar environments. This high degree of surface complementarity typically correlates with stronger binding affinity and potentially enhanced complex stability [57]. The effect on the stability of molecular docking still needs to be verified by experimental techniques such as molecular dynamics (MD) simulations.

We took into consideration that CCEO is not used against *Hy. asiatiucm*, and *Hy. asiatiucm* is the dominant species in Xinjiang, China. So, we successfully cloned *Hyas*CYP24A1 and *Hyas*CYP4V2 (they are *Halo*CYP3A4 and *Halo*CYP4B1 homologous genes, respectively) as receptors to estimate the feasibility of *Hyas*CYP24A1 and *Hyas*CYP4V2 metabolizing the constituents of CCEO. Overall, *Ha. longicornis* (*Halo*CYP3A4 and *Halo*CYP4B1) and *Hy. asiaticum* (*Hyas*CYP24A1 and *Hyas*CYP4V2) p450 family members involved in the metabolism of 13 components of CCEO differed in their binding affinity. Terpenoids are widely studied compounds in plant tick control strategies and, likewise, are one of the components in essential oils [24]. Terpenoids are mainly repellents, food -deterrents, and toxic to pest control [58]. In the present study, *Halo*CYP3A4 and *Halo*CYP4B1 did not show strong binding to terpenoids from CCEO, suggesting that there are other specific p450 members responsible for metabolizing these terpenoids in *Ha. longicornis*. However, the proteins encoded by *Halo*CYP3A4 and *Halo*CYP4B1, as well as their homologous genes *Hyas*CYP24A1 and *Hyas*CYP4V2 found in *Hy. asiaticum*, are well-bound by GD and naphthalene. Surprisingly, the proteins encoded by *Halo*CYP3A4 and *Halo*CYP4B1 both bind well to GD and naphthalene. It suggests that stimulation by GD and naphthalene as exogenous substances is a key molecular factor leading to the response of *Ha. longicornis* p450 members. GD, a class of sesquiterpenes widely found in plant essential oils, possesses insecticidal, and insect pheromone functions [59]. Naphthalene is a good repellent for wheat stem sawfly and *Cephus cinctus* [60]. Therefore, GD and naphthalene can be considered as effective compounds for tick control. Of course, perhaps other ingredients from CCEO could be included. Because, this paper only evaluates the metabolism of the p450 family enzymes of *Ha. longicornis* and *Hy. asiaticum* on CCEO components, and did not focus on the characterization of the action of CCEO components with their molecular targets.

## 4. Materials and Methods

### 4.1. Identification of CYP450: The Non-Redundant Number of Transcripts

The acquisition of transcriptome data after terpinolene stress in unfed nymph *Hy. asiaticum* references our previous study [8]. However, unlike the prior approach, de novo assembly was employed in this study (data unpublished). Additionally, unigene, peptide, and gene expression data for CCEO and citronellal-stressed unfed adult *Ha. longicornis* were retrieved from the National Center for Biotechnology Information (NCBI) Gene Expression Omnibus (GEO) database (accession number: GSE176275) [43]. To identify p450 candidates in *Hy. asiaticum* and *Ha. longicornis*, we firstly retrieved p450 sequences of *Drosophila melanogaster* (Order: Diptera, Family: Drosophilidae), *Aedes aegypti* (Order: Diptera, Family: Culicidae), *Pseudophyllum aegypti* (Order: Orthoptera, Family: Tettigoniidae), and *Centruroides sculpturatus* (Family: Buthidae) from the NCBI database as query sequences. These query sequences were used to mine potential p450 peptides in both tick species using BLAST+ (*E* < 0.05). In parallel, p450 candidates were also identified through a hidden Markov model (HMM) search (PF00067) using HMMER v3.1 (Loudoun County, VA, USA) (*E* < 0.05). All candidate peptides from both methods were merged and subsequently submitted to the NCBI Conserved Domain Database (CDD) for complete p450 domain validation.

### 4.2. p450: The Non-Redundant Number of Transcripts Phylogenetic Analysis

The final candidate p450 members from *Hy. asiaticum* and *Ha. longicornis*, which contained a complete p450 domain, along with the p450 non-redundant number of transcripts of members of *I. scapularis*, were subjected to phylogenetic analysis. Multiple sequence alignment was performed using MAFFT v4.748, followed by sequence trimming with TrimAl v1.2 (parameter: gappyout). The optimal substitution model was determined, and a maximum likelihood (ML) phylogenetic tree was constructed using IQ-TREE v2.2.2.6 (Vienna, Austria). The resulting tree was visualized using iTOL v7.2.1 (European Molecular Biology Laboratory, Meyerhofstraße 1, Heidelberg, Germany).

### 4.3. NRNPTs Expressive Profile Analysis Under the Abiotic Stress

The input data for the analysis of NRNPTs differential expressive profile in the de novo transcriptome data of *Hy. asiaticum* under terpinolene were the reads count data obtained from the analysis of gene expression levels, which were analyzed using DESeq2 v1.48.1 software (Chapel Hill, NC, USA) [61]. Specific analyses included (1) normalization of the read count; (2) calculation of the probability of hypothesis testing (*p*-value) according to the model; and (3) finally, correction for multiple hypothesis testing to obtain the FDR value (false discovery rate). The screening criteria for significant differential genes were *p-*value < 0.05 and |log_2_(fold change)| > 1. The differentially expressed NRNPTs in the de novo transcriptome of CCEO-stressed *Ha. longicornis* were derived from those described in reference [40].

### 4.4. GO and KEGG Functional Analysis of NRNPTs

Further explore the biological functions of two types of ticks under abiotic stress. GO and KEGG functional analysis of two types of tick NRNPTs was performed using OmicShare Tool (https://www.omicshare.com/ accessed on 18 November 2024) Specifically, we used NRNPTs containing annotation information (including log2FC) as the target file and GO and KEGG annotation information files for all transcripts in the transcriptome as the background file to perform functional analysis of NRNPTs in two types of ticks.

### 4.5. Complete CDS Cloning for the Metabolism of CCEO Components by Hy. asiaticum

BLAST v2.12.0 (Bethesda, MD, USA) was used to search for homologous genes in the terpinolene-stressed *Hy. asiaticum* de novo transcriptomes that were significantly up-regulated in the p450 genes after stress in *Ha. longicornis* with terpinolene [8]. Subsequently, we cloned the complete coding sequences (CDS) of two differentially expressed p450 genes. For *Hy. asiaticum* CYP4V2 (F: 5′-ACAGCGTTCCTAATGCCAGA-3′; R: 5′-CTTGCAAAGCAGCGTTATGT-3′), PCR (Bio-Rad Laboratories, Inc., Hercules, CA, USA) amplification was conducted under the following conditions: initial denaturation at 95 °C for 3 min; 35 cycles of denaturation at 95 °C for 15 s, annealing at 59.8 °C for 15 s, and extension at 72 °C for 1 min; followed by a final extension at 72 °C for 5 min. Similarly, for *Hy. asiaticum* CYP24A1 (F: 5′-CGAAGTTGTTTAGTTGGGCG-3′; R: 5′-CAGGTGAGGGAAGAGGATGC-3′), the amplification protocol consisted of initial denaturation at 95 °C for 3 min; 35 cycles of denaturation at 95 °C for 30 s, annealing at 57 °C for 30 s, and extension at 72 °C for 45 s; with a final extension at 72 °C for 3 min.

### 4.6. Molecular Docking

This study investigated the metabolic mechanism of CCEO (CCEO) mediated by cytochrome p450 (CYP450) enzymes in *Ha. longicornis* and *Hy. asiaticum* ticks using computational molecular docking approaches. The 3D structures of CCEO constituents were retrieved from the PubChem database as ligand molecules. CYP450 proteins exhibiting differential upregulation in *Ha. longicornis* under CCEO exposure, along with their orthologs in *Hy. asiaticum*, were selected as receptor targets. Homology modeling was performed by identifying suitable templates through BLAST searches against the Protein Data Bank (PDB), followed by structure prediction using SWISS-MODEL (Basel, Switzerland). Molecular docking simulations were conducted employing CB-Dock2 (Chengdu, China) [62], with subsequent visualization and analysis performed using PyMOL v2.5.0 (Schrödinger, Inc., New York, NY, USA).

## 5. Conclusions

In this study, we conducted a preliminary exploration of the genetic characterization of the NRNPTs of *Ha. longicornis* and *Hy. asiaticum* and evaluated their ability to metabolize constituents of CCEO by means of de novo transcriptomic data under CCEO or terpinolene stress. In summary, the number of NRNPTs in *Hy. asiaticum* was greater than in *Ha. longicornis*. NRNPTs expression patterns under different exogenous substance stresses with different biological functions. The proteins encoded by *Halo*CYP3A4 and *Halo*CYP4B1 (as well as the *Hyas*CYP24A1 and *Hyas*CYP4V2 genes, which are homologous to them), which were significantly up-regulated under CCEO stress, may be key enzymes for potential metabolism of GD and naphthalene. Other components of CCEO may be metabolized by other p450s from *Ha. longicornis*, but the related NRNPTs expression did not undergo significant up-regulation under CCEO stress. Our findings enrich the molecular mechanisms by which the tick p450 family metabolizes plant-derived acaricides and provide new insights for further studies on the biological functions of the tick NRNPTs.

## Data Availability

The original contributions presented in this study are included in the article/Supplementary Material. Further inquiries can be directed to the corresponding author(s).

## Supplementary Materials

The following supporting information can be downloaded at: https://www.mdpi.com/article/10.3390/ijms26178489/s1.
