# Supplementary material for: In Silico Analysis of Two Hard Tick P450s: Identification, Characterization, and Putative Metabolism of Cymbopogon citratus Essential Oil Constituents
Source: Int J Mol Sci. 2025 Sep 1;26(17):8489. doi: 10.3390/ijms26178489 (PMC12429676; doi:10.3390/ijms26178489)
Supplement: Supplementary file 1 [file ijms-26-08489-s001.zip › File S1.pdf]

File S1 Sequence information of *Haemaphysalis longicornis* and *Hyalomma asiaticum* p450 family members identified in this study

Haemaphysalis longicornis p450 sequences

>Halo40802.18413

MEWANIRDSRAAGVVGNPYLLTLGLVPLLTVAAVLLRKWLSSVRLPPGTRLPPMPPASSI

RGHAEVVSFGFHRKKALEWAEQLGPVIRLKWNFSDIVVLNDFKTVKQFMNTKQILDRSHC

FLLKREYYSGVGSVNGDTWNANRKFCLGMLRDLGFAKTAMEDNMMEEFSHLEKRIGNTKG

VPINVQEYIQPCALNNIASFLYGKRLPIDHPDRRQLQRLVAKLYWPLLKGPIQLFLPRLL

RWILERLPFTRLGQVNACMKDLEKFNEKQIQIHLKDATADVSDDFIQNYVKKIDEAKKES

KPLFTDRNLVGNVTGFLLAGTLSTASTVYWNLLINATNPDTIQSRVQKEIDDVVGTERSP

TWEDRKRMPFTLACIWEVDRWKTGSPLGVARECSEDVVIGEYFIPKGTTVLPNLWAIHND

PTFWKDPEKFDPARFLNQDGSIVSPKPEQLLPFSIGRRSCPGEMFALMEVFLMTTFLLQK

YRVVPDRAVKINLNSPDLMVPQQVPVKLQFLPRHTNIS

>Halo40802.98088

MGSTAAVVLSLTFAPVIWFTLRYLWRHLSRRQLPPGARLPPMPPASSIWGHVEMQTFNFF

RKKALEWAGMCGPVYRLKLNGCDIVVLNDFDSIKKFLNTKEILCRAQRFEVGPVYFPGVG

DINGERWNANRRFCMTMLRDLGFAKTAMEEKMMEEFAYLEETIGKTNGAPIDVKKYILSC

TSGNVASFFYGSRLPPSHSSRKELQTLITDICAHMHSASLLLFLPRPLFWFLGHLPFTKT

GQLKACMIALEKFTEKQILDYKADEEVDVSQDFIHGYLRKIEETKEEPHSMFTHQHLVGN

VNVFIMAGTISTTVTMWWLLCLFAKHPDTIQAKIQREIDDVVGPDRRPTWEDRKQMPFTL

ASISEVERWKTSTPLGLARESSDDVVIDGYFIAKGTTVIPNFWAVHNNPTHWKDPLEFNP

HRFLNAEGTITSQKPEHLIPFSLGRRSCPGEMFASMEIYLMVTFLLQKYNVVPERPIDCD

LNNPELLQPKQVDIRLQFLPRNRCNDGKFLPRTA

>Halo40802.69872

MSSAALARRLARQAAAGAGRRARSTEAAAAAGCPFHRVLQQEQSPSAGGVPEQHRAGGTA

AQPFSSLPRPKGLPVLGTALDVLRAGGATKIHEYCDRRHRELGPIYRETLGSVDAVFVAD

SALIQKVYTNEGKFPMHMVPEAWLIYNEVKGIQRGLFFMDGPEWIERRRNLNNVFLKPKT

VTDNVPVFNDVITDLLQRWHQARDSSGVLENVERELYNWSIESLGAMIFGRRLGCIAQTS

SMDNVHEFVYCVQQIFNESANMSMISPRVAYRLRLPVWRRFVKAAGRALELATDYVKENV

ADITKDNNNRRRFNQGILSQLLLNEKMSETELIRIVTDLFLAAADTTSHATQWALYLLAK

HPEQQERLLHAVRSVVPAGQPINEDSLARLPYVKAVIREALRLYPVAPFLTRILSEDIVL

GGYHIPAGKLILMSLYTTGRDERHFSEPNKFRPERWLRDRDRSGQVNSWACLPFGLGARS

CIGRRVAEVQMQFLIARTVQQFQLTPSTDKDVQIKMRLITTPEEPISLRMTPR

>Halo40802.50722

MRRSLFALGRKSSAERKAPTTAARAAQKRQFYAYAHKANITPAWRIPASDDPAMAAYDTM

LLSAPTAGSSQAATLVLLALLVAVLAKIIVVLARWLRMYWCLRNVPHPAQRWPFSLAVEM

WLSMAKMDPNLELTAKIFNYFDGMFRTIHDQDVTVAYYGPQPFLIAATPKAVESLLTNNT

NLNKAFLYKMMRPWIGNGILMIEKEKWRSRRKLITPAFHFRVLDDYAPIMNRRARELVRR
LDTMVGKGFFDVLPAVRLAAFGILFETALGVQIDEAEVQRMRLLEINDEIGASVIARMLN
LAHWFDTIYNLTQASKDFRRNIKFIHEYNKRIVKQRLAEYKQGKVGADSKKSFLDILLHM
HMVDGTLTEEEVKNEVTSIFIGGFETTASSITFTLFLLGNHPEVQEKVQEEIDALFADDR
DRDVTIEDIKQLKYLECVVKESMRLYPPVPLIARNVDEDMKVGEFTIPKGAVAVAAIYFI
QRHPKFFDEPDTFVPERFLDTKEKNPFLYVPFSGGFRNCLGQKFANLEDKILLTQIMRCF
TVTSKLRMEQLQLSIEVVLKATQGIEIQLHPRDKSPRPQ
>Halo40286.0
MWELVVATVVLFLVHWLLKRREHFNFFKNLGIPGPQPNIIFGNILEVYKKSPTKAYREWI
DTYGDVVGYFNGYRPVILVADLELLKQIQIKDFQDFADRGLLFQAKRPPSPHNKSLLQLT
STRWKEVRSVLTPSFTTTKLKMMSPGVVEAVQKLVSKIDRKAQSGEEFEAGEMYAALALD
VICKSAMGIDYNLQDHPRHGFLVCCRLLFGCAFSFIAVILTAFPGLASLLKFINMRWLRY
QNNGVHPFLEVQEKCKRIVAQRQQDSSLRQKDLLQLMIDAKEARMDMGSVTSSQLTAGDD
NEQELPTADANDSARLDTSVFKKAVLDDDDITQNAFVVLVAGFETTSNTMAMVTHMLSHH
PEVQEKVREELLSVLSPDEPITYSTIQKLTYMNCVIQETMRMYPPAFAFVTREAVVDKQY
GKIRIPAGTAVMAAVEYIHRDPRHWEEPDRFDPDRFLPENKSRINPMSMQAFGNGPRNCI
GMRFAHMELRYTFAHILRKYRLEKTENSEKDPPTIEMNPIILKIKNGVKVRAVPL
>Halo40802.61229
MMVFAYVDRWRKIRPAASPAFSTGKLKKMDGLIQDCAKVTCEHLKLAAEKKADIDIKQFY
GHYSLDVIARCAFGTRLDSHSDATNEFVKESRKAFAISITPKVLFAVLFPGVMRALRMKV
LNDESFIYFKKLCQRIIGERQGQQRQEDFLQLMMEAQKGGIAASTEVSSDMESQLFDIDS
VEKPDAAPCAKGLTEDEALAQCVLFFVAGQDTTSSTVSFAAYELAVNPDVQDKLRKEVDE
CVTAHGPEPSLDVISKLKYLHCVVSETLRLYPPGTRVERSATEDYVLGEKGIKVPKGCVV
IVPVYAMHRDPEFFPEPEAFKPERFSDSSVDSIRPYTYLPFGAGPRNCIGMRLALQAVKL
CLFHSVRNVRFVRTEKTKVPLKIAKGFGVLSVEDITIGIRSRKDQSS
>Halo40802.90861
MELGDAMGQLVAASCMLLLVHWYMKRKQHFCFFKDLGIPGPVPSIITGNMNELYKKTPVA
AYREWIEKYGKVVGYFNGYRPVLLVADLDLLKMIQVKDFQDFIDRSLLFQSKRPPSPHNK
SLIQLTGKRWKEVRSVLTPSFTTNKLKMLCPGVVSSVREFEATIDEYARSGEEFEIGNLY
QALTLDVICRSAMGIEYNIQKNPQHSLLVSSRLLFSSTFSWIAVLLTSFPELEFVLRYLN
DWRLSRTNNGVHPFKEVQEKCGHIVKQRQHDTTPPQKDLLQLMIEAKSSTVDIGIVTSDQ
LTAADDNEHELKQSTSLQSDGLLHPSKTVLDDDDITQNAFLVLVAGYETTSNTLTLVSHM
LVNYPDVQEKVRQELMAALGPDEEISYNTIQKLPYLNCVIQETMRLYPPIFAFVTREAVV
DKQYGKLRIPAGTAVMAATEYIHRDPNHWENPDTFDPERFLPERRRGHNPLAFQPFGAGP
RNCIGMRFAQMELRFTLAHILRKYRLEATPNSDKVRPPAAPPRVGCFTAIDTRGDHRGSV
ILRQ
>Halo40802.79228

MAESMFVPWLYDSRPFSSQFQSLLARVSPWLVVFALWLLLFRPLVQWVRIWLALRPIPGP
WDGIPFWYSVKAYWTVSKTTGLKDATVGFLTTVNQLTKEYQWKTFKVYLGPMPVVIVHTP
EAAETLLMSKENHRKPYVYSFLSTWLGPHNLVTATGDIWRFKRRLFTPAFHFRVLENYMG
HFNENGNVLIKILEKHVDEKPDEALATFPLMQHLTLDIIGRVSMGTVLGMQTDKGNPFGQ
NLNRLSFLITVRGFRPWTWIQQIYDMTYEGRVFKETLLDMEKFSLSVMQQRKEKLQEMEL
NGELTETDDASDAPIGKESIILDYLLKKHLEESSYTIDEVKKDIDTIIFGGNDTTTSAMS
WAFYLLGLNPEKQAKVHAELDEVFGEDRDRDVTKADVNRLKYLECCIKETLRLYPSIPLI
GRHLEQDLVIDGYRIPKGVNCFINLFSLHKNPQYFKDPESFIPERFLTEEISARHPYSYL
PFSGGPKNCIGQRFALLESKVIMAKLLLKFSVESTRPLDQVRVSYEVIVKARGGLRVWFR
RRPPVNA
>Halo40802.4034
MILLAAVAVTAVALALLFRWRKKKFSYFKERGIPGPEPNLLWGNLWEYHTLGLYKALDKW
CREYGDLYGFYNGDVPFLVIKDLDFLEYVYIKNFQNFTARGVTMRTDQMHPVVGQMVIHV
RGDKWRRMRSCVTSGFTSSKLKQMVPHLAESAEVFMDILGQYADTDTEVNLRLEFQALSM
DYIGQAAFGIETCFQRELNDIFFTTARRVLPGVMTGTAHMIAQSTTTLGNAMKPFYWFLG
QFASLTFKVFSKETTKMIELRKNNPELRKPDILQSLLDAEIEEEALSEAVKEGDVRGAAE
GIKKRVRALSPREVIMNATLLFMGGFETTSIAMCYLTFLLAKHQDIQDRIREEVKQVAGE
SGELDYDTITKKARYLCQVIEEGLRLYAPAVVATSRQAEEDFQWNGMKFKAGTCVMAPTH
QLHRDPRYWTDPDSFDPDRFSPENEGSIYKMAFQPFGVGPRQCVGFQMARMEIRYTLARL
LQCYRVELGESQKGVMRMDSYAMVSAPENGPWLKFYRL
>Halo39765.0
MGPIKRFSQPRCLIDEERYKKMGRIFGMYELGKPSLMVAEPDLVKQVLVKDFPVLTNRRE
LDLNDSILRNVLVLARGDLWRRIRPLTSPAFSTGKLRKMHVLIQNCANVTCKHLEEAAEN
KRDVDLKQFYGHYTLDMIARCAFGTKLDSHTDATNEFVTEAGKAFTRKASWGLVLGVIFK
GLVKIVRPQVIGAHSFVYFKNVLQEIMRNRQETGQRHDDFLQLMMDAQSGRLVSSTDNGV
LANSKLIDIGSESTSDATAPTKRMTEEEALAQCLLFFLGGQDTTSSTLALCVYFLAVNPD
TQEKLRREVDECIATHGTEPSLDVVFKLNYLNAVVLETLRLYPPLERLEREASEDYVLGQ
TGIKVPKGCVIGVPVYAMHRDPEYFPEPEKFNPDRFMGENEDSVRPYTYLPFGAGPRNCI
GMRLALQAVKLCLMHSVHKVRFVRIDKTQVPLKIINQVGLLHFEDISVGVRSR
>Halo40802.5364
MWDLVIAAVTLVVVYWLMKRREHFNFFKKLDIPGPPPNILFGNILEMHRKSAVVCYRKWI
DQYGDVVGYFNGYRPVLLVADLDLLRQIQIKDFQDFTDRALLFQADRPESPHDKSIIQLT
STRWKEVRSVLTPSFTTNKLKMMSPGVIDAVEKMVSKIDQKAESGAEFEAAEMYAALALD
VVSKTAMGIDYNLQHNPQHPFLICCRKMFGCAASILVALFSAFPSAVSMLKFINSRLLRY
QNNGKHPFIVVQEKCKQIVQQRQEDISLRKKDLLQLMIDTKQSRVDVNSITSDQLTAAED
NERELATGTISESATGKLPIFKKAPMDDDDITQNAFIIFVAGFETTGNTMALMTHFLSHY
PAVQERVREELFSVLEPDEAITYNTVQKLTYMHCVIQETMRLYPPVFAFVTREATVDKQY

GKVRIPAGTAVMAAVDYIHRDPRHWENPECFNPDRFLPENKSRINTMAMQSFGQGPRNCI
GMRFAIMELKYTFAHILRKYRIVKTANSDKNPPEIENSPLILKIKKGVFVKAVPL
>Halo40802.26660
MSSRFTQVDRQARAASLFEAPLAAHNKVTMSATAVLLLLIVVLLAVLLIWRWRHFSYFKR
LGIPGPKPNLIWGNLMEYHSTDLYKTVGKWIERYGDMFGFFNGDVPFVVTKDLDLIEQVY
VRNFQNFINRGLTMTSDQLHPFLGKSIIHVNAPQWKSVRSSVAYGFSATKLKLMLPYLEQ
DVNILVKLLEEHADTGKEVNMLPKFEQLAMDYIARGSFGIDERFQGNPDHPMIALAKAAC
CQLMTGPFHMIAQSTTSLAPLRKPMCWLSLAIGDFIFDTVTAQTTKIIEKRKKDPSLRKP
DILQNLLDAEYVETDAGPGGADGGKAGVVKSRALTTEEVATSAATLFIAGYETIVTSISY
LTFTIAKYPDVQEKVRREINDAVGEKGVLDYDTVMKKLTYLEQVMNETMRLYPPGLTFVT
RMAKRDFEFKGKKFQAGTCFMVPQYQIHRDPRYWPRALEFDPDRFAPENEGKLKKMAFAP
FGIGPRNCVALRMSVLKMKFTVAKLLQKYRIELGPSQMGEMPMASRAMVSTPARGPWIIL
HSLAKAA
>Halo43377.0
MIGKELILGSSPSHTSFQVFCGRMILGLLAYLFWTRVMRCCARWCRQWNGLRPVPGPVDL
IPFWRTVSVYWSWKGRVHFMSASAVFFQIICDMCERYRGKTFKTYLGMMPVVVLHTPDAV
QTILTSKTNMKKPIVYNFIVSWLGEHNILTATGASWRFKRRLMTPAFHFRVLSDYLSTFN
NNATALVERVEKLTAQQPEEPIRAVRLTQNCALDIMTKVLMGVDLGSQVDTTVTSFASQF
NMLMFLIGVRIFRPWMWSDAMYKRTMEGRLYQTAIEEMEKYTLGVLEKRKGKLQQLAAEL
QREPRDDVIGEEEENRHAVMVDTLLKAHLHDHNYRISEIKKDIDGLLFAGTDTTTSAVGW
ALYLIGLHPQVEAKIHQELDEIFAYDLDRDITLEDLKRMKYLDMCFKESLRLFPPVPIIG
RVLDEDVTIDGHLIPKGVTCFINMYSLHRNSEYFKDPEMYIPERFLSKQVRNRHPFSYVP
FSGGPKNCLGQRFAQMEAKTVMAKVLRKYTLESTRPINQLRITYEMILKARGGLRIWFRE
RTHVDRAARMADAEDKQTNHGL
>Halo40802.98726
MALFPGLLEWIVLAATSCLLLYLYVSRRRNHWTDQSITSEPYALIFGPTLKLLFGPMHEL
DHARYKKYGKLFGGFEAGNVILFVGKPELVKQILVKDFPSLPNRRPFKFYDPVLDNMMVA
APVEQWRKIRPAASPAFSTGKLRKMNSLIQDCAMVTAEHLKNAALKKEDIDIKQFFASYT
LGVIARCAFGTLLDSHSDQNNEFIAQSRRAFPTRVTPRLFLFFVFPAVTKLLRLNPFNTE
IFLYFKKICQNIIKSREDKQSRHEDFLQLMMNAQEGKLSATAENIAQRDNQLFNLGGELK
ADASFSSNKSLTEDEAMAQCVLFFAVGQNTTSTVISFTLYLLAIHPDLQERLHREVDECF
NTHGEHPSLDVVTKLNYLHCVVSEALRMYPPGARVERSAIDDYVLGDTGIKLKKGDLLAV
PVYAMHHDPQYFADPFTFNPERFNDENVGSIQPYTYLPFGAGPRNCIGMRFALQTVKLCV
LHVIHNVQLVRTEKTEVPLEFRNGFTALTAKNITLGIRPIV
>Halo40802.64483
MITTIADNFGLREGIFCAVFLALFLYTKHLGRYRHYWKNQNVVQEEFEFFWGPISTLLKK
SIEDIDMDRLKKYGKVVGTFEGPKPALIVADPDIVKLVLVKNFQLFPDRRHFDFNDPMLD

NMMSMVPAERWRKIRPAASPAFTTGKLRKMDSLIQDCARISTQHLLEVAERGEDVDVKEF
FGHYTLDVIARCAFGTKLDSHTDQANEFVAKARAGFNVKITLPTIMLFTIAGLFPVIMKL
LRLRIFNSGVFLFFKNVCLEIVEKRKLSPEKHQDFLQLMVDAQDCGLQQGSTEGPRDAED
KLYNLGTEQSIEKNASAKVLTEDEALAQCVTFFIGGQDTTSSVIAFTAYLLALHPDVQQR
LRDEVDSCFEKHGEEPSIDNVSKLKYLNCVISESLRLYPPAIRVERTACEDCVLGEAGLR
LMKGTVVTIPVYAMHHDPDNFPDPEKFDPERFSEENVASIKPYSYLPFGAGPRNCIGMRF
ALQVVKLCLVHAVRNFELVRTENTKVPLKMMRIFGLLLPEDITVGVKKRRP
>Halo40802.103351
MQDLGFWAWVATLVACVLYSVGQLLLRQRRKCFEVFKDTGIPGPPIKSLINGNSDEYWNP
TTFERIGRWLKEYGDVFGFYLGAAPIVVVKDLDIIKQVFTKDFSNFNARAHIMFIYELQP
FLSNNVAFARGRAWKEARSCVGQFFTPAKLKMVMPSLVDGQRQFIEVLGACADSGAELNI
TKYLERLTFDVISKMAFGIETDVQRKPENPIFQTALTVLPNIAKGLVYNAGQNLFPWPWL
LRRPLQLLSLCFANPLAAMTDKAKAVIEFRRQNPEVNRPDLAQILIDYVLGRGDAAGENS
AKVDPKVPIPNETMDMLASNSMAIFLGGYDTTRLAMACWFYLMGKHPDVQEKMREEVLKA
FAAEGEFLSVETLTNLTYTNQVISETLRLYPPVVTLTGRAAEEDRRYGKYLIKKGTSVMV
PVYQLHRDPLYWEDPEKFDPDRFSTEKKHLINPYVYQPFGLGPRLCIGQRLALLELASVF
TQVLRHFRITLGPSQKPDLELHTYSVLAAPKDGVWLQLHKLPARDK
>Halo40802.54191
MSSGAVAAGNWNFFTPSRFDAVLLPSLPWISVLLSCALIAMHCRRWFNVWSLLRSMPGPG
PDWLPPWFLLSIYWKYRKELSTSPTSVVYKVISELSKKYDGKTFKVFVGITPVVILHTPE
AVEVLLSTKENTGKPNMYRFLKSWLGPKNLLTSKGDQWRKKAKLFKSAFNKEHMQNCVEV
FNRNGAILEERIASMAAESPDQPISCYKNIQMCVLDIIGRATLGIELGLQNGKRPEYARW
FNCLTFLITIRYFRPWLWIQGVYNMTREGKVWKNTVRNIGNLHLAVIQRKKAELLKKASG
QDYSDLDETDDGLSFRVAVDVGVENHLSCPSYTLRELETDITSIMFAAADSTSAAMSWTL
YLLGLHQDKQAKLQKELDDAFGRGVEHDFTMNDLKELPYLECCIKESFRLCPPFPLIGRE
LDEDLEFDGYIVPAGTTCMINIHSLHRNKDHYTDPEEYIPERFLSENSRNMHPFSFIPFS
GGVRVCLGQKFVVAEAKILLAKLLSKYTVEATQPLEEVDAAYEVVLKARGGLNVWFRKRA
ESE
>Halo35200.0
MYYMPVHFIWDELYRKYGPVVGTFTTSTPTLMVADPALLSDIMVKNFASFPNTQFIRQVG
DPVLDNMLVSLMDNEWRATRSVVSPVFSTSKIKQMVATVNECAKDTLRNFADAAAEGKPC
NVKLIFGAFGLDVIARTTFSFSLDSHRDVNNPFVLQAKRFFSAEHKWRAFVCFQFPRLST
LLGVRIFCPEAVQYFSGVMTNLLKRRAENVDDNVRPDFVRLLLDAECKDEDGCQTRGSKE
TARRVLSKEQVLAQAVLFFVAGYDTTTNAMSMTVYHLARNKHVQSRLIEEIEDTLAKHSA
ITFEVLMGLEYLDAVIMEVLRINPPVHMTYRTCREGTTVGNIPVQEGTLVRIPIFSIHHD
EKFFPNPEVFDPERFLGENKKDIVPFSYLPFGEGPRQCIGMRFALLTVKLCLFHVLSRFS
FELCPETAIPPKYHPSILVLTPYEIKLKIIDRQRSPEEK

>Halo40802.17827
MVVLALLGSVAALLATWLLLWVRGRRKAHGFLRAFGIDGPPTDLLWGNWNQLKEDRIQVM
EDWIAKYGKVFAFYKGTVPYVVISDVDLIKECFIKESAVFYDRPPAALPVKPYTDMLLFL
RGPDWKHVRTVLNPSFTAAKMKLMTPIISRCVEDFMSIMEQKADKEEAIDVMEVAQGLSL
DVIANCALAWQLDSQKKPDNPLAQLLRGILYEAETAFTTAFVAFPILGQCASWMYGATLH
SKRTWEIIDNVRRVVEVRRRSREGASAAGGGNTRRMDILQLLLDAQNAAKPSPAAPHQAC
DPPAEELSPIDDDTLLANSLLFLVAGFETTASTLSFIMHLLAQHPHEQEKVHEELMRRYP
SDEELDYGDLQDLQRLDMFMKEALRLYPPVVMLVSRHCRADTTILGQFFPAGCEILAPVW
HVHHDPQLWPDPFCFNPERFAPEVLKDHHPGAYMPFGIGPKSCIGNRFALLELKAALCKL
LRKFQVLPCARVEDPMKLVVKTVLAGPETPIQVKLRYRVAASHVS
>Halo40802.61717
MWAALIVSLLVVAASTALYWLFSRKWKLGLFQRHGIPGPKPDLFWGNFMQLREDRIQVME
RWIAEHGKVFGFYMGEEPFMVVSDPEMVKQCLVKEFPTFHDRPPFALTVEPFASCLLCLK
GAEWKRVRSVLNPTFSSVKIKQMSAIVRGCVDTMMEVLEEKCRNGQPVDMLKVAQGFSLD
VITKCAFAWQVDCQRNPNDPLLLGVRKLFEEAEQPAVQNTIRFPVLRYLFTALYRLSDYH
KVMQQMVNKLRQVIELHRRDRRALATDMLQLMLEAQEGEDVATAESARGKDVRLIEDRHV
ISNAFIFMAAGFETTATSLGFIMHLLATHPEEQDSLHAEIGAAFGDVGEELSYERVHQLK
RLDMFVQEALRIYPPVVLFVTRKCEKDTTVMGQFFPKGVSIMAPTWHLHHDPAVWPDPFV
FRPERFDQGGNGGDGAQPHHPGTYLPFGLGPRICIGKRFALLEIKMAVCRILKQYRVVRC
HQTQEPVKLIVQSIIINPEGGVFVGLERR
>Halo40802.90193
MRRIPRTLLAAVPRASGRPNSSLATAAAEKPCPVEQHDVCPVHKAETVLENSRSRTVGAV
STARPFDEIPGPKPLPLVGNIWRYLPFIGELDLTRMHRNAQKLLDQYGPLVREVVVGDRV
VVHVFDPRDMEHVFRNESRFPARLSHRALLKYRRERPHVYGSGGLFPSNGEEWSRLRHIF
QKPLMQQGAMSAYMDVLQEVTWDVANLVRQTRDSRTLEMEDFLKELYRWALECTGVLALN
TRLGCLQGGLSSDSEQQRLVEAASETHRIIMVTENGLPFWKVWNTPAYRKLVDSQDFMAS
IVNKYLEGAIAAMRQDTAGERTVLEKFVTTPGIDIKDVFTMILDMFLAGIDTTAYSTTFI
LYYLATNRHCQDRLAQELRTLLPTQDSKLSLEQLQGAAYLRACIRESLRLSPIAIGVGRV
LPDDIVLSGYNVPAGTVLIMHNQVACRQASSYPEPDTYRPERWLKEERPDAGRAHPFTLL
PFGYGPRMCIGKRFAETVMCLLVARVVRNFVLEYKHEKLDCFTRLINVPDKPLKMTFIDR
DS
>Halo40802.3745
MAGGFVVLLAVTSLLAAFATWVVRRRHKQGLLKRYGFPGPKPNLFFGNWLELKKDRIKVM
EGWARRYGKVYGFYEGEVPKVVIGDMDVIKECFVKQAHAFTDRPPMVISVEPMQSCLIGL
KGDEWKNVRSTLNPSFTSAKMKLMLHTIDQCADTAIEIVNERVSTGGHADINVSKLCQGI
SLDVITKCALGWQSDCQRNTEDPTVKTILKILLDSGNCISDVCVLVPSLGALVSRIFPFL
TYGKLFSRIQDNLRQLVKSRENTSPAVPDIVQLLLEARRKQTNGNDPEKVKTPVVEGPFN

AKLNTGFMTDTHIVSNCFLFLVSGFESTSATIAFALYELARHPEEQRRLHSELMTSFPDN
ETLTYEDLKVLKRFNAVIKESLRLYPPLVMVTSRTCSKDIPLATGHVIPSNAHVLLPTWN
VLHDPDLWSDPYSFEPNRFSERLQGIQLHASRVAFGTGPRECIGKRLAELELKAVLSKLV
RKFEFSVCSETQVPLKIKVPLVNVFPERDIWLCATKRPT
>Halo40802.20342
MFQNIVISVAISLLAWFYLQRKRRLSFFKDRGIPGPPPSFLSGNLSELIEKGAAQAFKEW
MDQYGNFLGFFNGGYPTIIVKDPQLIKKIQIKDFGNFHSRGVTSVFSQSHPTNKNSLTNA
SGQRWKEMRSLLSPAFTTRNMKKMFNLMEDCTKEFLGILDGLRSRKEAFEARELFQRLTA
DVILRSAFGLKSNVQQKRETTSIGEALFKNSQKHFQQFRHAWRTYLIACFPEFSAVWCIV
LHFLARFRKAATDNILSDLMAIIKFRRENREDERTDLLQLMLNAEVEEGATVNVHSLTAN
YEADTALEENNMTKITSIKNKRILTNEEILANGLLFFIAGFETTGGTLSFMSYLLAKHQS
IQDRLREEVLEVLEREGSFTYNSVFGMKYLDQVISETLRYYSPVVGFTTRTSANAYVHNN
FTIPAGITILVPGHHMGRDPDFWEDPDKFDPDRFSAENKHQIDPMVYQPFGQGPRNCIGL
RFAQLEAKLTMAKILAKYKIVLDDRHLKEKDLEIGSAFVFAYPQHGIWLKLEELP
>Halo40802.58670
MKQGARALARSVASETSVSLGTQPPGSGAAKAQELRRAEVYPKPFHKIPGPKPSLPFIGT
SWQYSRWGRYSLYQLHDASADKYHRYGDLMKEEYQWRKPIVHAFNPEDFQVIFRNQGRCP
VRPPNEFVCKYRTEHPLKYNSVGLSNALGPEWHTLRMALAPALLQMKNVAGLACWQREIC
EDFAEYVRWVRDPDTLVISNIQDSLSRLALESIFMLCLDTRLGCLKPSKSEPGEASTVIK
AARQLFSAYQELYYGLPLWKYMNTPPYKKYTEAENVLYKITLGYIQHYAKQKLEETTNLK
DKSLLQALLSLDRLSEMDIHLTVMDFIAGGVFTTSIALCFLLHHLACNPDVQQRLYTELK
SGSKEVSSCSYLRACIKESFRLSPTVPGVMRILPEDVVLSGYSVPAGVPVFANSLVTCRL
AKYFPQPERFRPERWLGEARSHIHPFSMLPFGHGARMCVGRRFAELELMTAAAKMVENFI
IEPCTQHINTSYVFVVVPSHPVALRFRDRK
>Halo40802.85839
MITATAARRIPGKSAVFQALPISVSASCHVDSLSFAKPFRDVPKIPALPLVGSSWIYFPI
IGRYNIRNSSDAALDMYRRYGPIVAEKLPGRGVLVHLFSADDIRALYQEEGRTPYRVGAL
PFKLYHTGRPDYFANAGILNAQGEEWRRIRAMAQPCTIRPRTIQAYAEAMGQIADDAVSL
IDSYRDHSGDVPDCHAIMKRWALESVMLVSLDKRLGLLEEPLRPDSEAARIMHGVVSLFS
GMDKLATRFPYYRYFSTPTIRNFERTGDYLVPRLFHIIRKAAETTRDKEDQNCTILSHLH
NVEKVDFKDMFTFLHDFVVGGTDTTAGAATFSLYRLAINPDAQEKARQEILSSSCKDPDS
AASENHNHLPYLKACIKEALRFHPIIPGINRKVSHDVVMSGYKIPANTVMRSEPFVAGRL
EENFTRASEFLPERWLRRSEQHQNEVSGTTEAWTLHPFASLPFSIGPRKCIGRRIAEMEL
CTLLAKVLRKFKVENPHGDIGFNTEFSGKPAKPARFRFVELEN
>Halo39665.0
MRRVTTPSLLKRNAKFAARAYSQPAVASASDEAVKPAASSTKPRPFSDVPRVPSLPLVGS
SWMYWRLVGKYHPDRRHLAAVDMYKKYGPVVAEKLPGRYSLVHLFNADDFRTLYQEEGKT

PFRMGATAFKKYRETRPEYYANIGILNMQGQEWYNVRSKTQPYTLRPRTIMSYVPGMDAI
AQDALQLIEETRDEKGEVEDAYPILYRWALESVTLASADTRVGCLDNPLHPSSDGAAFLE
DMNNVFNCLQIFGYRFPYFRYFRTPTWRKFEKAMDAFTHRLFKHIQEAAERLQTTEKDQE
YTILEHLLVEKKLAFGEILAFMSDFIMGGADTTSSSATFCLYNLAKNPEAQERARQEVLS
VVGENCTAVESRHLNNLPYLKACLKESLRFNPVLSGVFRKLDHDVVMSGYTIPAGTPVFT
ENYVASQLEENFTNAAAFLPERWVKTEEQRDNWVLHPFASLPFSFGPRMCLGRRMAELEV
WILLVKLLVKYRIEYHYEDIGFLGKLANAPDKPARFRFIEIQN
>Halo40802.86294
MSTVLVAMLVSPLWDWRWLTTAVVFALTYLVGRFYHRVSKYPRGPFPLPLVGNLLALRNS
KDLPYRAIEWSREYGDVFTLWMAHKPMVVLSSHAVISEAFLDRRHEFAGRFPTKMGALQT
RGDHDIIFEDYNPRWKALRKVALLAVRKYAVSESLQTLCTHVVDTYVDSLKPGPQIFDSR
KPFLSIIFKIIGMSVYGTRLDEGSIDMAQLEDLDRRFYGLAPNGLPSDIAPWLGLLYRGR
ERAIEALFREGHGILGSLFTRAEESYVPGKTENFTHAMLAAREEAIREEKGDAEYLTKGN
MVQVVVDVFGAAKDTSAGELQWMFLRMAKEPGIQAKIEQEIEENIGNRPPVYADREKLPF
TVACLLETLRCHPVAPLGLPHNTSTDTEVGKFAIPKDTGIMYNIFGVNRDPKLWDEPEEF
RPERFLDPVTGKLRQDTGPLITFGLGPRTCPGQKLAHVDMFYVLVRLIQRLSVRAPGKTS
EVDLRGIGSSLFLLPAQQNIVLTRKN
>Halo40802.47786
MSVVSLAKLVSPMWDWRWITTAVVFALTYFVGRFYHRVSKYPKGPFPLPLVGNLLTLRKV
RGLHHKATEWTKVYGDVFTLWMSHKPMVVLNGYTAIREAFLDRRHEFAGRFPTKMGELQT
QGNHDILFEDYNPRWKALRKVALLAVRKYAVSESLEKLCTNVVDDFVDNLKEGPQVLDSR
KPFFTLLYNIIGISVYGTRLGEGSDDIRRLEELDRLFYEVSPDGFPSDIAPWLAILYWSR
EHKVKLLFEEFLEILKRLFNKAEENYVPGKTENFTHAMLAAREQAIAEEKGDSEYLTKGN
MIQVVVNIFGAATDTSAGELQWLFLRMARDPSIQAKVQQEIEENIGSRPPVYADREKLPF
TVACLLETLRYHPIVPLGLPHNTTTDTKVGGWDIPKNTGVLYNIHGVNHDPKVWDKPQEF
RPERFLDATTGKLCKEIGPLMTFGLGPRTCPGEKLGHVDMFYVLVRLMQRISCRAPGKAQ
DVNITGVGSSLFSMPAEQKIVLTRRN
>Halo40802.78463
MHKKAVAWSKVYGDPFTLWMGSQPMLVLNSHDIIKEGFIEKRHQFAGRYHTNMGDAQRHN
DCDVIFEDYNATWKALRKVAVTAVRKYAASESLEKLSVEVVDAYVDSLGDQPKTVDSRDP
LLFIIVNIIGMSAFSKKFDPNSTDLARIKAINHTFAELAPNGFPSDISPFLGVLYRAREK
KLEALFGDIRCILNELYEGAKESYAPGNIENFTHAVMSAREEAIKQDKSDADFLTEGNMV
QILIDLFNAGTDTSMGELQWLLLKLSREPRIQEKIQKEIDDQIGQSPPTMQDRDNLPYTV
ACIMETLRFYPLAPMGLPHKASCDSYLGGVPIPKDTRLLYNIYAVNHDPELWTDAEAFRP
ERFLEPATGKLLAKEKLPPLLSFGLGPRACPGEKLALADMFYVLVRLIQRVIVAAPEGMI
GGEVRPQQSSFFLRPGNYNIVLSKRH
>Halo40802.75482

MLGAMLWKCLATGLVAGFMALLLQYLWDLLKRNLRRDLPPGPYGLPLVGYLPFMPKGGHR
GIEELKNKYGPVFGVHLGSRYVVFLCDFDSTKEALSQDCLLNRPEEFPFMVNEDSQGLMV
LNGPLWKEQRRFSLRLFKNLGIATQAMENHIHEELSYLLREVEARKGGRVVPTDVLTPST
SNVISALVFGRRFEYDDPERIYLDKLIELIPALSAQVSAINFFPWFRKLLVFFRFGTCEQ
LRDALVRRENFADSKINSHQSTYQDGLVRDYIDGFLSEMKQQGEGNQTFTRNLLKGNVAS
FFGAGSETVRSAIEWLLLHSATKPEMQRRIHAEIDDVLGSGRGAVSWGDRNRMPYTQAFM
WEVMRCKPVNPLSVMRHASKDVRVGKYVIPSGSIVIASIWSIFYDASFWKDPEVFRPERF
LTDGGTRAQKPERFIPFSYGKRSCPGEVIANMEVFIYFTTILQHFVVEPPPDGPGLVFDE
VLGLSLRPKPQEMLFRPREVRT
>Halo40802.84731
MFLGLLQGSLMDVPAWIYSWSAATSLLAFILTVLLALNALTPKSRKRERKRLPPGPMGVP
ILGYIPFVRKPFHVEFKELSEKYGPIIRLRLGCKEVVVLNDLDSVREGLTNPDVLFRSND
FVFRYLGIKGIVSMNGEPWQENRRYCFHVLRNLGYGKRTMDKHIQEEVQYFIDLLRSENG
KPTQVAQPLAASVANNISALVFGQRYDKDHPTGRSIESLLTMFLRNGNFFSLLDFLPALR
LLSVYIPNTRLHIMYRVFKEMRQLVRNEVKEREGNMEHYMDRDFIDGYLRKIKENQGNDH
HYTLQGLEGNAINFYGAATNTVRSAILWNLYIAAADPDGHQARVQLEVDTVIGQQRAPEW
EDRHRMPFTMASILEALRWKTTAPISIQRTAGRDTVIGGYHVPAGTFVVPNFWALHNDPE
HWPNPSQYDPTRFLNADGTELGRRPEAFVSFSLGRRACPGESLALMELFLYVSTVLQNFR
VLPEEGKTLSLDAVNALVLVVDDTQRLRFIPR
>Halo48359.0
MELAKVYGPVLRFPMGVKNVVVLNDYESVKEILPRKEMLFRSENNVVSQTEYQGIGTLNG
DHWKQNRNFCLHVLRDLGFGKKSMEEHIMNEAQCLLEKIAESKGAPEDLDKYLTPSVSNN
ITALVFGRRFPFEDHRRKFLDDRSRRLTNLFQSGSRFTFFPSWVFWITNFLPNATSHLVK
QIFDELAGFISEEIEQHEETLDEASNRDFIDGYLKKMKEHEDDPNAKFKKVNLTGNVMAF
FGGGTGTIKSTVYWHMLNCADKLDTVQRRLQEEIDEVVGKERAPRWDDQSRMPYTMATIW
EMYRWRPIAVFAIPREAAEDSVYKDYVIQKGTVVIPNIGAVHMDPAHWEHPEEFMPERFL
NGDGSGLKPKPQQLIAFSVGKRMCPAETLATVEVFIYLTTMLQKFTVMPPDGCRINLQSV
TPAVNNPQLQELRFISR
>Halo40802.8567
MTWAVVFFTSGVAIMAGLLVLLLGKKALRGGIPPGARHPPMPPKNSVWGHVEVLRNGFHQ
KEALEWSKAYGPVFRLKLNFYNIVILNSFEAIKRFLNTKELLNRSHCFLPSRDYYTGVGS
LNGIPWTANRKFCLHMLRNVGFAKAGVEDNMMEEFRRLSEKIASTKGEPIQVRQFLLASI
SNNIASFFYGAWHPDHSTVLELQRIMRQLSDVITTGPMFQFTPTVLRRLQYHLHFTRNSR
LNAAMVDLEEFSKRQIEVEKNTGRQDEIIDFIGGYVEKIEHEKQHPDARFTYRYLVGTAT
GLLIGGTFTTAASVQMHLVNFASSPENIQSRVQKEIDEVIGQDRQPTWEDRKRMPFTMAC
MWELERWKTTSVFAAPRECSADVVIDGFFIPKGTVVLSNLWAAHHDPVIWKEPQKFIPGR
FLNEDGSILSHKPEGLIPFSTGRRSCPGETFASMEIFLMVTYLLQKFQVLPSNPIHLDLE

SYEVLPNQLQQIKLRFLPRNRTDN

>Halo40802.108319

MSWAWVASLSAHTLDWRVIFVTIAVLFLTLRRKKQQIPDGKVLPPGPKGWPLIGHLPNRA

KVFDYRKCMELAKEYGSVFSIHMGIKNVIILNDLESIRDILNRKEMLYRSENALVNQTRF

KGISTLNGEAWRENRTFCLQVLRDLGYGKKPMEEHMREEVEELIEKITEAKGAPMILDSL

LQPSVSNNITALVFGRRFPYEHPTRKFLDDRNQKLSQEFESGTHYVFFPQLIFKISNLLP

RASSRRVKEIFEDMGKFIRKEMESHMSTLDETSNRDFIDGYLRKMKDHQYNPDSKFREVN

LTGNAMAFFGAGTHTVKAMAIWNLFICANNVETIQRKIQEEIDRVTGGERAPRWEDQSKM

PFTVATLWEMYRWRAISPLSLPREASEDIVYKDYFIPKGSIVIPNIGAIHMSTSHWESPQ

KFMPERFLRDDGTGLKPKPDCLIPFSIGKRMCPGEILATVEVFVYVTALLQKFTVLPQEG

SRVSLESTSTALNFPRPQKLRFLRR

>Halo35494.0

MEWAVPAVAAGAALVLAVVFLVRRHLSEVERQRSIPEGTKIPPGPPTLPVVGHLPTVLAG

FAPEKAFGWARKYGPVVRVKAGSSETYILNDLESIRMFLSHQNLLYRSLDWEMNLGVNIG

FSGFGGHAWDENRNFSLKLLRELGFAKSVMKNTLSEGCQLLLDRIAEERGNPLDISELLM

DSLSNNVGMFVFGRRFPHGQPERKGLSQALRDQFRASRSGDVLGFGPDFVRSLAKRLPFT

RRGLLFKVLKWLEDFLSNQLAWHMKTLGDDENRDYMDAYLRQREGRENDETTTYSLPCIV

GNAMGLMIAGTNTATAVLQRHLLLQAAYPDTVQARIQDEIDAVIGCERQPAWEDRHAMPY

TMATILECHRWHTLLEMGVPRRAKQDTIIGGYFVPKDTTVIANVWAVHHDPRSWHQPNKF

DPTRFLKEDGSLSWEKAEKIIPFSVGRRMCPGEIFASVEIFIYLTGFLQTFRVLPQEGCV

VDINVEDRVLLKCNQQKLRFVPRFSLLPHRREDR

>Halo46584.0

MLRRLFAAISTALLLTTCMYLWGKYKPRRDIVFAVLSAVCAIGPLLCWSRMRRLPPGTRL

PPMPPSQSIRGHLELTRKDFFSNKAMEWAKAYGPIFRLRVNLKYVVVLNDIETIKSFYSK

DEVLNRSRCFLLNFENYPGLAYLNGETWAANKRFCMSTLKNIGFATASGEERIRDEVSLF

AQRLGETSGEPVLLHQPLIACITNNVLDFLLGRHLRNKDAFRQQLHLVVMKLAMSLVADG

SLDFMPSSARKILGHLPFSGRPKPLVALDEMEDLILNQLKEYDTSSEADSNDDLVHLYLK

KIEETKYNPDSPFQYKFLVGHMGSFIVGGTLSPWDSMQQTFALLAMHKDTLQKKVQREID

DVVGLHRCPTWEDRKSMPFTLAWLWEVERWKAAPISVPRDAKDDVIVGDLFVPKGTMIVL

NKWAAHKDPRYWKEPHKFDPSRFLNDDGSFMEEKPESLVPFSLGKRSCPGEKFALMEMFL

MMTFVLQKYIVMPDKPLSQYEENPDIKLSNAGHVKLRFLPRPTSTT

>Halo40802.42363

MRTTASAFGFVGWTSQQTNFLKEGPLVKTGTFSSRAMISLCQETALIHLSSSDGSTFLLV

FSVLFSEQEESQYLAERLAATQGAPIFVQEYLIPSVSNNITALVFGSRYSVDDPRRKFLD

ERLARVLKALAAGQFFTFMPSWMNNVAAWLPLLRSTGLKDTIAEVVGFIKQQIQEHRDTL

EEHSNRDFIDGYLKKIDENKSSPTSNFTERFLVGNVGNFFGAGSNTVQVSMQWHLLNCAH

KPDSVQRSIQAEIDHVVGRERLPCWEDHMRMPFTVASIWEMYRWRSVATLGGPREAAEDV

TFKDYFIPKGTVVMSHIEAVHKDPKHWEDPEQFKPSRFLNDGGLVPKPEQLIPFSIGRRM
CPGETLATMEIFLYLTTLLQKFSVFPEEGRTIDIVSMATGLSVPKQQKLRFVIRD
>Halo40802.40762
MNSSVGSMSWGVIFATCGLALLLRLLIFVLDKKALHRRIPPDARLPPMPPKTSVWGHVEV
LRSGFHRTAALEWSKAYGPVFRLKLNFYNIVILNSFETIKKFLNTKELLNRSRCFLTARD
YYTGVGSLNGIPWSAHRKFCFSTLRKLGFAKTDMEDHMMEEFRRLSEKIAQTNGEPVEVG
QLLLPTILNNIAAFFYGSWPPDHPTRFELHRIMMHLCDVVNTGPMFHFTPPVLRRLQSGL
HFGRNSLVNDAMLELEEFSKTQVEVHTNASCKDGMTDFIRGYAQKIEHEKHDPDPLFTYR
YLVGTVNGFLIGGTFTTAITMRMHLVNFARSPDNFQARVQKEIDEVIGQNRGPTWADRKR
MPFTMACVWELDRWNTAGFLGTARECSADVVIDGYFIPKGTVVLPNIWAVHNDPSLWKEP
HKFNPGRFLNEDGSLLEHKPEFLIPFSIGRRSCPAETFASIEIFLMVTYLLQKFTVLRSN
HVDLDLDSYDVFPQDIQDMKLRFVPRQQT
>Halo40802.47195
MVVEQFLAGALVVFAALLVFVQQFRKKRAHKKLPPGPTSFPIIGSSHIVAKYRNAWDAFS
DLRQHYGDVYAISLGSRRCLVVSSVEALREVLVTKASDFADRPDSLRYHAIFKDDRNLSI
ALCDWSSKQRTRRELCYPSMHPKHGSTEQSRLSASIEDELAYLAAELSRSRGTPLKPRQL
LLVATANIFYTFLCSERFSPDDQKFLHIVDLYNEVFHQLFQGFAIDFMPWLKVLQSKQLG
ILRAKSTEIYHFTVNIMETREKALATQGEPLNLTDMLLLSLKDPDAERRLSRVEVAVVIE
DLIGGHSVIANLWLWCLYILSSYPEVQAKIRAEAYSVLSSRKDTGLALSDRPHLSFTEAT
LYEVIRVVNSPIIPHVCSNATTVQGYHVPRGTVVMFNTNDINYSAYLWQKPWDFKPERFL
GEDGSVLKPGHFFPFGTGKRSCMGDGLVRTILVLGLASLMARFELSLGPSQKPAHFASFR
SKVIFDQDPEIVFTDIATQRST
>Halo40802.108315
MGCAGMAIYIIGLGATILGSALWWFLRSRSQPRKHQPPGPTGIPFLGNFEVTKRDFFYKK
AMEWVKLYGPVLRFRTAAVSTVVVNDYDLIKETFSKPELLHRPSAWLLKDTTEGLSVLGG
QEWKANRRFVAEVFTELGYGKQAMWDQVQEEALHLVDMISKSNGAPILPRDYLARSACNN
AAFFLFGRRYDLDDPRRKDIDEHLEGFNLGNAAFAVDYRPAGLKALARRLQPRSANVLTK
DMARNFEAMSKRQVDLALQTDKLQRNRGLVDVYDEKLQEHGDPTHPVFTEARLVGNVGDY
MLGATAVVALFLHSHVLNFAARADSLQAAVQREIDSVVGRDRLPNWHDHVRMPLTMATIW
EMYRWKACTPFGVPRGAAEDTTIGGYHVPKGSVVLANFWAVHMSPKLWKDPEIFDPSRFL
RPDGSATSTRPEHIITFSIGKRMCPGESLSTAEIFLDLTMLLQKFRILPEEGATIHIESH
QPLLEFANTRLRFVPRHD
>Halo40802.91253
MAFTASKLRPMVTGMSEAVDRFLNLLESRCREAADGEADVFPLLGALAFDMVAETACGLT
LDVQHKPNDEYYATASHSMMLNVVESVYQQVGQFFSGLVPLACLLEATFAQEPLTTLTHK
AKPIVALRAKDSSLSRPDVIQSLLEARVPKELLGQHDLRARSDDKGNLFMHYRDVASNTA
TVLVAGFETVSASTASCIFCLAKHPDVQERVRREVHAAYEKHGSFSYDAIKDLPYTTQTI

FETLRLYSPVVAFTSRRAACEYRYKDLTMPKGLNVMSCTHQIHMDPSKWDQPDKFDPERF
SPAQRTSRNPLAFQPYGIGPRNCVGMKLAQLEMTLIIAKLLHRYKLHLSTRHASGELDRK
TQSIIASPQNGVWVRIERMSYILA
>Halo40802.16546
MNIYSEGDGWKTKARLFKGAFSSELREMSMDILNKNSKIFIETIEADAKDSQPRAKDCYK
MAHACVMDNVARVTLGEDLELQKGKNQHYLEGFNTMTWLTLARVLRPWAWFQPLYFLTSH
GKIWRDTAQKIDAVHLAAIQKRVLAVKKQLSGLNKSSKIGEDDTPFPYGVDSYLKTHIND
PSYTIQDVLEDTLATSFGAADTSTASISWTIYFLGLNPDKQAKLQKELDDAFGPGVEREY
TSTDLEKLPYLDSCFKEALRLCPAVPIFGRELKEDLVLDGHTVPAGTTCLVNAYSLHRNR
KLYTDPEKYLPERFLEENSEGKHPFSFLAFSGGIRPCLGKRFAYAQAKVLLATVFSKYSV
ESTLPFQDLSLGFEITLRAKDGLKVKFRKRNL
>Halo40802.2039
MMTPVGGIVIAVILVLVITVLDVVMKSYKRLRLFEELGIPGPKPQFFWGNMKQLRKNRNE
VLTKWKTKYGKMFGTYQGHEPFLVITDPDMVHECFVKQAAIFLDRPNALVDAEPFRSSLF
QIKGQEWKFVRRFLEHAFSSNNIRELSKTADVCTTRFVESLTKAAGIRGSVDVTDFAMGY
AVDFLTKCLLSWDMDGQEYHGNELVECLTALSKELDGAAVEIAFTMPVMRCILSWFYPFT
KHARMFNELIERVRNTVESRSSRKSPRKKDVLQVILDAQSEDCNHPSKAGCKHDNLFHDQ
YMFSNIVIFMFAGFDGMSATLSFLLYLLAKHPREQEAILREADEYFPNKAGQLLSFDELR
QLKRLDMVTKEGLRIYPPVPVTLVRQCSEDTTICGQFVPAGVNVAACPWLIHRDADIWPN
PDQFLPERFAESSTAVKRGAFLPLGLGPRKCLGKELGLLAVKMALFGLLQSCILSLKDKT
AGPPRAVARALTLIPEGGINVTLRPRTQK
>Halo40802.41450
MLETYYWAWLTSLVACGLYGLYRWVLRERRKWFDAFKDTGIPGPPIDSLISGNSDAFFKP
TQLESISRWLKQYGDVFGFFLGAVPVVVIKNLDMIKDILNKDFSNFIARGHMKHMCELQP
LMEDSIGFGRGRAWKEARACMQQFFTTSKLKAVMPSLLHGQRQFIEVLGASADSGAEVDI
TKLCERFTFDVISKTAFGIDTEVQKNQDNPLFQAAIVVFPNSMNGFAYHMCQNLYDWPRL
LRFLVKVLSMFVSDPAAEMRTKAKAVIEFRRQNPQVNLPDMAQILIDDALSRRDAGRKMD
RSKTDSRAPLPPAKLEKLAGNGMCIFMAGYDTTRLALTYWFYLMGKHPDVQEKMRKEALE
AYKLEGDPLSVETLTNLSYTHQVISETLRMYPPVVTFTTRCAEEDYRCGSYLIKKGTSVL
IPVYQLHHDPLLWVDPEKFDPDRFSPENKHLVNPIAYQPFGLGPRMCIGSRLGLLELASV
TTQVLRHFRITLGPSQQPDLELNTYALLAVPKDDVWIKLHRLNGTH
>Halo32110.0
MDVFHKNSDAFLAQIEHALKISPDKPFNCFRGLQKCFVDIMARVCMGVELNTQQDERNFF
GNCFNRLSYLTAVRGVRPWLWAQQVYDLTTEGKIFKHTVHEMQKFSYAVLSERKRQLLQN
SPASLMDKGAKIPTTMPESSSLFLDSLLSYNIKDPTYSFQDVKNDIDSIIFAGTDSTASG
VSWTLYLLGLHPCKLAKLQEELDRVLGSDSDRTIAMQDLLQLHYLECCIKESLRLYPPFP
LFGRMLEQDTVIDGYSLPEGVTCFVNLYSLHRDPRHFPEPETFLPERFLSDDFGLRHPYS

YIPFSAGPKSCLGQRFFMLEAKLLLAKVLSKFSVVSTRPVDQLKISYEVVLKSRGKLLVW
FHERNGPVS
>Halo40802.88768
MPYVVNCQRKFIEILGTLADSGAEVDILTYCGRLTFDVISKTAYGIETDVQRNLDHPLFQ
APHKVVPNFMDGFLYSMGQNLFHWPWLLKFLMQLFTLCGGNPISPMHEKAKAVIEFRRKN
PQVNLPDMPQILLDDALGGREATSGNCSEAKTRAPLSPATMDKLAGNCRNIFLGGYDSTR
LTLTYWFYLMGKHPDIQERMRKEALEAFESEGEYLSVETLTHLSYTNQVISETLRLYPPI
ISMTARAAEEDRRYGRYLIRKGTSVMVPTYQLHRDPLYWDEPEKFDPDRFSPENRHLINP
LAYQPFGLGPRGCIGQRFGLLELASVTTQVLRHFRITLGPSQKSHLELRTLAVTAVPKDS
VWIQVHRLNGAK
>Halo44927.0
MKEQSPTVKDLRLSKQLGRVYGTFEGDLPALFVTDPELIKEIFVKEFSSISSTSVGEYFH
PLLDNMMSLCPAEMWRRIRPLLLPAFATGNLRKMNTDMQYCARQIAERLVDFSENGQHVD
AVSLFGQYAAQVIARCGLGVEINSSDRLIEDLVPIAFAEKCPPMRQAILNIFPGVANFLG
IGPIVHQLARDGENVAKKLLQLPRKPVCEGTELLQPALEILNMSKGSVRHPSDGTSSDHR
KKRCEQDFTEDEMMAQCVVYLLAGTETPATPLAVAASYLAKNPDIQLKLQNEVDQCFAEI
DQEEPSFDDISKLKYLHSVISEAMRIKPPIPRLDRCVARDVTLRKSGIKLRKGMPVFVPV
YALHHDPDFFPEPSTFKPERFLDDDTEAARRYTFLPFGTGPRACIAIRFATHAMKVCLLQ
AIRHVEFVDIERTTAQISCEAPSDDQKAKPGKLFVRVKKREKPRSI
>Halo40802.56980
MVAELVTTLLWFLLAFLVTAVVLWRRRHFSYFKRLGIPGPEPSLLWGNIREYHETDHYKV
IGRWLEIYGDTFGFYDGDVPFIVTKDLNFLEYVFVCNSKNFTDRGVTLAMEQNHPLLRHA
IVYAEGTRWRSIRKSIAPGFTPAKLKQMIGNLKSGADVFIDIASSHAESGREVNAFQLYQ
KLAMDFIGRTSFGIERSFQLGPEDPIAVAAKKLLKGVMRGPFHFMFQSTTTLGALVKPLH
WINMLLGAYVAIALTQETAKVIELRRRNPELRKNDVLQTLLDAEYQEDNEPPSNEAPENR
NEHAAVARGRILTSEEVLVSASTLFVAGYDSTSTALSFLTYLLAKHQDVQCKVREEVNKV
ISNNTELEYEVLTRKLPYVNQVISEALRLYPSVLTFVTRKALEDFEYNGLKYKAGTCVMS
PSLQIHRDARYWHDPLIFNPDRFSPENETTVHKIAYQPFGIGPRNCLGLRMAQMSLTYAV
ARLVERFKVELGPSHEESTFGISSRAMIAGPSNGPWILFRKSN
>Halo40802.74021
MSAAGEATLFPMTLTGFLPFSSHRVLWSLTLLLSCIVPVAVHIARRRRIAELISKIPGPT
AAHPILGNLDVLYELKKYRHLLAPHILLLQTMCGLAQIHDKDRIFRFWLGFRPVVSFFKA
ETVEVILSSNTVLDKSFDYTLLHPWLGTGLLTSSGNKWRRRRKMLTPAFHFRILEDFIPV
FNEQAVIFVKNLKEQQNKKYIDIVPLVTLCTLDIICETAMGVKVDAQLNSNSHYVRSLYE
VGETFMARVMRPWLWPNYVFYMSSFGRKFKDNLAQLHNFTRKVIRERKAELLEQKVIDGL
TIGEQVIGQKRRQAFLDLLLSHHIQDSSLTEEDIREEVDTFMFEGHDTTAMGISWAMYLI
GLYTDVQQKIHEELDGIFGEDRERAITPDDLKEMKYLECALKESQRLFPSVPFIGRELME

DVVVNGYTVPRGTTCFLFTFMLHRDKEIFPNPEVFDPDRFRPENCVGRHPFAYVPFSAGP
RNCIGQKFALMEEKVVLCSVLRNFCIQSVDFRDKIHLVAELVTRSKHGLKIRLRPR
>Halo40802.97067
MADFLSLVLVVAVLCSIYAWRKKKFSYFKEMGIPGPQPSLIFGNLLQIKKQGAAELFEEW
IKKYGNIVGFYNGAIPFLLVNDLDLLKKVEIEEFHNFAERGRVIEVQEVPDIRQKSIVTA
PVHRWREMRAVLSPAFTTKKLSQIFEIMNKCTDTMIELLEEKRVAGQSVELTLAFRRATM
DTMFNAGYGVDLDVQRSPPGGPLDQMGAGAGEVMRSVPFRGVTFFSNCFPELHHFWFLLT
WLTSRIVTPYFMLTTNLLLPVMTKRKAQQLRDKVDVLQLLLNKENTGELFKNDDHGFDGK
TKLALTKAEVIANSSFYLIAGIESTPNTMGLTLHMLAWHPEMQDRLREEIFAVLKRDGGF
TLKNVMEMPYMDMVLNESMRYYTGVVGFISRRAENDFEYNGLKIPKGLSIMVPVTYLHHD
PEVWPEPDKFDPERFNAENKHRIHPASFQPFGKGPRECLGRNFALLEMKLMLSKVLANFK
VSVDEQHHRVSSHHKEPIKLKSSFIATFVPNGVWMKLEQAQP
>Halo40802.103473
MLLAVGLLAVFTSLLVALVIWRWHHFSYFKRLGIPGPKPSLIWGNIREYHSMPHYKVVEK
WFKEYGDIVGFYNGDVPFVAMKDLEFIEYICVRNFNNFIGRGTLMITQDAHPILRRSLMN
VGPPLWKRIRASVAHGFSAAKLKLITPSLEEESSLLLKTLEEHSDSCEEVNMHQVYEQLA
MEYITRGLFGLDEHFFGNPDHPLYAVVRSVFRNLMTGPLHMVGQCTTAFGPLMKPIFCLA
NALRKFPFGTLTDETEKIVELRRKDPSLRRQDMLQNLLDVEISDQELSPDDACDKQGVPP
PRVLTAKEVALNAAALFVAGFETTATTLSYITFVLAKYKDVQDKVRQEVVDVLSKSGSLD
YDTVTKKLTYLGQVLEETIRIYSPALTSITRRAQEDFEYNGIKYKAGTCFMVPQYHLQLD
ERFWPAPFEFDPDRFSPENAPSICNGAHVPFGIGPRNCVGKQMATFEIKYTAARLVQKFR
MELGPSQKGVMEMESYSFLSAPANGPWIIFHKL
>Halo40802.88159
MGPVCIAVVVVFATLLFWFLAQRARTFTLFKRLGIPGPPPCFIFGNLQELRNKGMAATFD
NWITTYGDTVGYFHGSVPAVLTSDPEMIKQVFVKDGHKITERQVFTLVTKPLLLNKVRLT
RTFGQDWKELRGLISPTFKSLNMKQAVVIMKDRANLFTAMVEMAASEQHGAADLHKHVLR
LAMDVAVGVFHSIQTDVQRGDEAACELLDKCRRCTGMFGDSKMVLMNSISELPWTFKPVL
KYALSRGVPMDYVLGYFGPIFEERRKANGANKADLLQTLLNAESETSPSISTNGIEKASS
ENKNVHLTCLTRTLANGTLFVIAAIDAVATPLSFATYLLALHQDVQDRLREEVQLAIKKD
GDITYENVCAMEYLGQVISETLRMFPSLHGFVQRACDEDYEYKGIRFPKGTVIGVPVLRM
HYDPRFWNEPEKFDPDRFSKENKLNVNPMAYLPFGAGPRNCIAARYSELLMRVSLATLIS
KIKLSPNDGDNKTNGSFRANFIFMVPDKGIWLRVDKLPSM
>Halo40802.94243
MPGLPCSSISVYSPYATKCTDRTRARSRSPKRAARVPLTTLCGKRYKKIVSASNTIISQP
AAPTPDRQASEMISALTASRNFDRAPGLFYWTLGTIASAITALIIIHLARSLFEWVRMWW
YLRDVPKPKPKESFTLLIDLYKQLSAMGPNLDIKVKSFKYLQGLFKSVEDQDVTVAFYGP
YPFLLASTPQVVETILGDTKNVDKAFFYDMMKPWVGGGILTLGGEPWRNRRKAITPAFHF

RILDDYIPIMNKRGERLMKKVSTMNGEFFNVLPIARAATLGVLFETSMGIDYDEDEIERV
GYLKIHDAISDSVVNRVANFHHWFDRLYAFSSEYKEMRRNVEKARAFMDTILQKRLADYR
KGVRDPVSSNAFLEILLRMCVDEGKISEIDLRDECLSTLIGGFDTTATNIAHTLYLLGLH
PEIQTKLHDEIDLVCGDDWDKPLTAEDLKNLTYMECVLKESMRLYPPAPIIGRTITRDIK
IGKYTIPRGTMAIVALYFLQRHPRFFANPDAFIPERFLETKRTHTYAFTPFSAGPRNCLG
K
>Halo40802.23081
MAPVIILLLGAILLYCVRAVKRTFGFWHGKGVPYLSLTEYLGVLYDAFTKPLPDVIRNNH
LKYGKIYGSYQGFVPCLVVGDPDLLRVVCAEDFKSFVDRTEGEVTGNVLWDKMLLNMTSD
VWRSERMALSPAFTPSKLKLIFPRFLELTERLRLQFVEKAKNRNIVAVQRLFETCSMDGM
AAFLFGLDLHTSATPDHPLVTCCNGFFSAKSGWKVVMLYLMSKVFKMLPIEFPSKYGSQQ
VMKFVEVMVEKRLRSKERYNDVLQFCLDTVREVNKNNDNKLTESEMKDVAAEIMVFFIAG
SDGVAIALTFSAYNLALHPEYQDKLVEEINEAVKKNGMTFEAINSMSLLEAVVKESLRMY
TPDSFLTRRCNKETTLYGIDFKPGMCIELPLTGVHYNPDFFPEPYAFKPERFLPEMKDLL
KPYSFLAFGAGPRNCIGIRVALVQAKTMLACILRDVRFERCDETAVPVTFVPRRLLLEPT
TEVKLRVVPRS
>Halo40802.77162
MIGSTLSHSFGLPVLTKVTATASLVAQALAVFLVTSCLLVAVFLSVVKLIQRSKLRDIKN
VPMRWEPIPYMNLWSLLKSCSQAGSSAGLSASLFTAVVGFHVLYKKHGMHMFFIGTTLSV
SLQKAEFIEEVLSSHSLLAKGPEYGMLHTWLGTGLLTSTGSKWRTRRRLFTPAFHFRILD
DFTPTINFQSQILAKKLGALTDEGKQFDIVPLVTLCTLDIICETIMGTSIHAQHDADSPY
VKAVNRLGELFILRMLNPLAEAEFFFKRTAMGREYYSCLNTLHSFTRKVIAERKQLLRNE
DCTDPDYVNMPEKRKESKQKRPFLDLLINEHLIDPRSITEEDIREEVDTFMFEGHDTTAM
GISWALFLIGHQPREQQKIHDELDAIFQNDTERQVTSEDLKEMKYLDCCLKEAQRIYPSV
GFISRTCEEPFRIGGNIFPKGTMVRISTYCLHRNETVFAKPEEFHPERFFPENAKGRHPY
AYIPFSAGPRNCIGKTWHR
>Halo40802.90132
MHHCTLFGKNSVIHMLCVTKVHTNNEYIFTFMFTLTLLRNRLSFTFRWLRRTFTFWNGKG
IAHLSFWQYLRFCYDLYTKPLNEVIHRSYAKYGRVYGSYQGTAPTLVVGDPEILREIMVS
QFKNFSDRTESQKMGSDVWRKSILNLSGDEWKKARTIFSPALNPARLKKIVAKIRVVAEK
MTSKVAEAAAQDKPINISELAHHSFLDTTAALNYSVDLDSATDTEHPLLKSLEAIFSPVA
GYKLVMLFLMPRIFKLLQTDYPPKASTDLFKAFVSHLIEDRKSKNKAEDDFLQVFMDAEY
DWDNTSDRKQDNEEPKKMSLEEITAQGLVFFVAGVESVATVLTFTAYFLALHPEFQDRVI
AEVDKAVKEGGITYDALQEMHYLDASIKEAMRVVTPDSVTLRLCTEETTVAGIHFKPGMC
VDIPLAAIHRDPEYFPEPDKFMPERFLPANKESVRPFTYIPFGAGPRTCVGKRLGVVQAK
TTLACLLSRVRLEPCSETMPTLKYKPCQLLPVIDGPVILRAVPRDSTWPKKESVAQ
>Halo40802.105813

MLHAILLVLLSVVLLVVGIFAKTFTFWKGKGIPYLSFLDYLGVVRDNFTGELNKVAVRNY
RRYGRIYGSYQGLIPSLVVGDPDVLRDVFVKDFKSFVNKSDDKVSGNSLWDQMVLHQQNE
EWKHTRSTLSPMFTTAKLKSMVPKMTKSTDNVCKLLLEKIKAGKSINLYQLLEKSAMDLY
TSLIFDLDINSHVDTDHPMLKCYSGFLSAPGGWRLLMMTTLPRLFRALRVEFPNKGDAQY
AVDFTRHIIEKRLRDKEVHDDALQMYMDSKMQPNGKGGAALSESAINDIAAQCMCLFIAG
SDSIALTVTCAAYSLALHPEIQEEVIKEVDGAEEPTYEALRSMVLLDAVVSETLRLYSPT
SVLTRICSQPTTVAGVRLTPGMRVEIPAHAMHYDPEFFPDPESFKPERFLPANKDGVNQY
AYLPFGVGPRSCIGMRMGMVQVKHILYRLLQTAKFEPCSETQMPLSFAKGKVLLEPDPPI
QLRVVPRAKT
>Halo40802.96851
MYRRYGPVVVEENPGCETVVHLFHAQDFRFVFRYEGKVLYRSGLHPLKAYNTAKRELFPF
SVVRHCETEEWGYVQSRDRPVASQPGDIESYARAMGQISDDTLKLIAALRDDKSEVEDSS
AIMRRWSLESAMFQALNSRLGLLEHHVPGDSDGRVILRCTEDLASIMDSLVVSWPFSHYF
HGSKVEELNTIADELAPRLLRVSKETAPQACFKVDSSGNDRILELLQIKLSLDEMFPFVH
DFLVANTYTASAAATFCLYRLAMHPEVQEKARQETLGVLRDSPEYYSPGNLPYLHACIIE
SLRLHPMVYRMDKKVHVDFVASNHKVPANTLVRATISVAGRLEENFPNACEFVPERWLRT
DESEIFEEVRDVWYQHQFASIPFSMGPGMNNSRRFIELELLILLAKVLRKYKVESCHADL
GFQTRFLSAPQHAAKFRFLDLETST

Hyalomma asiaticum p 450 sequences

>Unigene0091162

MLGVFWRCLVTGLVAGVGAVVLQYLWDTLKRCLRRDLPPGPFGVPLLGYLPFMPKDGHRG

VEALRQKYGNVFGVHLGSRYVVFLCDFESVKEALSQDSLLNRPEEFPFKVNQDSQGLIVL

NGPLWKEQRRFSLKLFKNLGIATQAMEKHIHEELSYLLRELESVKSGRIVPTSVLTPSTS

NIISALVFGRRFEYDDPERAYLDKLIEVIPALAAQVSAINFFPWLRKLFVFFRMGACEQL

RDALVRREDFADSKIDSHQDTYQDGLVRDYIDGFLSEMKQQDEGSKTFTRNLLKGNVASF

FGAGSETVRAAIEWLLLVSAMKPELQRRIHDEIDAVLASGESSQISWCNRSQMPYTQAFM

WETMRCKPVNPLSLMRYAATDVKVGKYIIPRGSVVIGSIWSIFYDPAYWGDPEVFRPERF

LANDGTRPQKPERFIPFSYGKRACPGEVIANMETFIYFTTILQHFTVEEPPDGPKLVLDE

VLGISLRPKPQELLFRPREVRV

>Unigene0040524

MDWVTIAGSLLAASLLYVITRTWLSSKSVPRGARLPPGPPGRPLVGHVQFTHKDFHCNQA

MKWANQYGPVYRIRTGSADMVILNDFASIKKFLTKKEVLYRPQNWLFRGEVYGGVATMNG

ETWVDNRRFCLHVLRDLGFGKTSMEEHVKDECQCLVEKIAEAKGAPIAIQEYIFPSTSNN

IAALVYGSRYPFEHPRRRYLDELLSELFKAIRAGTLVEFLPSFVRKAITWLPSTRRTVIK

SKLMEFIEYTKGQVADHKATMDEHFNRDFIDGYLKKIQEHENEQNPNFQHRFLLGNVLSF

FIAGSNTVAVTIHWHMLNFANNPDTVQARVQREIDEVVGRERQPTWEDRNKMPYTMACIW

EMYRWKTVSPLGVPRGAGEDTMFDEYFIPKGTTVIPNVWAVHNDPTLWKEPSKFDPTRFF

KEDGSLVQSKPEHLIPFSIGKRMCPGEILASVEIFLYITCLLQKYRILPEEGKIHDLDSI

DIPLVELNHYKLTFTPR

>Unigene0005615

MAVWFTIIASGILALGNLVSKRRGNKGKRLPPGPSGVPLLGYLPFMKKPYYVDFKKLSDR

YGPVFRVRLGCKNVVVLSDVTSVREGLNNPDVLYRPEDFLFRYLDNKGIGALNGEVWQLN

RRYCFQVLRNLGFAKKPMEEHIKEEIQRFTELLEASKGKPVNVVHELVVSVVNNMSALVF

GERYDVHDSRARLVSDLMSKFLKNADFFSLTDFLPTIRFFSLYVPHTRLRTINYVFKEFK

KLFRQEIKNREQVMDECKDKDFIDGYLRKIQENNGNGSHYNLDVLEGNTFNFYSASTNTV

RTAILWNLYIVASDPDGHQARIQGEIDAVVGKLRAPAWDDRLRMPFTMASILEALRWRTV

SPIGINRAAARDTVICGYHVPAGTVVVANFWSLHNDPAYWHSPSKYDPSRFLTADGTEVK

EKPMAFLPFSMGRRGCPGEGLAMMEIFLYVTTVLQKFRVLPEEGRAISLDGYDALLNVVD

DTQGLRFSLR

>Unigene0070178

MCLFACVVLVGALVIYLKGRGGGRRLPPGPPGLPIIGNLLSVKRGFYFHDCIKWAKKYGP

VLWIKFAFTNVVILVDETSMQKYLCKREMLNRPSNWTVRNKSKGIVALNGQAWRENRRLC

MQILTDLGYGKEPMHIRIQEEVQHLLSKIAEQGGKPVRSHEYLKGSVLNNILLYLFGRRH

DLDDPNRRQLDDLVGAFFSAGLDFSIEWLPGWLRRASQRLFPTMRCSTVTRLTENITDYM

RQQIEHHQKIPDSQRQSCLVDSFLKEIENRKSDDLTMSHLAGNASDLVVAGTLTTTMTLQ

WHLLQLALEPHGLQEKLQQELDAVVGRERAPAWEDRERMPLTMATIWEMYRWKMATPIGL
PREAAEDTWVGDHFVPKGTVILANLWAAHMNPDRWDRPEKFDPTRFLKPDGSAPVTRPTN
MFPFSVGKRMCPGEPLAHAVVFLYLTSLLQKFHILHEEGSQPDISGAVIPSKVAGIRLRF
LPR
>Unigene0042213
METGAFGVTAALLVAVVVLCASWAVKRKHQQRLFQRYGIPGPVPDFLFGNWNQLREDRIE
VMEKWIKEYGKVFGFYEGEIPKVVISDLNMIKECFVKEAHVFRDRMPLIIEVEPIKSSLL
ALRGEEWKRVRSVLNPAFSGAKMKLMLQVMNDCGNTLIDVISERLTSGQDIIDVTKRSQA
LSMDVITKCALAWQVDCQRNADDPTLQLLQKVFLEVDERTLASAVAFPVLRKVYAWLLPL
LKYGRVLLQVADNVLEVIRVRSAEKQGKQASSVDIIQLLLDSRTEAAQKGSGKVEITDRH
LIANCFVFLAGGFETTATTLAFLLYELARHPDEQERLYEEIKSQLADHSVNIPYDDVQKL
KRMDMVVSECLRLYPPIVLFTARVCSRDTSIAGYTVPAGTHVILPTWHVHHNPNIWPEPQ
KFIPDRFLLGIGEEERRHAAAYVPFGLGHRECIGRRFALLELKTVLAKLIRAYVFSVCRE
TDDPMKLTVP
>Unigene0011285
MIRKSLLLSYDAFSWTTIAIAVVYVLYRLVRYRQKTFSYFKEIGIPGPEPSLLWGNLAEY
HRKGFVHAITDWCAKYGDVFGFYNGDLPVLVVKDLEFLSYVFVKDFKNFTDRGVLMRTDQ
EHSVLGNSIIHVRGANWKRTRSCMSHAFTSNKLKHMMQDLFTSTDLFIETLGKVADAGKE
FAMYETLQGLAMDYTGRAAFGLDCCFQRELSHPFMETARKVVHGVMTGPFHMIAHCTSTL
ADIVAPLLWLNEKLGSFTFSIFGVETSKVVEMRLRNPEARRQDLLQTMLDACEEKKRPND
GDAKSRANMTLQEVELNTTVTMIAGFETTSAALAYVCYLLAKHEYVQEKVREEVITAIGE
CGKLDYDAVTHRMKYLRAVVDETLRIYPPATLFTTRRAANDFEYNGVKYKAGTSIMAPTR
QIHMDHRYWPEPHKFDPERFLAENASSRSSIAYQPFGVGPRNCIGERLAILAIIYTVARM
VEKYRLTLGESQEKDLELHFYAAMCAPQNGPYIRFQRI
>Unigene0023963
MYKKFIDGYATARAKLHLLSETATWLVLSYLAWVLLVRPTVVWLRKWNALRPLPGPCDGL
PFWYSIRTYWEKSRSNSVKDATCVFFQIICDVCERYRGTTFKAYLGMTPVVVLHTPDAVQ
TLLTSKTNLRKPIVYNFLASWLGDHNTLTAVGDSWRFKRRLMTPAFHFKVLDSYINSFNY
NATLLVKRVDRLTAKHPDEPIPAFRLSQNCALDVITKVLMGVDLGSQLETTEPTFANHFN
MLMFLIGVRIFRPWMWPDFWYARTSEGRLYYHSIREMEKYTLEVLERRKGKLQQIAEEME
SMPGDYGEQAGDQVQESVIVDRFLKAHLQDSRYSIGEVKKDIDSLLFAGTDTTTSAVGWA
FYMLGLHPKVLAKVHQELDDIFGSDTERDVTAEDLKRMKYLDICFKESLRLFPAAPIIGR
LLDEDMVVDGYRIPRGVTCFVNIYSLHRNPEYFKDPELFIPERFLTPEVRNRHPFSYIPF
SGGPKNCLGQRFALLEAKTLMAKVLRKYTLESTRPVSELRITYEVILKARGGLRIWFRNR
AHVERATKLTDFEDQETNYGL
>Unigene0059299
MWELVIAALVLVIMHWLMKRREHFNFFKNLGIPGPTPNIIFGNMLEMYKKSPAKAYREWI

DKYGDVVGYFNGYRPVILVADLELLKQIQIKDFQDFVDRGLLFQAKRPPSPHNKGLLQLT
STRWKEVRSVLTPSFTTNKLKMMSPGVISAVEKLVTKIDRKAKEGEEFEVGEMYAALALD
VICKSAMGIDYNLQDNPQHRFLVCCRRLFGCAFTVIAVLLTAFPGLAVLLIFINFRLLRF
QNSGVHPFVEVQEKCKKIVQERQLDSSLHRKDLLQLMINAKDSHVDVASVTSTQLTAAED
NEQELSSGARNGSANDNVSGGFSKAVLDDDDITQNAFSVLVAGFETTSNTMSLVTHMLVH
YPQVQEKVRKELLSVLKDGEPITYSTIQELPYMNCVILETMRLYPPAFAFVTREALRDKQ
YDKLRIPAGTVVMAAVEHIHRDPRHWENPDRFDPDRFLPENKPKINTMAMQGFGNGPRNC
IGMRFAHMELRYTFAHILRKYRLEKTENSEKDPPSIEMNPIILKIKNGVRVKAVPL
>Unigene0049525
LLLHQPTTFEFSYLLRALCEMYKDRTFKAYIGFSPFVVIQNPENAEVLLSRTENLRKPFF
YTFTIPWLGPRNMLYIAGNVWRFKRKLQMPAFHSKTLEKFMGVVNEHADSMVERLEEASI
KKELAPIQEIVKRCSLDLISEVLMGVNLNTQKNQNSQYGEYISGVTFLMAVRAFRPWMWL
NSIYNFSIEGMWFRKMISGINEFNLKIMGERKKTFHLYRNQGDLSGSDDEDAKDKREHQP
ERRMAVIDIMLDKHFKDPSYTMQEISKDLDLLLFAGHDTTSIAMAWTLYLLGLHPELQRK
VQEELDAIFMDDVNRDVTRDDTDHMVFLEACFKEAMRLFPPIPLIGRVLDESIKLDGVMI
PKGTTVFINIFTLHRNSNHFEKPEEFVPSRFLDGADKKRHPFAYIPFSAGAKNCIGQRFA
YRQAKVVLAKVLLRYTFKASWPLDRLKLSTEMVTKVKGGLRVWVRHRKPGQYA
>Unigene0030066
VSQSTPLSSIALAQLATWQALAWTAAGLVVLWFVKDYYLPWLKTWIALKPVPGPWDWIPF
WYLLSVYWERRKQLNADSFTAAVFGAVRDICKAYEGKTLKGYLFLTPVVGIHTPEAAQAI
LTGKVKSDKPVLYRFLKPWLGHRSLLMIGGDAWKTKRRIFMQAFHTSAMDGYLGFMLDNA
ECLVARIDQLLKEAPGEPIVCLPNAQKCALDIIGRFALGEELGVQKEKHENYGTYFHMLT
VLISSRTFQPWNWLDAVYNLTHSGRLFQNTLQRIEAIVQGVLKNRRNVLQKLHHETKDNG
GQTLPNSDGGGSLFLDSLLLAHIKNPSYTLDEVRKDADFMMFAGSDSSSCAISWSLYLLG
LHPEKQRKVQQELEDVLGHDPDRIYTLDDLKRLEYMECCIKETLRLCPPFPFIGKVTDED
LVIDGHTLPKGLSCFISIYSLHRNPNQFEKPDEFIPERFTSEENNRRHPYSYIPFSAGPK
NCIGQKFVMMEVKVVLAKILTKFNVESTRPLEEVEMTFEIVLKAKGGLPVWFRRR
>Unigene0089213
MELIGLPDWVLLAATAVILLYLYASRHRNYWKKQNIPQEDFSLIIGPTTRIFFQPFHTID
TERYHKFGRVFGIYEGGKPTLLVGEPELVKQVLVKDFPLLCNRRQLQFFDPILDNMMSIA
PVERWRKIRPSASPAFTTGKLRRMNELIQACAEITTEHLKNAAQQKKDIDVKQFYGHYAL
DVIARCAFGTKLDSHTDATNEFVTKARKAFSGGVTLPLLMLFLFPGLMKALKVKAFNAEI
FQYFKEVSVNIIQKRKEKHCRQEDFLQLMMDAQEGALQEAPESTTGKESSEIFNLDSEIK
NDINFVSKALSEDEALAQCVLFFLAGHDTTSSVLSYAVYLLALNPEVQAKLRKEVDECIA
THGKEPSLDAVSKLPYLHCVVSETLRMYPPVPRLERSAYEDYVLGDSGIRVPKGCVIGIP
VYAMHHDPEFFPNPETFDPDRFSEENVGSIRPYSYLPFGAGPRNCIGMRFALQAVKLSLL
HSIQSVQFVPTDKTEVPLKFLTGMGLLIAKDITVGIRERPER

>Unigene0008037

EISGRNETSVFVARGNIICSLFSTNSTGDKWRSRRRLFTPAFHFKILEDFSPTINAQSFI

LCNKLAKLSKNGKSFDIVPVVTLCTLDIICETIMGSSISAQSNEKSPYVAAVNRLGELFV

ERTMRPLLQLDFMYKLTASGREYYKCLNVLHSFTRKVIEERKKELEAEVNDGALVLDSTS

KSEPRAKRRRPFLDLLIVEHLKNDKYITKEDIREEVDTFMFEGHDTTAVGISWALFLIGH

HPQEQQKIQEELDQIFGKDKERYVSFEDLKQMRYLECALKEAQRIYPSVPIIARTCEEPF

KIDGATLPAGTIVQMSAYFLHRDPEVFPKPEEFHPERFFPENSKGRHPFAYLPFSAGPRN

CIGQRFALAEEKIVIANILRHFTIKSLDQRDQVELASEMVLRSRNGLRIKFIPR

>Unigene0072704

LTSLNAAMLFLFGTLSLAAVFLAVVYRWRKRHFSYFKALGIPGPEPSLLWGNIREYHETH

HYKVIDKWLQKYGDTFGFYDGDVPFIVTKDLDFLEYVLVRNFQNFTDRGDELVMEQKHPL

LGNAIVYAEGTRWRNMRRSVASGFTPAKLKLMMADLKKGADIYLDIAGEHAELGREVNVY

ELYQRLTMDYVGRAAFGVDGSFQRGPENALAASTKVVLRGIMKGPFHFLCQSTSTFGALA

KPLYWINMLLGAYVAIAMTKETGSVIDLRRKNPEFRKPDVLQNLLDAEYQEDGPEIQNGT

SESVKGHAKVLKGRVLTKDEVLLNACTLFIAGYDTTSTSLSYVTYLLAKHQDIQDKVRKE

VDTLCNTEDLDYESVTRKLPYLSQVVTEALRLYPPVLTFISRKALSDFDYNGVRYKAGTC

FLSPTLQIHRDPRHWPDPLTFNPERFAPENEGRYHKVAYQAFGIGPRNCVGMRMAQMALN

LTIARLVQQFHLQLGPSQGEEPLDIGCRAMMSEPAVGPWIVFRRI

>Unigene0023028

AATNGGSTMSAEEKMTATSGSMPKATVVLAVLFAALLAKIIVAVARWTRMYWCLRNVPHP

KQRWPFSLAIDMWQCIANMDPNLEVTAKIFNYFDGMFKTIYDQEVTAAYYGPQPFLIATT

PKAVESLLTNSENLNKAFLYGMMKPWIGNGILMIEKGKWRSRRKILTPAFHFRVLDDYAP

IMNNRARQMVSRLDAMGTDFFDVLPIIRLAAFGILFETALGVQIDEEEVQKMRLLEINDE

IGASVIARMLNLLHWPDIVYNMSQASKEFRKNINFIHEYNRKIVKQRLSEFKMGKVMAGS

KKSFLDILLHMHLVDGTLTEDEVKNEVTSIFIGGFETTAVSIAYTLFLLGNHPEVQAKVH

EEIDSIFDEDMDRDVTAEDIKQLKYLECVVKESMRLYPPVPLIARDVDEDMKVGEYTVPR

GSVAVAAIYFMQRHPRYFDNPNTFLPERFLDNKEKNPFLYIPFSGGFRNCIGQKFANLED

KILLTQILRRYTVSSKLRMDQLQLSIEVVLKAIQGLEIKIQLRNKTAK

>Unigene0001716

MDLANALWQLFAATIILLLVHWYMKRKQMFNYFKDLGIPGPEPSIITGNMDELRKKTPTV

AYREWIEKYGKVIGYFNGSRPVLLVADLDLLKMIQVKDFQDFIDRSLLFQCKRPPSPHNK

SLIQLTGKRWKEVRSVLTPSFTTNKLKMMAPGMICTVREFVDKIAEYARSGEEFEIGNLY

QAMTLDVICRSAMGIEYSIQKNPTHSLLVCCRLLFNSTFSWIAVLLASFPELEFILKYLN

DWRLAATNNGVHPFQEVQEKCGNIVRQRQIDHSAPQKDLLQLMIEAKSSNVDVASVTSDQ

LTAADDNEHELKQHAQHSPNGLSYSSKTVLDDDDITQNAFLVLVAGYETTSNTLTLVSHM

LINYPEVQEKVRQELLAVLGPDEEISYNTIQKLTYLNCVIQETMRLYPPIFAFVTREAVV

DKQYGKLKIPAGTSVMAAIEYIHRDPSSWEKPDTFDPDRFLPERRKGQNPLAFQPFGAGP

RNCIGMRFAQMELRFTLAHVLRKYRLVATPNSDK
>Unigene0037076
MWAEVLFSLLLVLVSASLSWFLRRKWKLGLFKRHGIPGPEPDFFWGNFLQLKEDRVQVME
RWIAEYGKVFGYYNGEMPYIVVSDPEMVKQCLVKEFPTFHDRAPFVLNVEPFASCMLVLT
GSEWKRVRSVLNPTFSSVKMRQMAPVVHSCVETMLEVLDERCRAQQTVDMLKVAQGYSLD
VITKCAFAWQVDCQKNPNDPLLLGVRKIFEEAESTAVCNAIRFPLLRVVLTYLYRLSDYY
KVMHRMIDNLRQVIELRRRESKVSATDMLQLMLEAQAGAEVSNNRTIGREVRLIEDRHVV
GNAFIFLAAGFETTATSLGFLMYLLATHPDEQEKLHDEIEGAFGTDRELSYERVQMLKRL
DMVIQEALRIYPPVVLFISRRCDKDTTIMGQFFPAGVNIMVPTWHLHHDPALWPEPFEFR
PERFDPDGAGGLSQHQAAYLPFGLGPRVCIGKRFALLELKMAVCRVLREYRVVRCEETQV
PLKLIVPSVIINPERGVVVKLERR*IRA
>Unigene0054495
MLAAMYYVLVIGKLAAAIALAIIAFLCGKLALRWIRMYYYLRQVPHLEERWPFSLLLDTW
TALQQMDKNLPITAKFFNYIEDIASRICEQDVTIAYYGPQPFLLCISPTVFEAILSSNVN
LNKSFIYRMMKPWMGNGVLTSDKEIWRTRRKILTPAFHFKILDNYVHIMNRRTREVVGKL
MQLGEKPFDILPLLRLAAFGMLFETAMGVNINEEEVTNKKLLHTTDELAASVISRVLQVF
HWCDAVYYMTKEGQLFLKRAHYIREYNRKIIQQRKEQYLSGEAGCAPRKSFLDILLHMHI
DEGTLTEKEVAEEVATFFIGGFDTTATATSYTLYLLGHHSDVQEKVHAEIDAVFADDKHR
SVTTEDIKKLKYLDCVIKESLRLYPPVPLVARNIDEDVHIGKYTIPKGTVAACAIYFLHR
HPKVFKEPNTFMPERFMGSTNVSPFAFVPFSAGSRNCIGQRFAQLEEKIMLTHILRNFTV
ESLVPIEELQLQLEIVLRPLQGIQLKLTPR*QMSPH
>Unigene0026123
MFVPLSSESPSLSSQFDALLARVSPWLVAFLAWVFLARPLVQWLKIWVALRPIPGPWDGI
PFWFSVKAYWTTRKTIGLKDATAGFLTIVNELTRQYQWKTFKVYLGPMPVVFVHTPDAAE
TLLTSKENFRKPFVYRFLSSWLGPHNLVTATGDIWRFKRRLFTPAFHFRVLENYICHFNE
NGNVLIKKLEKHIDEKPNEALATFPLMQHLTLDIIGRVSMGTVLGMQTDSVNPFGKNLNR
LSFMIVLRGFRPWMWIQKIYDMTYEGKVFRETLLDMEKFSLSVMQQRKEKLQEMELNHEG
DGNSGEDEPDEEDEKNSKPIGKESIVLDYLLKKHLEESSYTIDEVKKDIDTIIFGGNDTT
TSAMSWAFYLLGLHPDKQAKVHAELDEVFGTDRDRDVTKADVNQLKYLECCIKETLRLFP
SIPLIGRHLEEDLVIDGYRIPKGANVYINLFSLHQNPKYFKDPESFVPERFLTEEFTARH
PFSYLPFSGGPKNCIGQRFALLESKVIMAKLLLKFSVESTRPLNQLRVSYEIIVKARGGL
RVWFRRRPPVNDEGQVSTT*TLC
>Unigene0059300
MWELVIAALVLVIMHWLMKRREHFNFFKNLGIPGPTPNIIFGNMLEMYKKSPAKAYREWI
DKYGDVVGYFNGYRPVILVADLELLKQIQIKDFQDFVDRGLLFQAKRPPSPHNKGLLQLT
STRWKEVRSVLTPSFTTNKLKMMSPGVISAVEKLVSKIDRKAQTGEEFEVGEMYAALALD
VICKSAMGIDYNLQDHPRHRFLVCCRMLFGCAFSFIAVLLTAFPGSAGLLKFINSRWLRY

QNSGVHPFIEVQEKCKRIVGQRQQDTSLRQKDLLQLMIDAKESRVDLGSVTSTQLTAGEE
NEQELSNGANSTNQGGSGNVSVFNKAVLDDDDITQNAFVVLVAGFETTSNTMALVTHMLS
HYPEVQEKMREELFSVLAADEPITYSTIQKLTYMNCVIQETMRMYPPAFAFVTREAVVDK
QYGKIRIPAGTAVMAAVEYIHRDPRHWENPDKFDPDRFLPENKSQINTMAMQAFGSGPRN
CIGMRFAQMELRYTFAHILRKYRFEKTENSEKDPPNIEMNPIILKIKNGVKVRAVPL
>Unigene0018453
VVGALLVIILTVTVLLLMWRRRHFSYFEELGIPGPKPNLIWGNLREYQSMPQYKVLEKWF
QQYGDVVGYYNGDVPFVALKDLGFVEYVCVRNFQNFVDRGVIMPTNEMHPALGRSLMNER
APGWKSIRRAVAYGFSSAKLKLLMPTIKKNADMFLESLAKHADSGEEVNMMSKYEELSME
HITRGLFGLNEHFIGKPNHPLTVIAKTVFRSFMRGPLHVIAQSTTTFGSLMKPFYFLINT
TAEFPLQTLSDETEKIVNIRRKDPSYRRPDMLQNLLDAEYFEECTGSSSAPVSNGGSDMR
PLTTQEVVVNAATLFVAGFETLAAAMCYVTFALAKYPDIQEKVRKEVSDAVPASGSLDYE
LVTRKLKYLGQVIDETLRRYPPALTSITRKAKEDFVYNGKKFKAGTCFMIAQYHLHMDPR
FWPSPEELDPERFSPENSTVLNKCAHVPFGVGPRNCVGVRLARLQLKYTMARLVQEYRLE
LGASQKGTMDMEAYCFVSAPARGPWIVFYKL
>Unigene0066938
MAMFFGIPDWAVLLFTLAVVVYLRVSRYRNHWKKQNVPHESYALIFGPMLRIVNKPFHEL
DLERYKKYGRVYGMFESGKPSLFVAEPELLKQILLKDFLVLPNRRTANFFEPLFDYMLSV
IAYPRWKTIRKYYSPAFTSGKLRKMEHQIEDCTKSTMIHLQKAAEEERDLELGHFYGNFS
LDLIARFAFGTRLDSHSDQANDFVTYAKKVFGADFSVSLIIHFLLPGVAQFFRLKLHNPA
TQEYFKSLCERVIKDRVDSKTRQDDFLQHMIDSQQGKCAADTSKETADVAEKIFDVDSKL
ADSEETQSNALTAEEAMAQCFMFLVAGQGTTSSLVAFALYMLALNPEVQEKLREEVDQCV
EKHGEYPPMEVVTKLEYLHGVISETLRMFPPASRLERETTKDYVLGETGIMIPKGCVVAV
PLYAMHHDPEYFPDPHTFRPERFIGDNLANIRPYTYLPFGAGPRNCVGMKLGLQASKMAV
LQSVRMAQFVRTDKTKVPLDFFKGFGVISSSDITVGVRKRTTTNK
>Unigene0001843
MPLTDHERYQKMGKLFGVYEGGIPKLVVAEPDLVKQVLVKDFHLLPNRRVHAISDPIFHN
MMAWAPFEIWRKIRPAVTPAFSTARLRKMNALIQDCVRVTCEHVKAAAEQRADIHVQQFY
GHYSLNVIARCAFGTTLDSHSDATNEFVTEARKGFSARVSWKVLLFVLFPGLLKYLKINA
SRGGTSQYFKEACQRIIRERRENGKRLEDFLQLMIDAQDGNLTASEEISADLENKICDVG
SDTKPGGPISTKRLTETEAMAQCVLFFLVGLETTSMTLALATYHLALNPAVQEKLRKEVD
ECMAEHGPEPSLDAIMSLKYLHCVVLETLRLYPPAPRVERYAIDDYVLGETGIKVPKNSS
VIVPIYAMHRDPAVFPDPERFVPERFSDENVESIRPYTYLPFGAGPRNCIGMRLGLQSIK
LCLIHILHNVQFVRTEKTKV
>Unigene0065845
MALPGLPVWWLLAVTLCVVLYLYAARKRNYWKEQNVKHEPLSLLFAAAKRLLLKPAHIVD
QERYQKMGRLFGFFEGGEPILMVAEPELVKLVLVKDFTSLPDRMPMQFFEPLLDNTVGVT

PLETWRRIRPSVSPAFTTGKLRKMTDLIQECAKKTAMHLTEAAEAGMDVELKQFYGHYAM
DVVSRCAFGTAVDSHSDKTNEFVTRVRTVISGGLNFRLIVLMLFPGLVKALRMRVFDPRA
FEYLRSFTLTVIKSRQNERHEDFLQLMMDAQEGTLSDSGDGAAEANSEVFNLGLDSKCNV
LFGARRLTETEALAQCVQFFLAGLDSTSSVLAYTAYLLALNPDVQRKLRSEVDDCIKSHG
NNPSLDVVSNMKYLHCVLSEALRMYPPAPRLQRIASTDYVLDETGIRLAKGCAVAIPAYA
MHHDPSFFPDPDRFDPDRFSDENVGTIRQYSYLPFGAGPRNCVGMRFALLSVKLCLLHSL
RCVEFVSTSKTKI
>Unigene0023029
MISTLAAMRTFERAPGVFLWALGAVVWLLTAALVVRVTRAVAEWLRMWWYLRDVPKPKPQ
KSFTLLLDLYKQLSAMDPRLDTKVKAFKYLANLFKSVEDQDVTVAFYGPYAFLLGATPQV
VEAILGDTKNVNKAFFYNMMKPWIGGGILTLGDEPWRTRRKAITPAFHFRILDDYIPIMN
KRGERLMRKVTSMNGEYFDVLPVVRAAAFGVLFETSMGIDYDEEDIQRTGYLRIHDAISD
TVVDRVINFHHWFDTIYAFSKERKQMLQYVEEAKEFVNSILQKRIADYRKGIRDPVSKNS
FLEILLRMCLDEGIMSEIDVRDELLSTLIGGFDTTATSLAYTLFLLGHHPEIQAKVQEEI
DIVCGDDWDKPLTAEDLKNLTYMECVLKEAMRLYPPGSVIGRTVVRDIKIGKYTIPRGTV
AVVALYFLQRHPRYVKDPNSFKPERFMDSKSMHTFGFAPFSAGPRNCLGQKFALREEKIL
LTHIIRRFNVSSKVPIDELELAMELILKPIQGLEIKLTPRKDPSSK
>Unigene0022630
YVTRNRNYWLKQNVPHEKLSLIIRPLYKVLTKPICLADQERCRKYGRLFGYFEGIKPALM
VAEPELVKQVLVKDFNLLPNRLEMNFGDPILNNMMVFARVDRWRRIRPASSPAFTTGKLR
KMHELIKDCVKITCEHLGVAAEEEKDADMKKFYGHYAVDVIARCAFGTKLDSQADATHQF
VKASQEAFKMDFSPTQLISMLFPGLLESLNISVNTSMRYTYFRELFLQIMDERKEHNRRI
EDFLQLMIEAKEGRFATVTASSADAESKLFDMGSEMKSDTKSSSKALTEDEALAQCLVFF
LAGQDTTSSTLAGAVYYLALNPTAQERLRKEADECFDTHGPEPSLDVISKLKYLHCVVSE
TLRLLPSVPRTQRCAAQDYVFGDTGIRLPKGSPVVVPIYAMHHDPEVFPEPEKFNPDRFN
DDNVESIRPYTYLPFGAGPRNCIGMRLALQSIKLCLLHSVHMVRFVPTEKTQVPLQIRQS
LGTLRIDGTVVGIRKRLDTRA*HSTC
>Unigene0057515
MWQSVVSMDSSLEMPAKIFIYVESMFRDIVDQDMTVSFHGPMPLLLAATPSVAEKVLSGT
ENINKSFLYTMMKSWIGNGLLTSDKAIWKNRRKALTPAFHFRILDEYVPVMNRRAVTLTD
KLAHIGREYFDLIPVMRLTTFAILFETAMGVKLDEEGVENSEFLRVNDEMAASIMTRMIT
VHHWPDLIYKRTKEGRKFYEKVGLIKKYTQDIISSRKKAYKIEGADAARKQSFMDILLRM
HLEEGVFTEEEIREEVNTFMIGGFDTTAMAASFALHLLGNHPEVQAKVHEELDAVFGSDE
ERPVTTEDIKQLKYLDCVIKEALRLYPPIPLIARELGEDLSVENHTIPKGAVSLVLIYFM
HRHPRFFSQPNAFLPERFLDGDRHPFLYIPFASGARNCIGQKFAQLEDKILLAQILRRFK
VQSMIPNEDLQMSLELVLRPVQGLYVTFTPRDRTAA
>Unigene0040533

MEPVPPMYKPLIHSKIVSTCVYVAVIAVILYCARILARTIRGYACVWNIPHPPERYPFCL
LLDLWRSVATMDSSLEVPAKIFNYLDSVFYEILDQDVTAAFYGPKPFLIAVTPDTVERVL
SNTENVNKSFLYNMLKSWVGNGLLTSEKSIWKKRRKVLTPAFHFRILDEYVPIMNRRAAL
LCDKMAALGRDHFDVLPVMRLATFGILFETAMGITLDEEDVKKTGFLKVNDQIATAIITR
MMNIHHWPEFIYKRTEAGREFYEKVELIKKYTRDILSCRKKTYKVEGDQVVQKKSFMDIL
LRMHMEEGIFTEEEIREEVNTFMIGGFDTTATAASFAMHLLGNHPEAQAKVHEELDAVFG
SDRDRPVTREDIAQLKYLDCVIKEALRLYPPIPAIARKLGEDLTIEKYTIPKGTVAVIFI
YFMHRHPRFVSKPNDFVPERFLDFDKERHPFLYIPFAGGARNCIGQKFAQLEDKILLAQI
LRRFKVESKTRNEDLQMSLELVLRPTQGLHIKLTPRDYAVT
>Unigene0014532
MMLYAVILALSFSLLTALFIWRRNHFNYFKKLGIPGPEPSLIWGNLVEYHSMERYKVLGK
WIEKYGNMFGFFNGDAPFVVLSDLDFIEYVYVRNFQNFVDRGFNMMTDQMHPVLGKSVML
VGGSEWKDIRSTVAYSMSAAKLKMMMPHLEENADIFVRSLEEHADTGREVHLLPEFEQLS
MDYTARGAFGIDEHFQGQPHHYLIKVAKEAFRGIMKGPLHMIAQSTTMFRRWMKPFYWLT
VLIGEFAFDKIGKETTKVIEMRKKDPSLRKPDLLQSLIDAELANNSTDRNGVPRTNGTYK
PRQVSSASIATSATVVFLGGFESTATSLSYIAFNLAKYPHIQEKARKEVLEVLSTSGKLD
YDTTMRKLKYVGYVVDETLRLYSPGLLFVTRKAREDFEYNGIKFKAGTAFMVSQYHLQRD
PQYWPNPEEFDPERFAPENSASLRKTAHAPFGIGPRNCVGKRLALLSLRYTVARMLQKYR
LELGPSQMGSMELGAHGMVSTPARGPWIKLYRV
>Unigene0086909
MSATAVLLLLAVFLLVVLFTLRWWHFSYFKRLGIPGPKPNLLWGNLMEYHSTHLYKAVGG
WIEKYGDMFGFYNGDVPFVVTQDLELIEEVYVRKFQNFMNRGLTMMTDQMHPYLGKSIIH
VNAPMWKSIRNSVAYGFSAAKLKQMMPFFEEDVNFLLKNLEKCADTGEEVQMMRKYEQLS
MDFVARGAFGIDERFQENPDHPLFDMAKTACCQLMTGPFHMIAQSTTSLGPLLRLVCWLS
LAIGDFVFDTVTAHTSKVVEMRKKDPSLRKPDILQNLIDAEYVESQTGAGSGKGENGVPK
TRALTTEEVITSAATLFIAGYETIVTSLSYLTFTLAKYPDVQEKARQEIEAVIAQGGELD
YQTVMKKLNYLEQVMNETLRLYPPGLTFVTRQAKADFEYKGLKFKAGTCFMVPQFYIHRD
PRFWSNPLDFDPDRFAPENEAKLKKMAFCPFGIGPRNCVGLRIATLQTKYTIAKVLQKYR
FELGPSQMGKLDLSSRAMVSTPARGPFVVFHRLNK
>Unigene0034459
MLLSLWIAVLVALFSTYIWRRRRRFLLFKDLGIPGPEPSFFSGNTAEILKKGSVKAFDEW
TKKFGDIVGFYNGGTPVLIVKNTELLRKIQVKDFGNFASRGVVSVASRHHRIARTSLTNA
PSERWKEMRSLMTPAFKSSSMKSMLSLVESCVDTFMEVVATKQTAASMEVRELFQRLSMD
IIARSAFGTETGIQRSQGGTAADTLLALIQDRLGEYKNGWLMYFANCFPEFHYLWRFLFS
RSKTSVMTPTDYIKNNLASLIEERRANKQVEHEDLLQLMLNAEEEADSLIDVQQLTVAHE
EEEVVRESLESIPVIRKRRFMTTTEIQSNAVMFLVAGFETTGTTLSFTSYLLAKYPDIQE
RTRSEVLSVMKAEGGLTYDSLTKMRYLDQVISEALRYYPVVVGFITRKCEQEYEYKGIKI

PSGMSILVPAYQMHHDPKLWSDPEEFVPERFSPGKRGKIEPMAYQAYGNGPRNCVAMRFA
QLVLKFTLAKLLSTYRLVLDPNKHEGDLKIGSSFTLAYPLDGVWLKLQEVHES
>Unigene0013814
VLQGSAAAFKEWLEKYGDIVGFYNGAFPVLIIRDTELIKKIQIKDFGNFHSRGVSSGFAR
VHPINKRNLVNAPGERWKEMRSLLTPAFTTSNMKKMANLMDDCTNEFLDVLKSLQAQNKV
FEARELFQRLTADVIIRSAFGLKSDLQQKKGSNSTTESLFQDSLRSFEQFRHAWMSYFTA
CFPELAPLWKVILSFKARYSKTATDNILDDITPILQFRRNNSEATRNDILQLMLNAEAEE
GAPVNVHSLAIDYDADSASEKNEPAKIGKSKKRRFLTNEEILSNGLVFFIAGFETTGTAM
SFMAYLLAKHPAIQDRLREEVMAVLERDGAFTYDNVFGIKYLDQVISESLRYYSPVVGFT
TRRCARDYTHNGIHIPEGTSVLIPSYHMSHDPTYWEEPEKFDPDRFSSQNKGQVDPMVYQ
PFGQGPRNCIGMRFAQLEMKLTMAKALAKYKFVLDDRHVNEKNLEIGSSFIFAYPQNGIW
LKVQECL
>Unigene0071549
MALRWVSHSLVGTSQLHALLVAVALCMLGFFAYSVVAPAAAKWIKTWLALRPLPGPWDGV
PFWFSAVAYWKKSRECCVKDATVGLFSVICELCETYKGKTFRTYIGMLPIVILQTPEAVE
PLLSSKDNLAKPDIYNFIAAWLGPSNLLTSRGEPWRQKRKVFTPAFHARVLDIYMDVFHS
NSEVFVNQIERFIRSHPNEPFCCFKGLQKCFVDIMARVCMGVELHTQKDERNFFGNCFNR
LSYLTAVRGCRPWLWMQQVYDLTKEGKVFKSTVQKMQMFSYSVLRERKEWLLKNNSVCTD
ISQRKDSAAFDSPSLFLDSLLSYNIRDPSYSIEEVKNDVDSIIFAGTDSTASGVSWTIYL
LGLHPSKLAKVHEELDRVLGRDADGVISSDDLLQLNYLECCLKESLRLYPPFPLFGRKLE
HDMIIDGYTLPEGLTCFVNLYSLHRDPRHFRQPNSFLPERFLSEEFAQRHPYSYVPFSAG
PKNCLGQRFFMQEAKLLLAKVFSKFSVQSTKPVEELKITYEVVLKAKGSLRVWFRERNSS
DAQQSSKKST
>Unigene0006573
VTKFKNFSDRSLSQSLGTEVWKKSIMNLSGEEWKKTRTIFTPALTATRLKTILIKVKTVA
GKMTSRVKEAAAKDELVNFSGLATSSALDITAALNYSIDIDSENERNHPILKSLEDIYIS
AGGWRVVTLFLMPTLSKIVRPDYPPKASTDLFKAFVSLLIEERKSKNQEQDDFLQIFMNA
DYNWEDNAEKKLENAEVRKMTLEEITAQLLVFFVAGVETVSTALSTTTYFLALNPECQDR
AIAEVDKAASEGEITYDSLQEMPYLEACFKEAMRLCTPDSIIMRLCTEETTVAGIHFKPG
MCVDIPLAGIHHDPEYFPEPEKFNPERFMPENKDAVEPFTYMPFGAGQRSCVGMRLGLVQ
AKTTLACLLQHVRFETCPETMIPLKLKPGQLLPFFNGPLLLRAVPR*QTSNSDS
>Unigene0012534
MNWTPLWFYVKISVWTTIVAVLTYGLFRFFKRRQKTFSYFKEIGINGPKPNLLWGNLAEY
HGKGLVKALTEWCDKYGDVFGFYNGDVPTLVIKDLDFLSYIFVKNFQDFTSRGVTMRTDE
EHSFLGQSLLHARGLQWKRTRSCVSYAFTANKFKQMVPYMSHVADIFVQILGEKADAGKE
YPMLRLFQGLTMDYVGRAAFGFDCTFQRELTHPFLKTAQSVLPGVMTGPFHILAHSTTTL
AKYVAPILWLNEKLGSFTYDIFNKHTTKVIELRMKNPEARKPDMLQTMLDVESEEGELPE

APQLLDADAKLYKRMSPEEVGINTTILFIAGFETTATGLSYLAYILAKHQDVQEKVREEV
KSVIERYGKLDYTAVTQGLKYLARVVDETLRMFPPVVTFTTRSAVNDFEYNGIKYKAGTS
ILSPTIQIHMDPRIWPDPEKFDPDRFLPENVAARPTIAYQPFGDGPRNCIGKRLALLEII
YTGARMVEKFKLTLGESQKDRMVMDFHAMVSSPGDGPYIVFHRL
>Unigene0028269
MDVAWSTGGLLVAAVAVVVVAWLQHRRRQMSLFKRYGIPGPEPSSIIFGNWKEFRRDPLE
VTTEWIQKYGKFFGFYVGEIPYVVLADLDMLKHCFVREAHIFRDRMPMILEVKTFQTSLV
GLKGDQWKKVRSVMNPSFSGAKMKVISKLMNDCVDIMVAKIDERLRNKENVVDVSVITQG
LTMDAIAKSILAWEPDSQRNPDDPLVSSLRKTLTEADTLILKAAIAFPPFRDLVPWVFPY
VTYGKIFALICRRLHDVIRARRAEPNVRHLDMLQLMLDEQAKSNICENASDASRVANNGA
ANGFLITDEHIVSNCFISLAAGFETTACTLALILDELARSPQEQDKLYAELSSAFPGNLE
RDAGFDELHSLKRLDMVVSEALRKNPPLVLFTTRICNEETTVMGHTIPAGSRLVAPTWNI
HRDPELWPDPEKFDPERFNPDIVHDRHPASYIPFGIGPRQCMGRKFALLELKTALCKLIL
KYEFAVCPGNEKPVKLKVPLITICPTKNVLLSVRLRNRLA
>Unigene0013587
PRDAVPNRVMEYTEVAKLAAAVALAAFAAWAIIRHRKQGLLRRYGIPGPKPGLFFGNWLE
LKKDRIKVMEEWAGLYGKVYGFYEGDTPKVVIGDMEVIKECFIKKASIFTDRPPLLVDIE
PVRSSLIGLKGDEWKSVRSMLNPSFTSAKIKRMLDTIHDCCDTTVDVLRQSVLHQQATVN
MSAVSQALSMDIITKCALGWQSDCQRKKEDPVVQTIARIFADSGNWINDLCVVAPFLRVI
ASYVFPLLSYGRLFASIEDNLQRLLEWRRKTAANASDIVQLMLEAQKLSTSAYKEESAGL
RTPCNARPQYPFITDRHVVSNCFVFLGAGFDTTASTLASLIYELATHPDEQRRLYDDLVS
AFPHHERLTYEELQTLKRFDAVISECLRLHPPLVLGTSRVCREDTRVATGHLIPSGSHVI
LPTWSVLHNSELWVDPYNFDPDRFIERSVGAAVVSSALSFGIGPRECIGKRLALLELKVA
LTKLIRRFEFSVCSDTHMPLKFKVPLLSLLPEHEIVLRVDLRT
>Unigene0000625
MLQALLLAFVVALLTRFVIQRRRRLSLFRDIGIPGPPPSFLRGNLSEMINKGTLVAYKEW
MEKYGDLVGFYNGAHPFLIVKDPELIKKIQIKDFHNFYNRGMASGFERSHPVFKDNVINA
EGQRWKRIRGLLRPAFTTRNMKQMVGLMDDSIDDFLDVVEFMRAKQGSIEFRELFQRLTA
DVIIRSAFGLKSDLLQKYQTKSTTEPLFRECLNLFQQFRRSWIYFLTACFPEFTPLLRMI
ILWSTRHKKTAVDRIIEEITPIIQFRRDSPESQKGRCDLLQLMLDAELKDEDLANVHSLT
ASADDDDPSKEKPLATEGDSGKKLVLKTSEVQANAFTFFIAGFETTGSSMASLSYLLAKH
QDIQDRLREEVLVVLDRDGEFTYDNVLKIKYLDQVICESLRLMPPLLGFTNRRCVRDYVH
KGVTIPAGTSVIILHRHMGHDPEFWDEPEKFDPERFNPENRGRIDPSVYQPFGQGPRNCV
GMRFAQLEMKLTMAKLLAKYKLFLDDRHIKETELEHESTFNLVYPRNGIWLKVEKI
>Unigene0044491
MDVLWQAGGLRPRMSANVVLLLLLSVLVPLVVFSWKHVATLVRAWRMLRTVPGPPDWLPM
AYVTSAKNAISKSSSTSEEFKLLALQFVLGYAKILETTKIAISRVYIGVWPVVYLNTAEA

IETLLKSSTLLDKALLYGLFNSWLGTGLLTSPKRKWRARRKLLTPAFHFKLLDDFVPVMV
EHARAFANRLRTLSERHQRAPLDVVPLVSCCTLDVICETAMGVSVNAQEDDDSPYVRAVK
VVSGSFLERFVSPWKWVDVFFYSNPTGWNVKRNVEYLHNFTERVIQSRKKEYLENPPVIG
ASEDDAGRPYGGKRLAFLDTLLMSHLKDPTFTEEGIREEVDTFMFEGHDTTAMGISFTLY
LLGLYKQEQEKVHQELDTIFGEDVERAPTLEDLKEMRYLECVIKESHRLYPPVPLVARNA
DEDADILGFRIPRGAMLLAVPYTLHRDPRHFPKPEEFRPERFLPENSAGRHPYAYVPFSA
GPRNCIGQRFALQEEKVVISTILRQFRLHSPDHRDTIRLTWGLVLRPVDGLRVQFLPRK
>Unigene0050868
MVALMGLPEWIILAVTVCVLWYLYASRKRNYWKNQNVPSEPFALIFGPSTKIFYKPIHAI
DAERYKKYGRFFGVFESNKLILFAAEPELVKQVMVKDFPSLPNRRSFALYDPLLDNMMSI
APVEQWRKIRPAASPAFSTGKLRKMNSLIEDCAVATSEHLKKAASNQEDIDVKQFFGHYT
LDVIARCAFATRLDSHSDQTNEFVTKTRQAFSGRITPRLFFFFLFPGIAKMLRLRPFNSD
IFLYFKQICQNIIKGRKDNQSRHEDFLQLMMDAQEGKLSSTTENAAERDNQLFNLGSDAK
PDTSFSSNRTLTEDESMAQCVLFFIAGQDTTSTVISFTLYLLAIHPDVQKKLREEVDECF
RVHGERPSLDVVTKLKYLHCVVSESLRMYPPATRIERSPCEDYVLGDTGVKVPKGELIAI
PVYSMHYDPQYFPDPLKFVPERFSDENLESIQPYTYLPFGAGPRNCIGMRFALQAVKLSV
LYTIRNVEVVRTKKTKVPLEYQSGFSILTAKDLTLGIRKR
>Unigene0024363
TMLHVFVALLVFLASLLFWWVRRKYTFWNGKGIPHLTFWQYMRFCVDIYTKPLSSVLDSY
YKRYGRMYGSYQGATPTLVVSDPDILREIMVSKFKNFSDRTEAQRVSSEVWRKSIMNMSG
DEWKKARSVFTPALTSTRLKTIALKIKAVADRTASRVAEAAAQDKPVNFSKLIEHASLDT
TAALNYSVDLDSENDKDHPLLKCVASLFSDTASWKLIMMFLMPGVYKVLQPSYPPKSCTD
LFKAFVSHLMEERKANNKKEDDFLQVFMDADFEWEVGADGKKEEGERKEMTLDEITAQGI
VFFLAGVESVSTTLVLTAYYLALHPEYQTSVIAEVDKAAGANGDLTYDSLQEMPCLEACI
KEALRLAASESIILRQCTEETTVAGINFKPGMCVDVPSAAIHHDPEYFPEPEKFNPERFL
PENKDSVKPFTFMPFGNGPRNCVGMRLGMLQVKTTLACLLRRVKLEACDETMKPLKFKPR
QLLQVTDGPIILRAVPRDAPSS
>Unigene0047825
EMRCLSRRLLCAVHQAGLRENSSLAAAAAEKQRPVEHDVCPVHGEKLQHLQALRNRPVGA
ASDTARPFHEIPGPKPLPLVGNIWRYLPLIGDMDLTRMHRNAKKLLDRYGTLVREVVVGD
RVVVHVFDPRDMEHVFRHEGRYPARLSHRALLKYRRERPHMYNSGGLFPSNGEEWFRLRH
MFQKPLMQQGAMAAYIDVLQEVTLDVANLIRQTRDSSTNEVEDFLKQLYQWALECTGVLA
LNTRLGCLEQGLSSDSEPARLVEAASETHRIIMVTENGLPFWKVWNTPAYRKLVQSQDFM
ASIVSKYLERHKLEEVQEGRRENGKERTVLEKFLSMPDVDIKDVFAMILDMFLAGIDTTA
YSTTFILYYLAMNRRCQEKLALELGSLLPTKDSKLSMEQLQEAVYLKACIKESLRLSPIA
IGVGRVLPEDIVLSGYNIPAGTVLIMHNQVACRQESNYPEPDVYIPERWLKERTEGSRAH
PFTLLPFGYGPRMCIGKRFAETVMCLLVARIVRNFVLEYKHEDLDCFTRLINVPDKPLKL

TFIDRDL

>Unigene0007332

MLVTVSLLILCTLLLASLLIWRRWHFSYFKRIGIPGPKPNLIWGNLWEYQSMEKYKVIGK

WLEQYGDTFGFYNGDAPFVVTRDINLVEQVFVRNFQNFVDRGFTMMSDQMHPVLKKSIMH

VGGLPWKSLRSAVTYGLSANKLKQMMPHIEEDAGIFIKWLEKCADTGEEVQMLPKLEQLS

MDYVARGSFGIDEHFQGKPDHPLITVAKATLRGTMKGPFHMFAQSTTTFGPLMKPFYWLS

AVFGEYTFENMNQQTAKVIQLRKSDPSLRKPDILQNLLDAEYVEAAEERGDNKVGNGSVR

SRALTTEEIINAASVLFIAGFETTAIALSYIFYALAKHQDVQEKLRREVIDAAGINGPLD

YETVMKKLKYLESVVDEGLRLYPPGLTFVTRRAKEDFEYNGIRFKAGTNFMAAQYQIQLE

PRYFANPMEFNPDRYSPGNEALITKAAHVPFGVGPRNCVGMRLALLKLRYTVARMVQKYR

LELGPSQKGTMELGQYAMVSTPAIGPWIILHTLADERNGS

>Unigene0092031

GRVYGSYQGTVPTLVIGEPDILREVMVTKFKYFADRSLSQTVGSDVWKKSIKNLSGDEWK

KARTIFTPALTTARLKTIVAKVKSISEKMTSRVMDAAAKKKPVDIFELATNTSLDITAAL

NYSVDIDSEKDKDHPLMKSIEAIYMTSSGWKVIMLFLMPKVYKLLQPDYPPKASTDVFKA

FVSHLIEERKAKNKEEDDFLQMFMNADYNWEQNAEKESEGAEVKKMTLDEITAQGITFFV

AGVEGVAIILSAAAYYLALNPDCQDRVIAEVDRALAKGELTYDALQEMQYLDACLKEASR

LCTPDSILMRVCTEETTVAGIRFKPGMCVDIPFAGIHYDPEYFPEPHKFNPERFLPENKD

AVRPFTYMTFGAGPRNCVGMRLGLIQAKTMLACLLQHVKFEACPETMVPLKYKPRQLIIC

FDGPLLLQAVARQRPTSNSSQ

>Unigene0026217

MVSAFITLLAILFAILFWWVRRIFTFWNDKGIAHLTFWQYMRFAYDIYTKPVNEVICRTY

TRYGRVYGSYLGTVPTLVVGEPDILREILVTQFKNFSDRSLSQSVGSDVWKKSILNLSGD

EWKKARTIFTPALTANRLKTIVTKIKPFAGRMTTRVMDAAAKNEPVDLFELANHTSLDVT

AALNYSVDIDSQSDKDHPLMKCIEAIYMAPSGWRVVMLFLMPTVYKALQPDYPPKSSTDV

FKAFVSHLIEERKSKNRQEDDFLQMFMNADYNWENNAEKISDKAEVKRMSLDEITAQGIV

FFVAGVESVTTALTVTIYYLAVNPDRQERAIAEVDKALAKGEITYDVLQEMQYIDACLKE

GIRLCTPDSVSFRVCTEETTVAGIHFKPGMCVDIPLAGIHRDAEYFPEPDKFNPERFLTE

NKDDIKPFTYMPFGAGPRNCVGMRLGLIQAKTILVSLLQHVRFETCPETMIPLKFKPGQL

LPNFDGPLLLQAVPRQHSLNNVS

>Unigene0009181

MLHAILLVLLSVVFLVVGILAKTFTFWKGKGIPYLSFVEYIRTVHENFAGELNKVSLRNY

KRYGRIYGSYQGLIPSLVVADPDVLREICVKDFKSFTNKSDNKVSGNGLWDQMVLHQQNE

EWKHTRSSLSPMFTTAKLKAMVPKMMKTMDRFTKLLLAKLSAGQPTNLCQLLEKSAMDLY

TSLIFDLDIDSHVDTDHPMLKCYSGFLSAPGGWRLLMMTTLPRLFKAFRVEFPNKGDSQY

ALDFTKHLIERRLRENETHDDALQMYMDTKMQQNGKGGVQLSESAMNDISAQCMCLFIAG

SDSIALTVTCAAYSLALHPEIQEAVIKEVDAADEATYEALRSMVLLDAVVSETLRMYSPT

SVLTRICSQSTTIAGVHFTPGMRVEIPAHAMHYDPEFFPEPESFKPERFLSEHKDSINTY
TYLPFGVGPRSCIGMRMGMVQVKYILCRLLQKVTFEPCAETQVPLSFAKGKVLLEPDPPI
QLKIVPRAKR*SVHSSNSP
>Unigene0052776
MELFGLPDWIALAGTVCVLVYLYASWNRNYWKKQNVVSEPFSFPFGATLKLFLRPMCQLD
SERYTRYGKLFGTFEMGKAVLFVGEPELVEQVLVKDFALLPDRRTLTFNDPVLDNMMSMA
PFERWRKIRPSVAQAFSSENVQKMNALIEDCALVTADHIKKAALNEENIDLKKFFGSYAL
DLIARCVFATRVDSHSDAKNEFVRRSRQAPSGRLTPRIFVYFLLPFIARATGLRPLSPSV
LEYFRCLSRNVIKSTQQEDRQNESFLRLFLDHQEKCEKSTSSSSERDQRLFNLGSDMKTN
TSLYSGGKLSEDQAMAQCLLFFIAGQETTARVIAYTLYLLAIHPDVQTKLRKEVDDCFAT
HGDHPDLEAITTLKYLHCVVSESLRMYPPVSRLERVPCCDYTLGKEGVKLKKGDLITVPV
YAMHHDPDYFPDPFSFQPDRFSDKNVASIKPYTYLPFGGGPRNCVALRFALQAVKLSLLH
TIHNVQVIRTEKTRVPLEFKNGFGLLTAKDIIVGVRKRP
>Unigene0025862
MDPGGWSVVAPLLAIVVVAVVSFVLQRRWQHSLFKRYGIPSPQVTSLLFGHWRELRRDTI
KVMNEWIEEYGPVLGIYGGDIPCVLLTDLDAIKECLTKRAHEFRDRVPLVVNLEPLKSSL
LGIKGEEWKMVRSTLNPTFSNAKMRIISRIIDDCTTTTLEILDQEIASGAEDVDISSMAQ
GLTMDCITKSVLGWKSEYQRHREDPFLTSLRETLIGADNFIVNISLALPPIRWFIQLVFP
YASYGRLFTCVTDSVREVIKARRLLEGENREKQVRRVDMLQLMLDAQYKSRAQNDKSGGD
RPSMTDDHVVSNCFVALGGGFETTSLTLALLLHELASNPDEQQRLYEELSSVLEDDVTPE
VLYDKLQSLKRLEMVINEGLRKYPPLVFFTARMCYRDTELAGKVIPAGTRVIVPTWSIHR
NPDLWPDPEKFDPDRFSEGRENDRHPASYIPFGMGPRECIGKKFALLELKMALSKLVRRY
EFSLSSKSATTLTFKVPLISINPVKSIVLRVQRR
>Unigene0009177
MLATVALIVWTVVVLGTTIAIVQLLRWRKKHFSYFKDLGIPGPTPNILWGNLWEYHQKGI
TNAIAEWCEKYGDVYGFYNGDVPTLVVKDLDFLSYVLVKDFGNFMNRGMTMRTDEQHPLL
GQGLLHARDLQWKRTRSVTSHAFTATKFRQIFPHLDDACDRLLDLLAKTGESGEKEVAAY
EHFKPLAMEYISRGSFGIANNFQEATEHPLFRMATQVLPGTMTGPMHMIAQCTTTLQNVV
APFFWLNSKIGSFTYDRFAKLAYKAVDVRRENPLPDGKKDILQILLDAEAEEEEAKAEVR
NGEFKIEKKMSTAEVSVNTAMLLIAGFGTTSVALSFVCYMLANHPDIQEKVREEVKQVRQ
KYGSLDYTAVTQGLKYLSCVVDETLRVLPVVVAFVTRSAREDFEYKGVKYHAGLSIMSPT
MVVQKDPQAWTDPERFDPDRFLPENVAQQHGMAYQPFGQGPRNCIGKRLALIEIIYTVGR
IVERFRLEPGPSQKENLELRFYSMVCEPREGPWLKFKPIPVEEQA
>Unigene0088193
RKYGPIVVEKLPGRYSLVHLFTGTDIRNMYQEEGKEPFRMGATAFKDYRASRPEYYADVG
ILNLQGKQWLKVRMSTQQHTLRVRTTMSYLPSMNKIAEEALDLLDKLMDENGRVEDCFPF
LQRWALESVALASVDARLGSLRHPLDLTLDGPAILEDMRTAFACMQKFGYRFPYFRYIRT

PAWQRFEKAMDDFTVRIFRHIVAAAERMTSEETAHEPTILEHMLREKKLTFGEILTFTSD
FNLAGVDTTSTAAAFLLFHLAKNPRAQSKAREEAIAVLGEDSNSVEPQQLEKLSFIKACL
KESMRLNPSLPGIYRKLDHDVVMSGYVVPAGVPIFVDGYVAGRAEENFWRPEQYLPERWL
KTDQEILPHDGYASLPFSFGPRMCLGRRIAELQICTLVAKILLKYTIEYPRENMEFHGQL
INVPKDPTDFSFRKFRPQA
>Unigene0051715
FYNGAHPFVIVKDPELIKKIQIKDFHNFHCRGMSSGFARSHPINKESMINGQGERWKKMR
NLLTPAFTTSNMKKMVSLMDDSSTEFLNALETLRTKNEAVEIRDLFQRLTADVIIRSAFG
LKSDLLQIERKKSTTESLFQETLISFQQYRRSWLHFFTGCFPEFTPLWRLIISYGSRNSK
TVADKCFDDITAIIQFRRENPEKDRCDLLQLMLNAEAEESNVVNVHSLAASGGAESAPEG
NQPAKVNGGGRKYVLSNTEILANGFSFFVAGFETTGSSMAFMSYLLAKHQDIQDRLREDV
LAVLKRDGAFTYDNVFSIKYLDQVISESLRLYSPVVGFTTRRCARDYVHNGITLPAGTSI
VIPKHYLSYDPNFWEQPDVFDPERFSPENEGHVDPFVHQPFGQGPRNCVGMRFAQLEMKL
TMAKLLAKYKLILDDRHVKEKDL
>Unigene0086134
MKQAARALARSVASEAVSLGTGTASKAKPFHQIPGPKPSLPFIGTSWQYTRWGRYNLYQL
HEASTDKYYRYGDLMKEEYQWRRPVVHSFNPDDFQVIFHSQGRWPVRPPIEFVCKYRTDH
PLKYNSVGLSNALDAEWHKLRMALAPVLLQMRNIAELATWQEEICNDFAEYVRWLRDPNT
LEVGCVQDTLSRLALESIFRLCLDTRLGCLQPSHNEQGDACTVIAVARQLFSAYQELYYG
LPLWKYMCTSSYRKYTEAEDVLYKITLGYIQQYGKHVSGNQSNKSLLRALLSLDGLSEMD
VHLTIMDFIVGGIFTTSIALCFLLHHLASNRDVQEKLYAELKSGDISNCSYLRACIKESF
RLSPTVPGVMRILPEEVVLSGYRVPAGIPVFANSLVTCRLEKYFPQPEEFQPERWLGESR
GLIHPFSLLPFGHGARMCVGRRFAEMELMTAAAKMVQNFIIEPCTQHINTSYVFVVVPSH
PVRLRFLDRN
>Unigene0052618
MKHLTRPLGAWRNTAVFARPYSQTVAASVEDAKTTSVKPKPFSEIPRVPSLPLVGSSWMY
WRVVGKYHPDRRHLAAADMYRKYGPVVAERLPGRYSLVHLFNADDFRTLYQEEGKMPFRM
GATAFKKYRDSRPQYYANVGILNLQGQEWYNVRSKTQPYTLRPRTIMSYVPGMDLIAEDA
IRLIEKTRDDKKEVDDCYTILYRWALESVMLASADTRIGCLADPLPPASDGAAFLQDMND
VFGCLQIFGYRFPYFRYFRTPTWRKFERSMDAFTLRLFKHIQEAAHRLQTRQTDQEYTIL
EHLLVEKKLGFSEILSFMSDFIMGGADTTSSSATFCVYNLARNPEAQERARQEVLSVVGE
NCRAVEPHHINNLPYVKACLKESLRLNPVLSGVFRKLDHDVVMSGYTIPAGTPVFTENYV
ASQLEQNFTKAHAFLPERWLKSEEQRDDWTLHPYASLPFSFGPRMCLGRRMAELEVWILL
VKLLSKYKIEYHYEDIGFVGKLANAPDKPARFRFIELKPES
>Unigene0091775
MSALLEKLALAWDWRWITTALVFIVSYFVGRFYHLVSKYPRGPFPLPLVGNLLTLRKVNL
PTKATEWSKVYGEVFTLWMSHRPMVFLNSYDVIREAFLDRRHEFAGRFPTKMGALQTQGH

HDIMFEDYNPCWKALRKVALLAVRKYAVSESLEKLSTEVVDYYVDSLKEGPQIIESREPF
LKILFTLIGVSVYGTTVEEARKEVDRIEEIDRKFFEIAPDGLPSDIAPWLGILYRRREKA
IERVFHDSFEIFNELFSAAEATYEPGKTENFTHAMLSAREDAIREEKGDAEYLTKGNMVQ
VVVNIFGAGTDTSAAELQWLFLMMAKEPWIQEKIQKEIDDNIGNTPPVYKDREKLPFTVA
CLMETLRFFPVAPFGIPHNTTTNTKLGKLDIPKDTGVLFNAYGVNHDPKLWDEPDTFRPE
RFLDPSTGKLRREPLPLVTFGMGPRTCPGEKLAHVDMFYILVRLMQRLSVSAGDKPPVVD
IRSLSNIFLIPAEQNITLTRRN
>Unigene0023990
MSILLEKLVSNAGDWRWITTALVFALSYFLGRFYHRVSKYPKGPFPLPLVGNLLALRNVK
DLHSKATELAKTYGDVFTLWMAHKPMVILNGQAAIREAFLDRRHDFAGRFQTKMGELQTQ
GNHDILFEDYNPRWKALRKVALLAVRKYAVSQSLEKLCADVVDAYVDSLPHGPQTIDSRT
PFFYLLYNVIGVSVYGTNLKEEDTEIRRLEAIDSEFVEVAPNGLPSDIAPWLAFLYHGRE
KKIKEMFVEFREIVNRLFTKAEATYVAGKNENFTHAMLAAREEAIREEKGDAEYLTKGNM
VQVVLNIFGAATDTSAGELQWLFLMMAKEPKIQEKIQKEIEDTIGSTPPVYKDRARLPYT
VACLLETLRFRPIAPLGLPHKASTEAKVGDVVIPKDTGLLYNVYGVNHDPKMWEKPEEFR
PERFLDSATGELRQDAGPLITFGMGPRTCPGEKLAHVDMFYILVRLMQRLSCSAPGKPSD
VNLNGSGSSIFLLPEKQNIVLTRRN
>Unigene0085945
PLPAALRNVTDLHAKSTEWSKTYGDVFTLWMGHRPMVMLNSYPVIREALVERRHDFAGRF
PTRSGALQNQGNHDILFEDYNPCLKALRKVALSAVRKYAASESLAKLCADIVDAFANSLK
EGPQIMDLRKPIFSMLCKIIGVSIYGTRLHEQADDIERLEDTNRRFYKISPNGLPSDIAP
FLAVLYLKKEKSMAAVLREIGEILNKFFTKAEATYSSGKTENFTHALLAAREEAIKEDKA
DAQYLTKANMILVTMNLFNAGSDTSTGTLQWLLLRMVKEPSIQARIQKEIEDNIGSVPPA
YGDREKLPFTVACLLETLRMHPAAPLGIPHNTTTDTRVGNWDLPKDTALLYNIYGVHHDP
KHWEKPEEFRPERFLDPVTGKVRTDAGPLIAFGQGARICPGEKLAHMDMFYVIVRLMQRV
TCSVPSGPSDISLSGNGSSLFLHPAQQNVVFTRRN
>Unigene0059012
MTLLAVIVAWDWRLLTTSLVFLATYYVVRFYHKVSQYPKGPFPLPVVGNLLTLAKEKELH
KKAIAWSKYYGDPFTLWMGSRPMIVLNGYEVIKEGFVERRHDFAGRFATKLGDIQRHDDH
DVVFEDYNATWKALRKVAVTAVRKYAVSESLEKLCAEVVDAYVDSLGDEPVTVDARDPVM
FIIINVLSVSAFSAKFDAKSADLARIMDINRTFTKLAPNGLPSDIAPWLGILYRAREKKC
EDLFAEMRSIVNRMYAGAKKTYEPGKVLNFTHAMLSARDEALEQDKSDAEFLTEGNMIQV
LIDIFGAGTDTSIGELQWLLLKISREPSIQERIKKEIDENIGQSPPTMKDRERLPYTAAC
IMETLRFYPIAPFGLPHKTSNNSEIGGFPIPKDTRVMYNAYSVNHDPKLWTDHDVFRPER
FLDPVTGKLTSKDRLPPLLSFGLGPRSCPGEKLAQADMFYVLVRLMQRLSVAAPDGAVGR
EALPMGYSFFLVADRQDVILTKNR
>Unigene0001612

RYPRGPFPVPLFGNLLALRKIENLHIKAIEWSKTYGDVFTLWIAHNPMVILNSHVVIRET
LAKQKLLFAGRSSTKMRDIQTQGNHDIMFEDCTPYWKALRKVAALAVRRYALTRTAEMLC
VDVVDAYVDTLAPGPHVVDSRKLFFSMLYKLTGASVYGESLNGEQRDIRRLEEIDHEYYG
IVPNGLPSDIAPFLGLLYCKREKRVEVIYKEYREIVYRLFAKAEESYRPDSKENIVHGLL
TARQEAIQEKESDAVYLTKENLVQVVMNLFGGATDTSASTLQWIFLRLVKHPEIQKRIQE
EIQDNIGSRPPRYEDREKLPFTMACMLETFRCHPFTPVGMPHRTTADAKVGTTVIPKDTG
VLYNVYRANRDPNLWEEPEEFRPNRFLDHATGRLCQDAGLTFTFGAGARACPGMKLAHVD
IFCILVRFMQKVSCTAVGNTSSVAIKSIRSSLFSIPAQRDIIFCKRN
>Unigene0001298
MLEVAVRECAITALVVALGATLVHYLWFLLRRALKRDLPPGPRGLPLLGYLPFMTMDGHR
EIEALRRKYGNVFGLHLGFRYVVFLCDFDSIKEALSKDALLDRAEEFPLNVHEKSQSIIV
SNGLQWKEQRSFTLRAMKALTPTLEAHVHEEASNVVRQLASSEGKPVAVVSLLTSSTSNV
VTALVYGRRFEYGSSERVQLDELADIIPTLSAQVLPINFFPWLRRVISFLNVGSCGRLRS
AMIRRDRLSGSFVGHHEKTYQEGTVRDYVDSFLCEMKRQRPGKKSFTRDVLTSNAASFFG
AGSETLRSTIEWLLIMCVANPESQERIRSEIDSVLGEDGAGPRILWEHRSRMPYTQAFIW
ETARCETINPFGFMRRASEDVKVSGYVIPRGSIVIPSLSSVLCDASFWKDPEVFRPERFL
VDDGTRAVKPERLIAFSYGKRTCPGETIANVATFLFLAAILQHFTVEVPADSPALALDGV
LGLSVRPRSQKLVFRARAVRC
>Unigene0071823
ESSWSVVGAYLALVYVAYLTLRLLIITTTKHEKKLPPGPKGLPILGYLPFIQKPYHVAFN
ELSAKYGPIIRLWLGCRDVVVLNDLQSIRAGLSNPDVLYRPNDFIFSYLGVKGIASLNGE
PWQANRRYCFHVLRNLGFAKKSMEAHIQEEIQCFVNHLASAEGEPMAFAQKLAASVANNI
SALVFGQRYELEDPRCHYIEGLVTKFLRYGSILSVLDFLPTVRAVCSYIPNTRMFIAKDV
MKKMKEAIRTEVKNRETCMEDYFERDFIDGYMRKMDENKDTNSHFSLRHLEGNAINLYGA
ATNTVRTAILWNLYIAASDPDGMQSRVQREIDSVIGREKPVVWEDRHRLPYTMASVLETL
RWRTTAPIGIHRVAMSDTKVCGYDISAGTFVIANLWSVHNDPAYWCNPREYDPTRFLKSD
GTELAEKPPAFLPFSVGRRACPGETLGLMEVFLYVATLLQQFRVLPEDGIAISLDPKNVF
ISVVNDAQKLRFLRR*ASRLS
>Unigene0006635
SMLIILTLILVCIGWIASHFFNEWLLRRRCPPGSRLPPMPPTTSIRGHVELLAHDFHRKK
CLDWARDYGPVIRLKVDFINVVILNDLKSIKSFCNTRELLWRSSCFVRYRDSCKGLGVMN
GETWSANRKFCMSLLRGSGFARRAMENDMQEEIRRFIQSIRKTDGRPYDVGSYVVLCAFN
NVAHFFYGAQLSRDHTTIHEFHQLLQQLGRALFGPQNQFLPWMVRRFLTWLPFTRNHRIA
SGVAKMDAINSKQIEKYKARVSGDESMDFILGYSKKIEDSKYESTPLFTDHYLVGNLNSF
LLGGTFTTASLMMWHLLNFAKNPETVQAKVQHEIDNVIGHNRQPSWEDRKRMPYTLACIW
EMQRWKTIAPLGVSRECADDVVVGDLFVPKGTVVIPNIWAVHNDPNSWHEPAMFKPERYL
RKDGTLLPRKPEHLIPFSVGRRSCPAESVGTMEIFLMITFLLQKYRILPEEPIDCDFDSL

DFEMSSFKSTRLCFLPR
>Unigene0061557
LTILLTSVVTLLLWLLFKFIDAWIPEWRAPSGSKLPPMPPASSLLGHVELLSKGFHRTKC
LEWVKEYGPVIRLRIGLKHVVIVNEYNAIKKFWNTKEILQRWSTFTGYTDTYKGLNEMNG
EVWSANRRFCLSMLRDLGFAKTAMEGLMMDEFRRIADVIADTNGKPIDVRHYVTPCAFNN
AVSFFYGEQLTHDSTTTRSLHQLLRQIGRAMLENTAYNFIPGRLCRLLSWMPFTTSHRIA
LLMGKLNAVTAEQIERYKAAKYEEDPKGFILGYVKKIAKYRSTPQPLFTDHYLTGNINSF
LMGATFGTTTVMMWHMLNFAKNPDTVQARVQREIDNVIGQERLPTWEDRKQMVYTMACVW
ELHRWSTNMPLGLARECGKDAVVGGFFIPKGTILFANFWAVHNDPALWQHPQKFMPERYL
REDGGPASGKPEHLLSFSVGRRDCPGQTFAIMEIFLMITFLLQRYHIMPEHPIELDLKDP
QMELPRAINVKLRFIPRQCTKH
>Unigene0017155
CGFESIKAALSHKAILHRPKEFTLNSSSPYDSLITLNGPCWDQQRRFFLKMFKELGAGTP
AMDKVIQEELSYLLETLKTRNGQPVTTQDILTPSTSNILMKLLFSRRYGHDHPKRAHIDN
IISSVLMYSTPLLPVNFLPWLRSALSHLGLGTCGKLRKALQRRNQFSECAVSENAKSYMD
GVVRNFTDGFLAEMNRRGGERSETFTWNILVGSVSSHIGGGTVTMCSALEWTLLMSAAYT
QLQRKVRAETNAVMKQREPGSTITWNDRREMPYTEAFIWETIRCRPVNPLSIFRCAAEDV
NLCGYAIPRGTIVIPSFWSLFNDSTLWKNPLEFRPERFLTGDGKTAQKPKEFIPFSYGRR
SCPAESLATMTLFIYFANILHHFTAEIPDSAARMDDGFLGLTFHPNARKLVFKPRAH
>Unigene0051602
GLGQLNGEAWSANRKFCMSMLRDLGFAKTAMEGRMMREFRRIADGLGDTKGKPVDLTQYV
TPCAFNNAVSFFYGEQLTHDNSTTRKLHGLMGRMGRTLFHGQAQMLIPWKLRRVLSWLPF
TTSHRIADLLAQLYDVSAKQIELYKTSKPEDDTRDFIHRYSNKIGESRDEQMPLFTDDYL
VGNVNTFLMAGTFSTTNTMTWHMLNFAKNPDTIQVRVQREIDDVVGEERQPTWEDRKMMP
YTLACVWEMDRWKTAAPLGVARECAEDVVVGDIFIPKGTILLPNIWAAHNDPALWEEPEK
FIPERYLKEDGKLVSHKPEHLIPFSVGRRDCPGQTFAAMEIFLLVTFLLQKYRILPEHPI
DFDLDCPEIDLPDARNVKLRFIPRKSGK
>Unigene0040778
SLELRMIFMILLTSAVALLLWLYFHLLGDRRLQRRMPPGSRLPPMPPASSILGHVEILRR
DFHHKKCIEWAKQYGPVIRLKVNLLDIVVLNDYESIKKSCNTKEILWRWPSAVGYHERQK
GLNEMNGETWSANRRFCMSMLRDLGFAKTAMEGRMMEEFHRVTETIGDTNGKPVDVTRYV
APCAFNNMVSFFYGDQLKHDSSTVRKLHSWIGKMGSALFEGRAQHYLPWKLRRLLVWIPF
TRSRQIADLMAELDAVSKEQIGLYKTKKIDENKDFLLGYIKKIEESTKRADNLFTDGYLL
GNLNAFMIGGTFTTTITMTWHLLNFAGNPDTIQARVQREIDDVVGDERLPSWEDRNHMPY
TLACIWEMNRWKTVAPLGVSRACGDDFVVGDFFIPKGTIVLPNLWAVHYDPALWKDPEKF
MPERYLNEDGTLVSHKPEHLIPFSVGPRDCPGRTFALMEVFLLVTFLLQKYTIMPESPSE
LNLDSPDIVLSHATNLKVRFLPRKSAK

>Unigene0054607
LLNVFTWETAWLVSALCLVLMTVLRILSSSAKRHRGDLGRLPPGPKGVPILGYLPFMRHT
FHASFHELSKQYGPIVRLRLGCKDVVALNDLASIREALANPDLLYRPDDFIFRFLGVRGL
LTLNGEPWQVNRRYTFHVLRNHGFAKKSMEEHIQEEVQDLTGHLSTTNCRPTLIGHTVAA
SVANNISGLVFGRRYSRNDPEGRFIENILTGFLHVSSFLSIVDFLPAIRAVAAYIPCTKT
YLTGKFFRDFIELVRMEVGKRKENMEMHLDRDFIDGYLRKIRENEVTASCYSMRYLEGTA
VNLYGASTNTVRSSILWNLYIAANDPDGHQAQLQREIDRVVGRERPPEWQDRNRMPFTMA
SILETLRWRTLAPISIHRAAGRDTVIDGYHIPAGTLILPNLWSVHNDPEHWLNPSKYDPT
RFLNADGTKLKERPQAFIPFSLGRRACPGESLALMEIFLYLTTVLQKFTVLPEEGTTLSL
DIQHVLISAPDDTQALRFIPR
>Unigene0037060
MPPTSSIIGHLELKDPKFHSRKALEWAKEYGPVFRLRIFFRNVVVVSDIEYIKKFCLDNQ
TLYRPDVLNLGRSYYQGITTMNGKEWKDNKRVCMSALRDLGFAKPTVETKMMDQCRKVGE
AVEKAEGKPLHLGWQFIEWSANNIAYFFMGPWKEGSSDMIDKMMDILQRSFVLLKSAGIY
EYTPGILRTFLKLIPSTIDYKIDKIFKELDNFIIEQVKNSAPIEGEASFIEEYTKMIPES
STNESGSYQYRYLVGNIRAFILGGVFGPSVSMHLILLFFAARPNDIQRRVQNEIDAVIGN
GRTPTWEDRKRMPFTLASIWEMERWKSDSFGLPRETETDLVVDGFFVPKGTVVLFNLWAV
NRDPSLWKDPHHYNPSRFLLEDGSLMPHKPTCYVPFSFGKRSCPGDVFAFMETFLMVTFL
LQKYDVHLDQPLPCDLDDPTTCYEKLQTTKLRFLRRSDGANRCSNVGN
>Unigene0061558
MISIIFLASAAASFLSSLLWTLYVFIDTWLWRWRSPHGSKLPPMPPASSIQGHVELLRND
FHRKKCAEWTKEFGPVIRLKIYFQNVVILNDNNSIKKFFNAKEVLWRWPTFVGYSDFYKG
LAQLNGELWSANRKFCLSMLRDLGFAKTAMEGRMMEEFRRVAGSIGDTNGEPVDLCRYVT
PCAFNNAVAFFYGDQLRKDYSTASKLHRLMGQLGATLFQGRVQPFLPSKLRQVLARFPFT
RSHRIAELLAELDAISAEQIKLYKASMSADDTKDFIHGYIKKIEESRGHPHPLFLDRYLV
GNINNFLMAATFTTTLTMTWHMLNFAKNPDTVQARVQREIEEVVGQERLPTWEDRKRMPY
TLACVWEMDRWKTATPLGVARECAEDIVADNVFIPKGTIVLPNIWAVHNDPSLWKDPGKF
IPERYLTGDGQLVPQKPEYLIPFSIGRRDCPGQTFAAMEIFLLVTFLLQKYRILPERPSE
IDLDSPDFALSHATKVKLRFLPRKSAKC
>Unigene0026622
LVWRWLDRRRLPLGTKHLPAPPTSSIIGHLELQQPKFHLEKALEWAKEYGPVFRLRMYFR
NIVVVNDFESIKKFSAHSETLHRPEIMNFGPDNNKGLLTMNGKAWQDNRRFCMGVLRDMG
FGKTTVESRLLREYRHLDEEIEKAAGEPVDLSIPLMECAASNVASFFMPRVQSDSRVRAE
LMIILKPVFLFYQREVLYDFWPPFYECLSRLLPFTCTGRAKRIIKDFDNFIINEISNDVQ
LNGQRNFVQEYLNMTSDPKLNDNGSFHHRHLVGNIKMFIIGGIDGPSVSMQLCLVIFAAR
PTDVQMRVQREIDAVIAQGRKPTWEERKRMPFTLACVWELERWKRTEPFGLPRGTDADMV
VDNYFVPKGSVVLFNLWAVNRDPSLWKDPHRFDPGRFLKEDGSVIPEKPNYHVAFSYGTR

SCPGETFAIMETFLLITYVLQRYTVELGEKLTCDFDDPAINLDKLKSFQLRFTRRPKS

>Unigene0070874

ISRLQLGVKSVVVLNDVTSVREGLSNPDVLYRPHDFIFSYLGVKGIAALNGVAWQVNRRY

CFHVLRNLGFAKKSMEEHIHEELGCFLELLTSSKGQPLQIAHRLAASVANNISALVFGRR

YDLDDPNGRTFEGLLSTFLRYANFLCVMDFLPFLRTLAYCIPNSKLRIMNYVMKELTRHV

RVAVKDHEENMDQYAERDFIDGYLRKMQENKGSNSHYTMKYLEGNAINFFGPATNPVRTM

ILWNLYVAASDPDGQQTRIQREIDVAVGRHRLPEWKDRLNMPYAMASILETLRWRTSSPF

SLHRVAGRDTVIGGCHIPAGTLILPNMWSLDHDATIWRNPSEFDPTRFLSADGKEVDEKQ

LAFIPFSVGRRACPGETLALMEIFLYVTTILQKFVVLPEEGKNISLEIEPALLTVADDMQ

GLRFFPR

>Unigene0014181

MLAWSRIYGSVVRVKAGSSEIVSLNDMDSIRTFLNHKDFLYRSKNWVVREIVELGFSGYG

GAPWDKNRRFSFKILRDLGFGRSAMGDIISEGCRVLISKIAESQGQPIDLFDLILASVSS

NFEVLLLGYRYPLQHREHQLLCKALRDEFRTTRDGSVLSFYPSLAKAVAKCLPFSETATL

FSAMKWIETFSRTHLSHRMTTLPDKEDRDFIDAYIRKTKEYDKKTEPAYGLSCLVGNIVS

FLVAGAVSASASLMREFLLVAANPDTIQARIQSEIDAVVGSERQPAWEDRASTPYTMGFI

WEAHRRYTIAPLGLPRRAYQDVIIGDQFIPKDATVVPNIWAVHNNPAIWKDPANFDPSRF

LKSDGTLSKKKLKKIIPFSVGRRMCPGEIFASVEIYIYLTSILQKFHVLPVEGDTVDVDV

KYGEIMEPGRQ

>Unigene0003035

MEWPDLITAALFLLVFYVFWYSQIGKRRIPPGSRLPPSPPAIPLIGHVEYHRPHFCATTA

IEWARIYGSTYRIKTGLTEMVIVNDFDAIEKLFSKDELLCRSKNWRLYGLSVGYPMSLEG

EEWVQNRRFCLQVLRDIGFAKPAIQNDILDECHRLIARLSESLGQPIAVEDLMLPSVSNN

AVATVFGESYPFDHPKRKWLDDTLRKLAVAVNTTRRITPLGFLRGPFLNTLAFPGRREIE

THFRALGDFTRKEIYARKHAINKPSSKTLIDAYLSKMAEEEHDSESKMSVENLMSTAIGF

FQAGSAGVPTYMHWHMLNFALRADTLQAQVQAEIDSVVGPLRRPTWDDRRNMPLTMAVVW

EMLRWKAITPLSAPRRAEVDFTCDGFFYPAGITVMANYWAVHNDPSVWPSPEKFDPSRFL

NDDGSATCERPKHLISFSLGKRKCPAETQAIMVIFLYIASILQHFRVLPEEGVTIDIGDA

NDRLPTKNKYRLRFVPRRK

>Unigene0011321

GEAWRLNREFSMRALAKLGFGTELMHRYIQEEASHLCDFLASKQGCPVSSFSITHKCHIN

TMCRFLLGYRFDLDDPRFAPLQTALSGFRLQSAAAPVEHRAAWLRRIIVDRLWPSSVSAS

RQRLLTSLNTAVRDLIAQNEDSNGRKRQTSYIDIYKEEMREAEKKNNPHFTVDYLAGNMC

DIVLGSATSTNVYLHWNLLNLASRVDDLQAELQREIDSVVGRRRLPCWDDHRHMPLTMAT

VWEMFRWKVAAPFNLPRGVGEDVNFKTYTLLKDTVVVPNLMAVHRSRKLWKDPDTFNPSR

FLRADGKTQTTRPEGLLTFSVGKRMCPGEAMALVQIFLFLTTLLHRFHVLPVEGEIYDIA

PFGPSLELTDTKLRFVSRA

>Unigene0007647

AAIISAYGALQSGSVGDICGMTIYISALVAVVLGSAFIWYSRRKNRDPNRKRHPPGPRGI

PLLGNLEFNKPKFYHTKGMDWVRQYGPIYRIKMMSVNVVVINDYDLLKDTFSRPEILHRP

AAWLLKETTAGLAVLGGHQWRENRKLVTQAFVELGYGKQTMSDIVQAEGQHLMEAIGKFR

GAPMLPRDLMLRSACNNVVTFLLGRRLDLDDPRRKDMDDHLEGFFLGSAASSIDCRPQWL

KKLERWLRPRSPRVRIENMANELEAMSKREVYRAMAMDKSRRNQAVVDVYKAKLEDLDKE

DDVFSEDRMVGNVTDYLLGATAVVALFLQSHVLNFAARADSLQKEVRQEIDRVVGRDRLP

TWADHVHMPLTMATIWEMYRWKACTPFGIPRGVAEDTVIGGYHVPKGTVLLPNFWAIHQS

TELWKEPEKFDPTRFIGPDGSATSTRPAHIITFSLGKRMCPGESLATAEVFLYLTMLLQK

FRILPEEGTTIHIESHQPLFEHAATKIRFVPRSD

>Unigene0007755

MWLLLVAASGVALLVFRWLLDRSRKPRSGDGDLPPGPQGLPLVGYSLGLKPLEQLDALAD

EYGPVFMFRLLGKDYVHLGSYSAIREAYVKLGDCFVGRPRDSTAMGYLLDHQGISNSEGH

EWTEHRRFVLHTLRDFCFGKLSVLDRVQDAAHRLVGRVVAQGGLPFDPEPLIFEAVVATM

AGLLFDVEYEEGANNALSSSSSAAAAATAPGAGDMRYESEQQQQTDEGAVPADDATKAED

GRVVVPPVSVEAEDEAYLARLVRLVAKATPLLNNDVLPQLWTCASPVELSPGFSELQRLK

RELDEFLERMIADHEPSLDESRLRDYMDVYLDERRRAMEEGTLHKSTFTINRLKTICVDL

LVSGTASSVAQLCWTLKLLARHPECQRRCQDEMDAVLSSADKLGSVFSKDNLPFTEACLL

ESARFASVHVVAAPRTNVEEASVCGFRIPAGSRVLANLWLAHRDTSFWREPHMFDPTRFL

AEDGRPERKDAFLPFGLGKRICIGESLAKTQMFVFVTELLKRFTFTVPEEFATQDLNFRP

PQGTLRFAEPFHLVATPKILSTTL

>Unigene0028659

HGTAEQTRLSNSVVTELGYLLSELSRCSGTAIKPRQALLVTTANIFYTFFCSERFSPDDP

KFLRIVGLYNEVFHQLFQGFAIDFMPWLKVVQGKQLCLLREKSMEIYRFTLAIMDRRENV

IADGIGAQVNHVRDLMDVLLLSLNDPSTEGQLERVDVAVAIEDLIGGHSVIANLWVWCLY

ILSDYPDVQRKIREETRAISSSEERLPSLLDRGRMCYTEATLYEIIRVVNSPIIPHVCSN

DTTVQGFHVRRGTVVMFNTNDINYSSDLWEEPWDFKPERFLSSDGQYVLKPGHFFPFGTG

KRSCMGDGLVRATLILGMAALLGRFELSLAPGQEPARFADFRSKVIFDRDPEIIFTEVSS

RTQLPQ

>Unigene0057049

MIELSTASIVVSVAACLCYAVARWFISERKKCFDAFEGTGIPGPPHRSLINGNTREFLNT

NQIKCLGRWLDEYGDVFGFYLGDVPFVVVKDPEMIKEIFAKEFNVFSYRGHLLRIHEMER

PLERNLVLVGGRQWKTARSCMQQFFTPAKLKVVMPRLHEAQSEFLTILGECADTGGEVDI

GCRCERLTFDVISKSAFNLDTQCQRNPQNPAFQMALRCFPGIMSGFMYHLALNLYHWPRV

IKILHKFFGHFFTNPLVALTKYAAELIKFRHENPQIDVPDMAQLLLDDALGKSNSETKKS

EMRTMTPAPLSEEKLYELGTNCMDVFLGGYDTTRLALTYWFYLMGKHPDVQERMRSEVLE

AFKKEGDVLSIQTLTGLPYTNQVLSETLRMYPPIIAFTTRCAGEDYQYGKYLIKKGTSVM

VPTYHMHHDPQFWTDPEKFDPDRFSPENKHLIKQATYQPFGLGIRVCIGQRLALVELASV
TSQVLRRFRITHGPSQKPDLGLLTYAFLMAPNDTVWIKLHKL
>Unigene0039246
MVSVTAEWNSALVVVAVVVVSLVSLWRWRRNKFNFFKERGIPGPEPSLISGNFFQLWNRD
TIKVLDEWSNKYGDIYGMFNGDAPFLMVKDLELLRRVFIKDFAMFVDRGDVWALMNARPE
QRNSVSFAKSDRWKFIRRFISMAFTSAKLRPMVASMNKSVDNCLDLLETRCREAPDGQAN
AYPLLGCLAFELVAETACGLYLDVQNKPNDQYFSSAKTYVLNVVESFYQRAGQFLTGVRS
LVALTCVLERHFGDEPLVALCHKAEPIVAFREKDPSLARPDMLQSLLEAKVPEELLVRSE
FRERTNDEGEFLMPVKAIACNAASILTAGFETVSANSSSCVFCLARYPEIQEKVRQEVNA
AYEKHGGFTYDAISDLPYTTQVIFETLRLYSPVVAFTSRQAACDYRYKEMLLPKGINIMA
CTPQIHRDPRHWNRPEEFDPDRFSPEQKASRDPLAFQPYGIGPRNCVGMKLAQLEMTLIV
AKLVHRFRLRLGSKHENGELKMHTHSIIASPKNGVWLTVEKIR
>Unigene0089040
MNGIIQDCARVATQYLLKVAESGNDIKPKVVFGHYTLDVITRCVFSARIPSHSKEADEFV
AKSMAAFNLIKPTLPVILFFYAWILFPCIQLLLKIRIFKPDTFLFLKDFCLNVIQERKLA
AVTHHDFLQLMIDAQNFGAQSGADRPQDTEEKLYNLGNEEAATAPASVKALTEEEALSQC
VAFFIAGQDATSSVLAYTFYLLALHPEVQQRLQEEVDRCFEENGEEPSLDDIYKLKYLNC
VISESLRLYPPAVRYERTACQDLALGDTGIKLSKGCVVGIPVYAMHHSPEYFPDPEKFDP
DRFSDENIGSVRPYSFLPFGAGPRNCIGMRFALQAMKTCLLHVVHSVELVRTENTKVPLK
MVISFGLMTAEDITIGVRKRATS
>Unigene0027157
MDAVAFQVLPNVAALLASFALYCAAAAVSAFACLSGLLYYIKCKQRETNVYMEKFPGRTE
PIPMLTTWMIHRTLSKEAHRLDIATHLFQVSVGFSRIHQKQGMLRFYDATHPILVLFRAD
HIEEVLTSNIILRKGHEYDLLNPWLGTGLLTSSGTKWRSRRKLLTPAFHFRILEDFLPAL
NDQSQILVRKLGQLGKDRSCDIVPAITLCALDIICETIMGYTINAQSNEQSEYVQAIQVL
GHSFTRRLESPIYWIDAIFRLSKDGREFRRKISELHRFTMKVIRERKGELLSCPELQEPV
DETTSESDAYGLKGKTRKPFLDLLLREHIKDPENFTEEGVREEVDTFMFEGHDTTAMGMS
WALYLIGLYPEHQELIHQELDDIFGSNQTRPVTSDDLKQMKYLECCLKESQRLYPSVPFI
TRKCEQDFMIAGRKLPKGADIQMSIYNLHRDPKVFPKPEEFIPDRFFPENVKGRHVFAFV
PFSAGPRNCIGQRFAMMEEKVVIANILRNYKLVSLHHRDKIHVKAELVLRPKSGLHVKFI
PRKCAKVQ
>Unigene0091089
MSAALSSVVSLTFFAPSRFDVALLSSLPWVTALLSCALMATNCRRWFYTWSTLRPIPGPG
TDWLPPLFLLSVYWQYRNHLTKSATSVVFRVVRKVCKMYEGKTFKVYIGASPIVVLHTPE
AAEVLLSAKENTGKPNSYAFLKSWLGPKNLLTSKGDPWKSKAKLFKHAFNTDHLESCMAV
FNESGEVLEKRIETMASESPDQPIVCYNNIQNCVLDIIGRASFGIHLGLQDGNRKEYARW
FNYLTYLLTVRYFRPWLWIPAFYNATKEGKFWKRTVDNIGKLHLSVIERRKAAIMKRLAD

ESNEDDLDDELSFPAAVDAGIKRHISAPSSYPLDELEKDATSVTFAAADSTSAAMSWTLY
LLGLNPDKQAKLQRELDDVLGRGAQQEITTSDLKQLPYLECCIKESLRLCPPFPLIGREL
DDELIIDGYTVPAGTTCMINIHSLHRNKEQFDDPESYIPERFLPENCKNMHPFSFIPFSS
GVRVCLGQKFVMAEAKVLLAKLLSKFTVESTLPIEDVKQAYEVVLKARGGLRVWFRKRSD
>Unigene0049737
MSAGGEATLFPMTLTGFLPFSSHRVLWSLTLLLSCIVPVAVHIARRRRIAELISKIPGPT
AAHPILGNLDVLYELKKYRHLLAPHILLLQTMCGLAQIHDKDRIFRFWLGFRPVVSFFKA
ETVEVILSSNTVLDKSFDYTLLHPWLGTGLLTSSGNKWRRRRKMLTPAFHFRILEDFIPV
FNEQAVIFVKNLKEQQNKKYIDIVPLVTLCTLDIICETAMGVKVDAQLNSNSHYVRSLYE
VGETFMARVMRPWLWPNYVFYMSSFGRKFKDNLAQLHNFTRKVIRERKAELLEQKVIDGL
TIGEPVIGQKRRQAFLDLLLSHHIQDSSLTEEDIREEVDTFMFEGHDTTAMGISWAMYLI
GLYTDVQQKIHEELDAIFGEDRERAITPDDLKEMKYLECALKESQRLFPSVPFIGRELME
DVVVNGYTVPRGTTCFLFTFMLHRDKEIFPNPEVFDPDRFRPENCVGRHPFAYVPFSAGP
RNCIGQKFALMEEKVVLCSVLRNFCIQSVDFRDKIHLVAELVTRSKHGLKIRLRPR
>Unigene0061866
WRRSKFQYFKDMGVPGPRPSLIFGNLLEIKRRGGAAMFAEWIKTYGNVVGFYNGAIPFLL
VNDLELLKRVEIEDFHNFAERGNVIEVQSVPDIRQKLIVTAPVNRWREMRAVLSPAFTTK
KLSQIFVIMDGCSDTMIELLRDKVEEGEAVEVSKVFRRATMDTMFKVGYGVDLNVQRSTP
GGPLDQMGDGAGALLRRVPLQGISFLSNCFPELHHIWFLLSWLTSRLVVPYFTLTTKLIQ
PVMSERRSKAMREKADVLQLLLNKESTGQLFRNDAHGFDGKTRLALTREEVIANSAFYLI
AGLEATPNTMGLTLHLLALHPEIQDRLQAEISEFLHRDGKFTYKNVMEMPYMDMVLNESM
RFYTGVVGFVTRLAARDFEYKDVKIPKGLSVMVPVTYLHHDPEVWHEPEKFDPERFSPGN
KPFIHPVSFQPFGKGPRECLGKNFALLEMKLMLSKFLANFRVTVDERHHKEPIKLGSAFI
TTVVPDGIWLKLEKLQWHQSRGS
>Unigene0035516
LVDFFDPILDNMMATAPVDLWRKIRPSSSPAFTAAKMRKMNNLIQDCARVTSEHLKNAAE
EEKDVDIKRFYGHYALDVIAKCAFGTELDSLEDANNEFVANASKAFSGGINLAVILFVLC
PALMRVLKPKILSPEPFRYFKEVCLAIIRERRRLQRRHEDFLQLMMDAQEGTLVDSAEDA
PGKESAETMDLDCNVNNGVTFVTRALSEDEALAQCVQFFVAAHDGISTVLACAAYLLALN
PEAQEKLCTEADECFTAHGNNPSLDVISKLPYLHCVISETMRLYPPAPRLDRTASKVYVM
GDTGIRVPKDSIVTVPIYAMHRDPEFFPDPDKFIPERFSPANAASIRPYSYLPFGAGPRN
CIGMRMALQTTKLCLLHSVHAVRFV
>Unigene0004477
MDLMAALTPVWVILAVTPGVLLYLYVTRNRNFWKNQNIRSEPFSIIFGATLKTFLNPVHE
VDLARYRKYGKLFGAFEMGKALLFVAEPKLVKQVLIDDFTSLPNRRTLDMNEPLLDNMMS
MASFEVWQKVRKGSAPAFSAKVLRKMNAFIEECALATTEHLKKAASNEEDVDVKQFFWDY
ALDVIAKCAFAMKLDSHSDPTNEFVTRSKQVLSQRFTPRLLVMILFPSIAKRFKIGPMKP

DEIDFFKSLGRNIVKDSKGTKAHDNFLRLLIEEKKEQLDKEREKVFDRDHRLFGIGSDTQ
QDALSSSEIKLAEDEALAQCIFFFLAGQQKAANAIGCTLYLLAIHQEVQDRLRKEVDECC
AIHGDRPGLDAITNLKYLHCVVSETLRLYPPVTRLERAPSEDYTLGDTGVKVTKSDLVVV
PVYAMQHDPQYFPEPSNFDPERFNDENVGSIQPYTYLPFGAGPRNCIAISFTLQAMKLAI
FHAIRNVQVVRTANTKVPLVFQNGFRPLTAENITLGIRKR
>Unigene0004053
QPLNEVVAKNYNRYGRVYGSYQGTVPTLVVGDTNILREVLVSKFKCFSDRTAGQRIGSDV
WRKSILNLSGEEWRKARGIFTPALTATQLKTIAARITTIAERMTHRVAEAAAKNKPVNIS
ELFDHTSLDTTAALNYSVDLDSEIDKDHPLMNCLEAIFGNMAGWKLLMLFLMPSVYKALQ
PDYPPKASTDVFKAFVAHMIEERTSRNKKEEDFLQLFMDADYDWDDAADGADNKNGKRMT
LDEITAQGIVFFIAGVESVSMSTTFTAYFLALHPECQDRVISEIDKAVTEGGMTYDTLQE
MHYLEASIKEAMRLTSPDPVIMRLCTEETTVAGIRFTPGMNVDIPLAGIHLDPEYFPEPE
KFTPERFLPENKDSIRPFTYMPFGAGPRNCAGSRLALLQAKAYLACLLRRFRLQVCSETM
IPLKYRPRRLFPFPDGPVVLRAVARGHAANGATTAS*TILR
>Unigene0074648
VMEQWIQRYGNVFGIYLGDKPFMVITDVDIIKECFIKAARVFQDRSMYGIDAEPFKSGLI
LLREEKWRKVRSVFNTCFTSVKVKKVSGLVDAAMTRFVQKFEEASRFGEIIEAHDTARRM
VLDLLTRTVLGRQVDCQTNSHDPALKSFEVIFKEADNTFFEGTFAYPGLRSLLLCIYPFT
SFCKALQKVMDDALRVLKERRSGESERKEDVLQHVVDAQQGIGDILPTTNANARSIDDTT
LLSNFAILLIAGFDTTSASLAFLLYQLAKHPEEQLKIRAEIAGKLRGKNISACEYTNTNL
KEQQDLEGEQRQENDNACSPIRPRGTDNPATGTVNARNQQARHTEVTRPSHTAFHQDIAK
NELDEDEVMQFERLDMVVREGLRLYPPIPITIMRDCTEDMTVSGQFIPAGMSVMSPPWHV
HRDADIWSEPNSFIPDRFLQKDRDIASSCYYPFGLGPRMCVAHRLGMVTLKTALYRIMRD
FEISLADGVPDPLPVSVEHVILNPAAAIKLKVKKSQL
>Unigene0073522
MLDFAIFAVSFVVFLLALVLYLYPSSAKQTTIPGLEPSDKKEGNVGDIVQAGGLQNFLIS
LHKEHGPIASFWIGTKLVVSIGKADLFKTQSHVFDKPAELFVLYRDVMGAGSIFFANGAE
ARKRRRLIDEVLTGKSLEMFLEPIEKLCSEVVMHLKDTPDDEHVPVYQYMYALCMKISTR
LLFGEYFFNDVEVLKFSRNFELCIKELEEIANGVVPDSNSPRTKKYEEAAKEMRALLVKA
LQKCKAGGDKVLLANVLNDPSVPEEQAVDDCVTFAIKAYSLVSAMTWMLYFLATHPELQD
TIAEEVKGTTDKTKGLSCQALSSMKSLQNSIKETLRTATVEPWAARCQDVDIDIAGHIIP
KKTPVIQALGVVLHEQENWKVPQRYSKSIF
